# Discovery of SD-965 as a Potent, Selective, and Efficacious STAT3 PROTAC Degrader

**DOI:** 10.1021/acs.jmedchem.5c03767

**Published:** 2026-03-23

**Authors:** Dimin Wu, Haibin Zhou, Longchuan Bai, Ranjan Kumar Acharyya, Hoda Metwally, Donna McEachern, Mi Wang, Jelena Tošović, Rohan Kalyan Rej, Meilin Wang, Bo Wen, Duxin Sun, Shaomeng Wang

**Affiliations:** † Department of Internal Medicine, Division of Hematology/Oncology, 1259 University of Michigan, Ann Arbor, Michigan 48109, United States; ‡ Department of Pharmacology, University of Michigan, Ann Arbor, Michigan 48109, United States; § Department of Medicinal Chemistry, College of Pharmacy, University of Michigan, Ann Arbor, Michigan 48109, United States; ∥ Department of Pharmaceutical Sciences, College of Pharmacy, University of Michigan, Ann Arbor, Michigan 48109, United States; ⊥ The Rogel Cancer Center, University of Michigan, Ann Arbor, Michigan 48109, United States

## Abstract

Signal transducer and activator of transcription 3 (STAT3) is a promising therapeutic target for human cancers and other human diseases. Herein, we report on the design, synthesis, and evaluation of novel STAT3 PROTAC degraders using high-affinity STAT3 ligands and cereblon ligands. Our study led to the discovery of SD-965 as a potent, selective, and efficacious STAT3 degrader. A single intravenous administration of SD-965 effectively induces rapid, complete, and durable depletion of STAT3 protein in mouse native and human xenograft tumor tissues with no depletion of other STAT proteins. SD-965 is capable of achieving tumor regression even with weekly administration in human leukemia and lymphoma xenograft models in mice without any signs of toxicity. SD-965 represents a promising STAT3 degrader for extensive evaluation for the treatment of human cancers and other human diseases.

## Graphical abstract

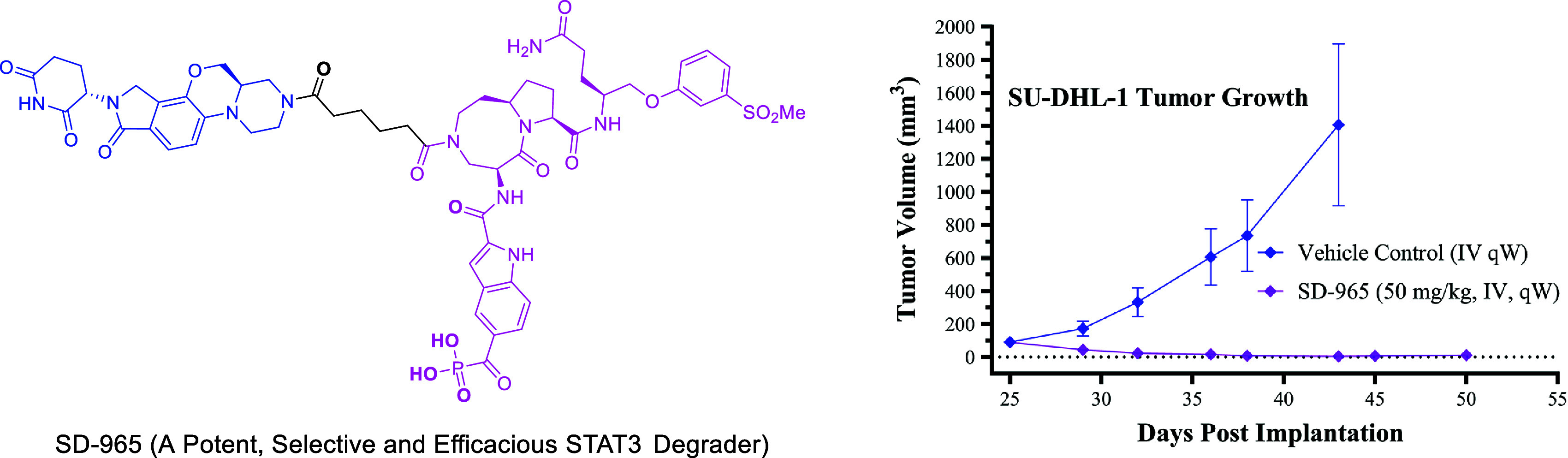

## Introduction

Signal Transducer and Activator of Transcription 3 (STAT3) is a member of the cytoplasmic transcription factor family that transmits signals from extracellular growth factors and cytokines to activate gene transcription.^1^ Overexpression and/or hyperactivation of STAT3 is observed in approximately 70% of human cancers,^2^ including but not limited to leukemias,^3,4^ lymphomas,^5–7^ and different forms of solid tumors.^8–11^ As a transcriptional factor, STAT3 plays a crucial role in the regulation of cell proliferation, apoptosis, metastasis, angiogenesis, immunosuppression, and inflammation in human cancers.^3,4,10–17^ Therefore, STAT3 has been considered as an attractive therapeutic target for human cancers and other human diseases.^2,18,19^

STAT3 consists of six domains, including the N-terminal domain (NTD), coiled-coil domain (CCD), DNA-binding domain (DBD), linker domain (LD), Src homology 2 (SH2) domain, and C-terminal domain (CTD).^20,21^ STAT3 is activated by phosphorylation of its tyrosine 705 residue located on its SH2 domain by Janus kinase (JAK) or other kinases. Upon phosphorylation, STAT3 forms a homodimer, which is then translocated from the cytosol to the nucleus to bind to its targeted DNAs for gene transcription. It has been proposed that STAT3 dimerization is essential for its gene transcriptional activity.^22–24^ Accordingly, small-molecule inhibitors have been developed to target the SH2 domain of STAT3 to block its dimerization. Unfortunately, many of the previously reported STAT3 inhibitors (Figure 1) lack desired potency and selectivity and clear cellular mechanisms of action, making STAT3 one of the classical undruggable targets for 3 decades.^25–29^

**Figure 1:**
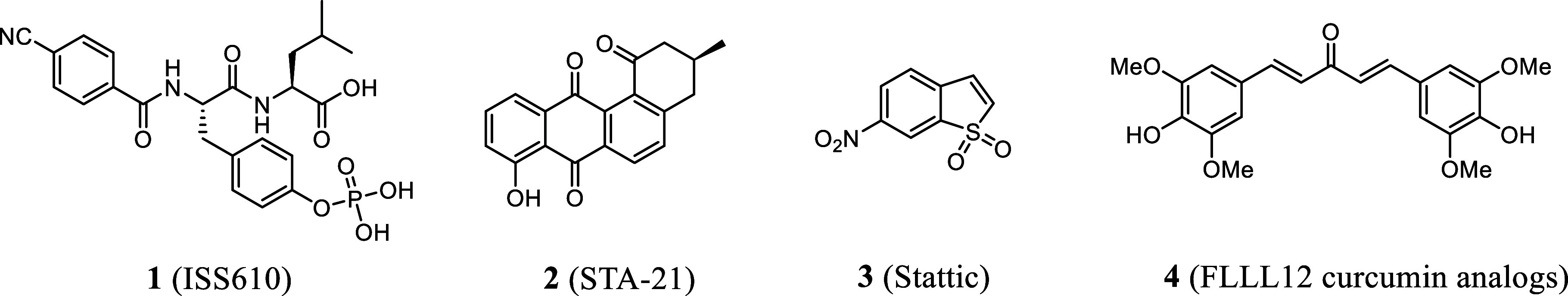
Previously reported, representative small-molecule STAT3 inhibitors.

In recent years, targeted protein degradation (TPD) using the proteolysis-targeting chimera (PROTAC) technology has become a powerful strategy to target those traditionally undruggable proteins.^30–32^ Our group reported SD-36 (Figure 2)^33,34^ as the first potent, selective, and efficacious PROTAC STAT3 degrader. We showed that while SD-36 is very potent and effective in the inhibition of STAT3 gene transcriptional activity, its corresponding inhibitor SI-109 is 1000-times less potent than SD-36 and only modestly effective. SD-36 was highly effective in inducing complete depletion of STAT3 in mouse native tissues and human xenograft tumor tissues.^33^ Impressively, weekly administration of SD-36 was capable of achieving complete and long-lasting tumor regression in mice without any signs of toxicity.^33^ Our data on SD-36 provided clear evidence that STAT3 can be successfully targeted by PROTAC technology.^33,34^ Our subsequent investigation of SD-36 led to the discovery of SD-91 (Figure 2),^35^ which has a better chemical stability than SD-36 in cells and *in vivo* and is similarly potent and efficacious as compared to SD-36. More recently, we reported the discovery of SD-436 (Figure 2),^36^ as another potent, selective, and highly efficacious PROTAC STAT3 degrader, which has an improved *in vivo* potency compared to SD-36 and SD-91.

**Figure 2:**
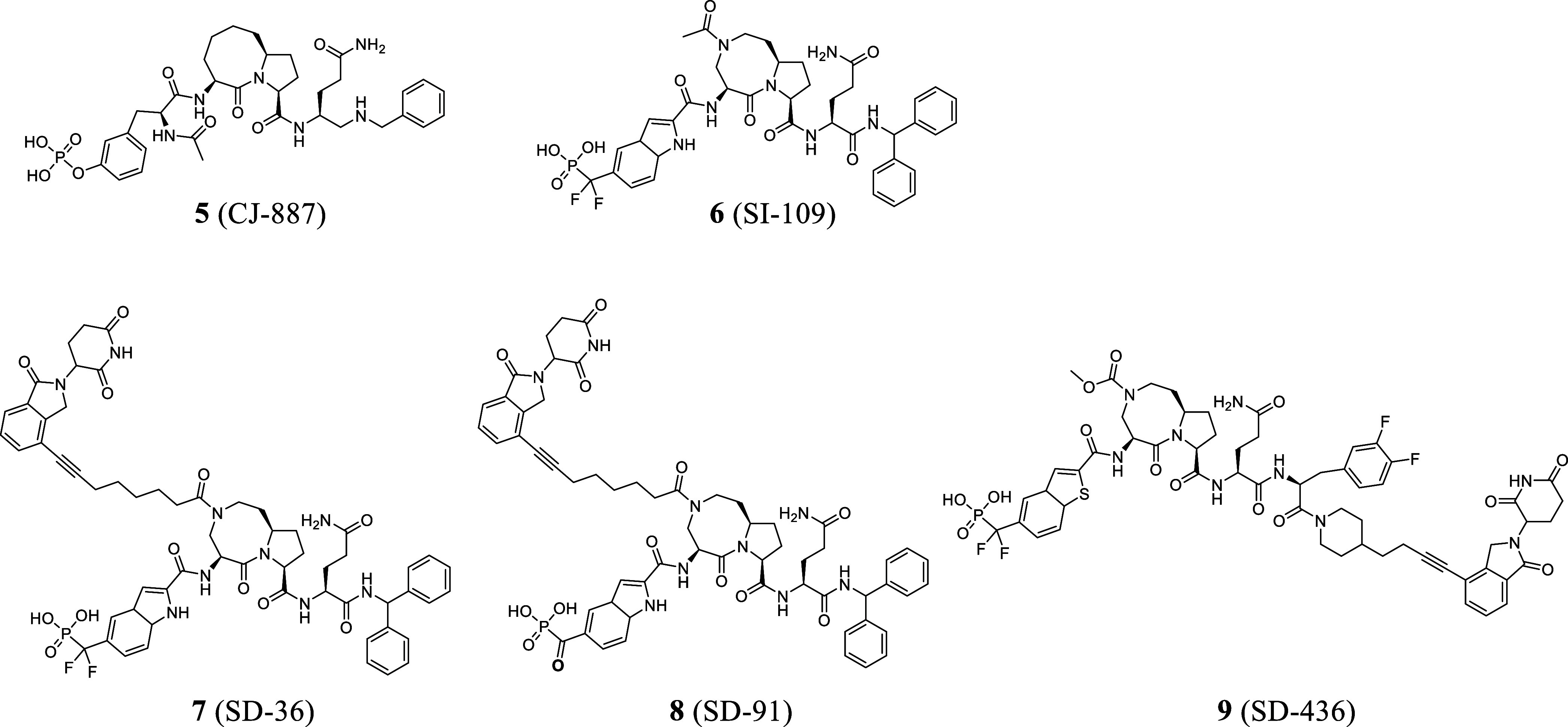
STAT3 ligands and STAT3 degraders reported by our group.

In the development of SD-36, SD-91, and SD-436, we have employed a lenalidomide analogue as the cereblon ligand. In the present study, we reported the design, synthesis, and evaluations of new STAT3 degraders using a series of high-affinity cereblon ligands and through optimization of the STAT3 ligand portion and the linker. Our study resulted in the discovery of a number of STAT3 degraders, which are more potent than SD-36, SD-91, and SD-436. Among them, SD-965 displays the best *in vivo* activity and is a potent, selective, and efficacious STAT3 degrader.

## Results and Discussion

### Design of New PROTAC STAT3 Degraders Using a High-Affinity CRBN Ligand

Recently, our group reported a series of cereblon ligands with high binding affinities and good pharmacokinetic (PK) profiles.^37–42^ Among our reported cereblon ligands, **11** (RR-11055) has a higher binding affinity to cereblon than thalidomide and lenalidomide.^40^ Additionally, RR-11055 has excellent microsomal and plasma stability in different species.^40^ We decided to employ RR-11055 for the design of new STAT3 degraders.

In our previous study, SI-203 was one of the most potent STAT3 inhibitors (*K*_i_ = 6 nM) identified.^36^ Using SI-203 as the STAT3 ligand and RR-11055 as the cereblon ligand, we designed and synthesized a series of degraders with linkers of various lengths. We evaluated these potential degraders for their ability to degrade STAT3 protein in a sensitive and quantitative HiBiT (Highly Improved Bioluminescent Imaging Tag) assay, with the data summarized in Table 1.

**Table 1:**
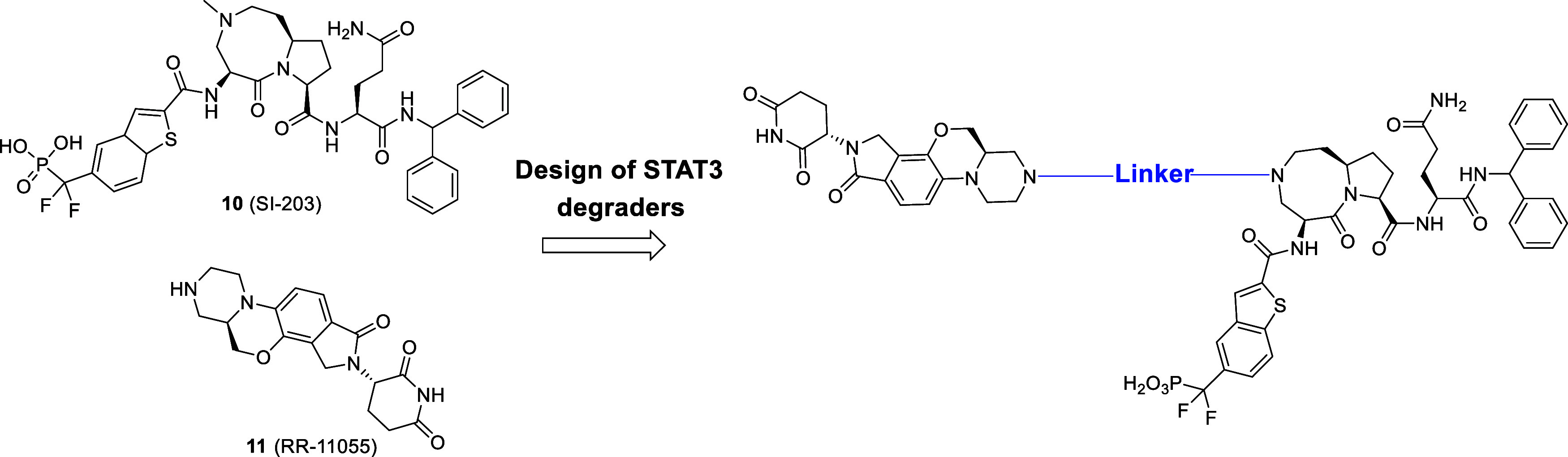

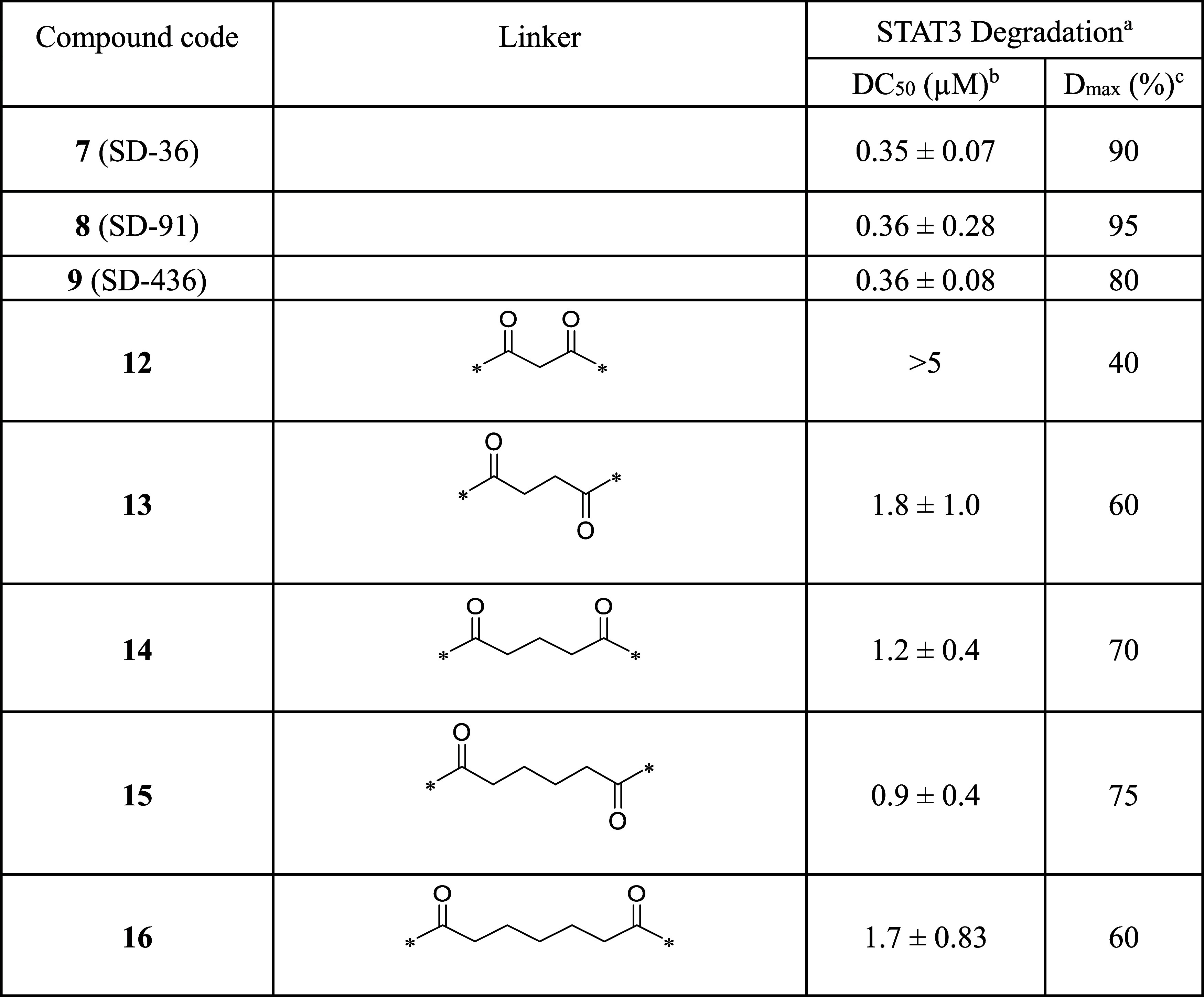
STAT3 Degraders with Linkers of Various Lengths

a STAT3 degradation potency was tested in the STAT3 HiBiT assay. Cells were treated for 24 h.

b The concentration needed for the reduction of STAT3 protein by 50%.

c Maximal degradation achieved up to 5 μM. SD-36, SD-91, and SD-436 were included as the controls.

Our data showed that compound **12**, which has a linker containing one carbonyl group on either side and one methylene unit, is a weak and ineffective STAT3 degrader (DC_50_ > 5 μM and *D*_max_ = 40%). Increasing the linker length by one to four methylene units between the two carbonyl groups yielded compounds **13**–**16**, which attained DC_50_ values of 1.8, 1.2, 0.9, and 1.7 μM, respectively, and *D*_max_ values of 60–75%. Among this series of degraders, compound **15** is the most potent (DC_50_ = 0.9 μM) and effective (*D*_max_ = 75%). In the same assay, SD-36, SD-91, and SD-436 have DC_50_ values of 0.35–0.36 μM and *D*_max_ = 80–90%. Hence, compound **15** is less potent and effective than those three previously reported STAT3 degraders.

### Optimization of the STAT3 Ligand in STAT3 Degraders

Analysis of the cocrystal structure^33^ of SD-36 in complex with STAT3 showed that the pro-(*S*)-phenyl group of the STAT3 ligand portion interacts with a surface pocket formed by I659, M660, and L666 residues of STAT3, whereas the pro-(*R*)-phenyl group has no specific interactions with STAT3 (Figure S5). We reasoned that further optimization of the interactions of the pro-(*S*)-phenyl in the STAT3 ligand may improve the degradation potency (DC_50_) and effectiveness (*D*_max_) of the resulting STAT3 degraders (Tables 2 and 3).

**Table 2:**
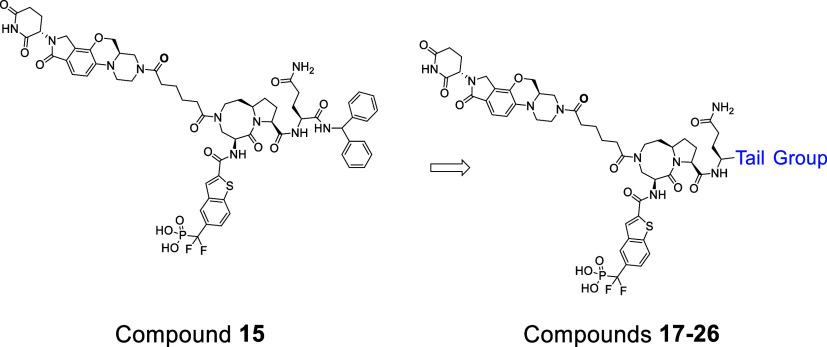

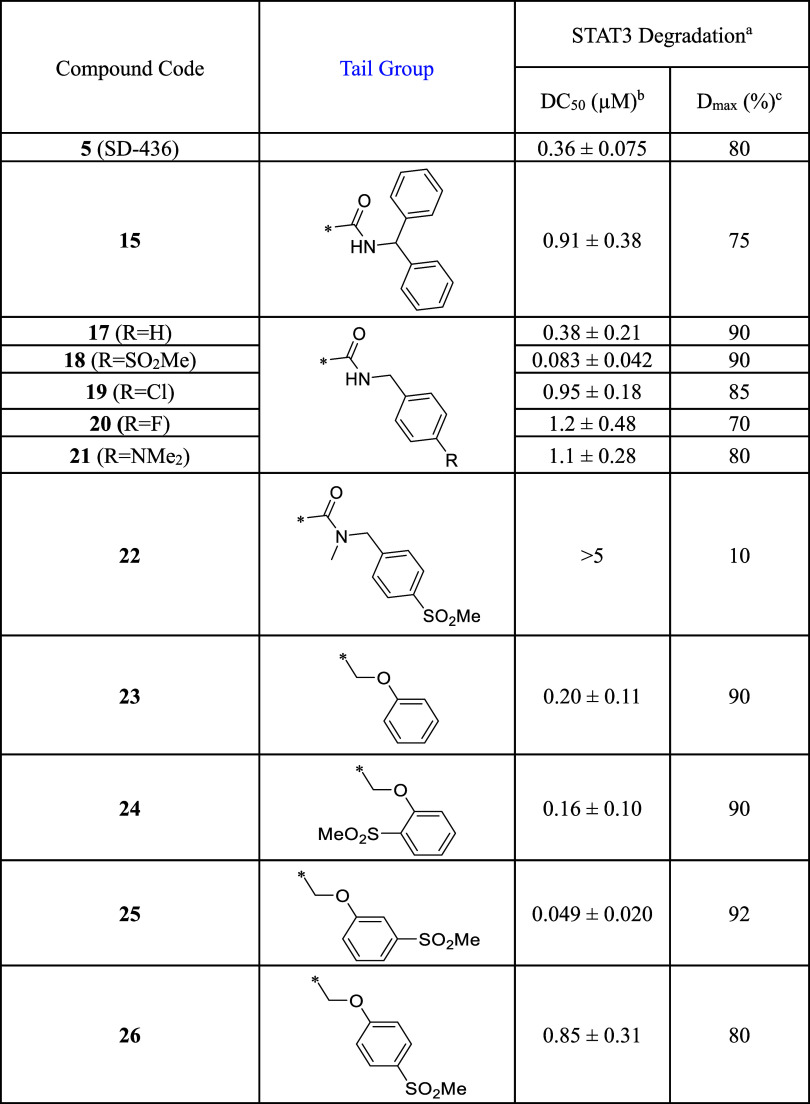
STAT3 Degraders with Modifications on the “Tail” Group

a STAT3 degradation potency was tested in the STAT3 HiBiT assay. Cells were treated for 24 h.

b The concentration needed for the reduction of STAT3 protein by 50%.

c Maximal degradation achieved up to 5 μM. SD-36, SD-91, and SD-436 were included as the controls.

**Table 3:**
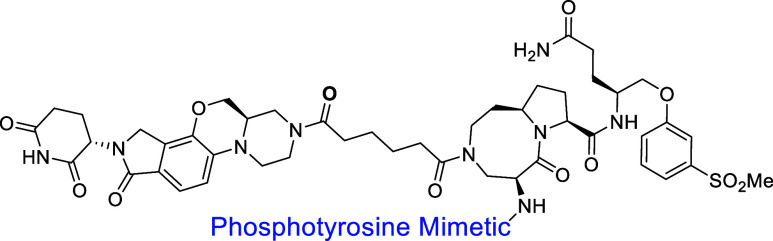

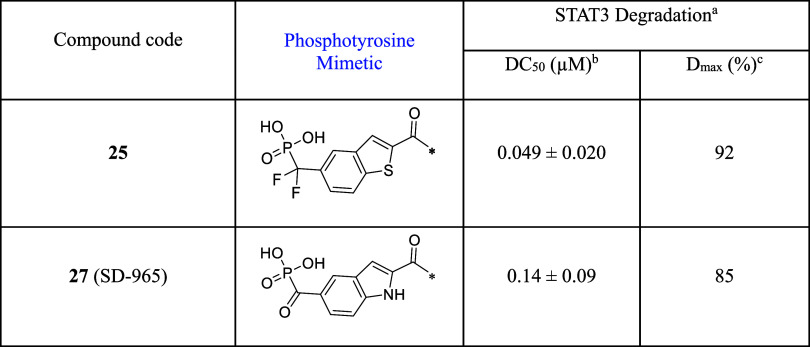
Design of a STAT3 Degrader with a Different Phosphotyrosine Mimetic

a STAT3 degradation potency was tested in the STAT3 HiBiT assay. Cells were treated for 24 h.

b The concentration needed for the reduction of STAT3 protein by 50%.

c Maximal degradation achieved up to 5 μM. SD-36, SD-91, and SD-436 were included as the controls.

To facilitate the optimization, we removed the pro-(*R*)-phenyl group, which resulted in compound **17**. Surprisingly, compound **17** attained DC_50_ = 0.38 μM and *D*_max_ = 90%. In comparison, compound **17** is more potent and effective than compound **15**. Encouraged by the degradation activity for compound **17**, we installed different substitutions on the phenyl group in compound **17**, which led to compounds **18**–**21**. Compound **18** with a *p*-methylsulfone substitution achieved DC_50_ = 0.083 μM and *D*_max_ = 90%, which is more potent than compound **17**. In comparison, compounds **19**, **20**, and **21** with *p*-Cl, *p*-F, and *p*-NMe_2_ substitutions are similarly potent compared to compound **17**.

We synthesized compound **22** by the installation of a methyl substitution onto the amino group in compound **18**. Compound **22** is a weak and ineffective STAT3 degrader (DC_50_ > 5 μM and *D*_max_ = 10% at 5 μM). Because the corresponding amino group in SD-36 has no specific interactions with STAT3, the much reduced degradation potency and effectiveness for compound **22** compared to compound **18** is likely attributed to altered conformations of the *p*-(methylsulfonyl)­phenyl group, which may weaken the interactions with I659, M660, and L666 residues in STAT3.

In compound **17**, the amide moiety in the tail portion of the molecule has no specific interactions. We investigated whether this amide group can be replaced with other groups without loss of STAT3 degradation. For synthetic feasibility considerations, this amide group can be converted into an ether. Our modeling suggested that a direct connection of the phenyl group with the ether group can achieve effective interactions with STAT3. Accordingly, we synthesized compound **23**, which attained DC_50_ = 0.20 μM and *D*_max_ = 90%. Hence, compound **23** is similarly potent and effective compared to compound **17**.

Because the methylsulfonyl substitution on the phenyl group enhances the STAT3 degradation potency for compound **18** over compound **17**, we synthesized compounds **24**–**26** with a methylsulfonyl substitution in ortho, meta-, or para-position, respectively, on the phenyl group in compound **23**. Compound **24** with *o*-SO_2_CH_3_ substitution is similarly potent and effective compared to compound **23**. Compound **25** with *m*-SO_2_CH_3_ substitution achieves DC_50_ = 0.049 μM and *D*_max_ = 92% and is therefore 4-times more potent than compound **23**. Compound **26** with *p*-SO_2_CH_3_ substitution shows DC_50_ = 0.85 μM and *D*_max_ = 80% and is weaker and less effective than compound **23**.

In our previous study, we have reported several phosphotyrosine mimetics for the development of STAT3 degraders.^36^ We employed the same phosphotyrosine mimetic used in SD-436 for the design of compound **25**. Additionally, we employed (1*H*-indole-5-carbonyl)­phosphonic acid as the phosphotyrosine mimetic for the design of SD-91 as a potent, selective, and efficacious STAT3 degrader with excellent chemical stability. We next replaced (benzo­[*b*]­thiophen-5-yldifluoromethyl)­phosphonic acid in compound **25** with (1*H*-indole-5-carbonyl)­phosphonic acid, which yielded compound **27** (SD-965). Compound **27** attained DC_50_ = 0.14 μM and *D*_max_ = 85%, which is 3-times weaker than compound **25** based on their DC_50_ values.

### Design of STAT3 Degraders Using Different Cereblon Ligands

Thalidomide and lenalidomide, as well as their analogues, have been extensively employed for the development of PROTAC degraders for different proteins in the past decade. In the past few years, our laboratory and other groups have reported the design of new cereblon ligands, which achieve higher binding affinities to cereblon than thalidomide and lenalidomide and display excellent absorption, distribution, metabolism, and excretion (ADME) properties. We next designed and synthesized a series of STAT3 degraders based on SD-965 using a number of previously reported cereblon ligands, with the data summarized in Table 4.

**Table 4:**
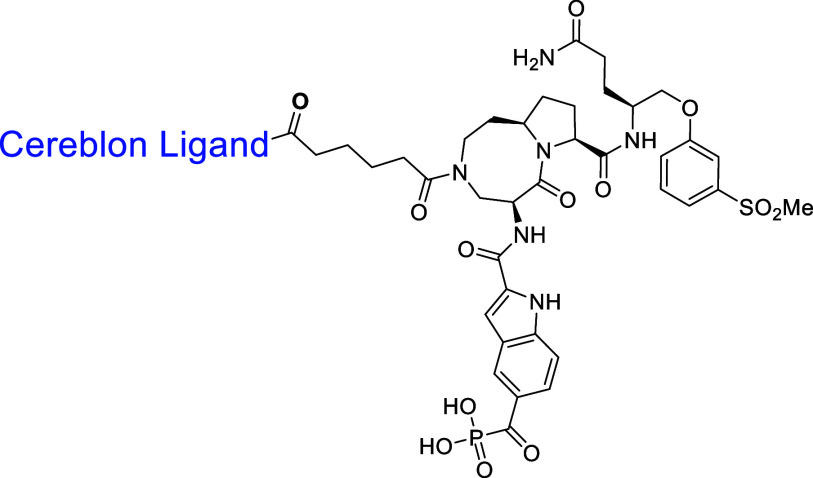

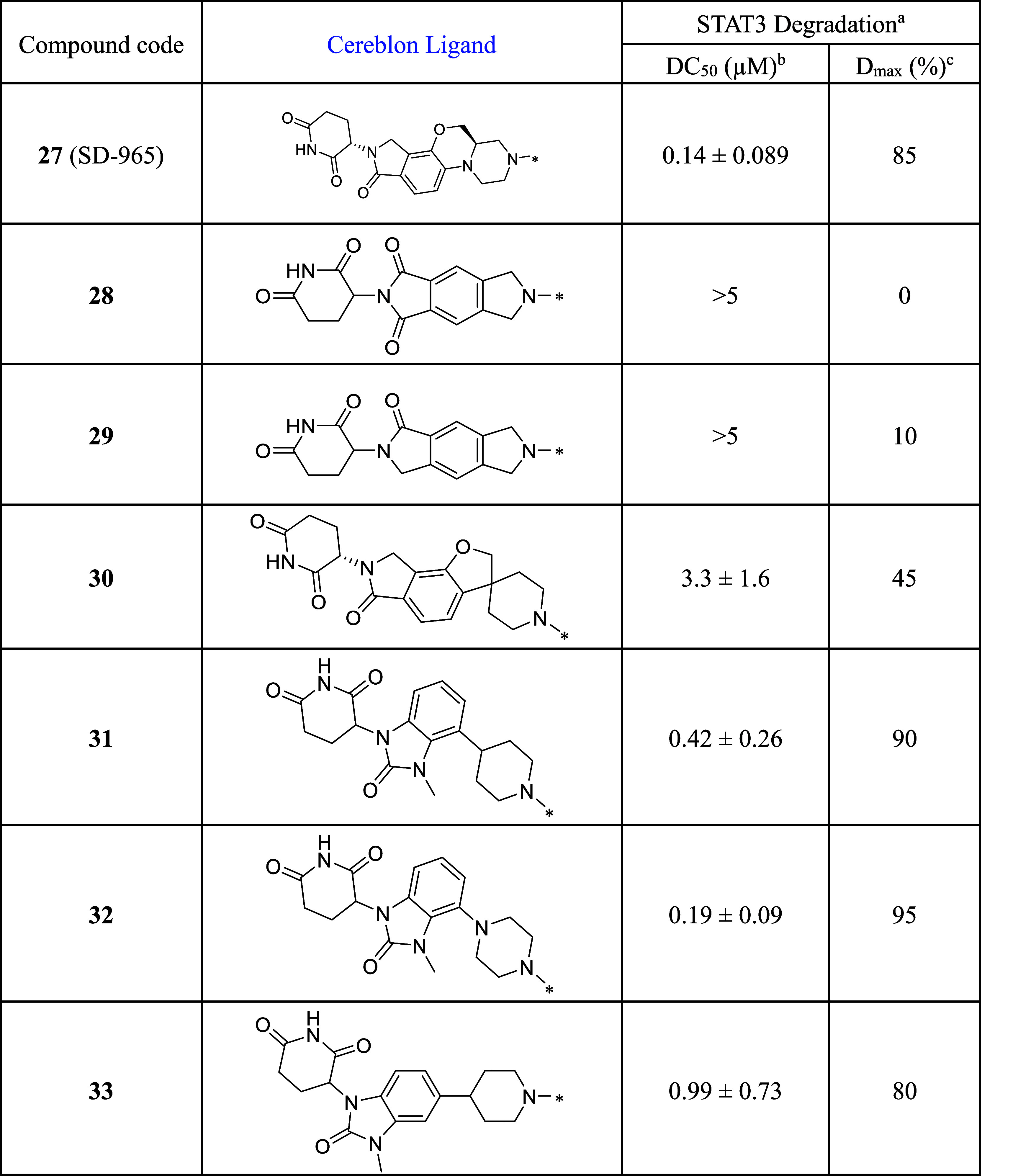

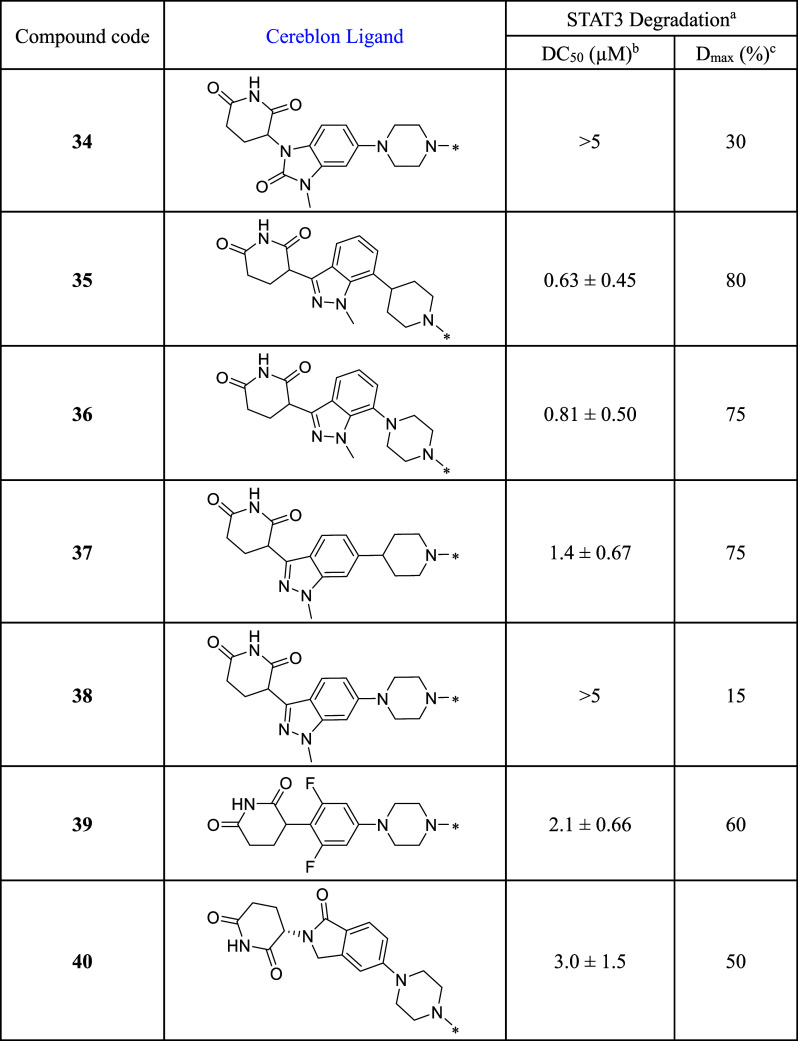
STAT3 Degraders Designed Using Different Cereblon Ligands

a STAT3 degradation potency was tested in the STAT3 HiBiT assay. Cells were treated for 24 h.

b The concentration needed for the reduction of STAT3 protein by 50%.

c Maximal degradation achieved up to 5 μM. SD-36, SD-91, and SD-436 were included as the controls.

Compounds **28** and **29**, designed using two tricyclic cereblon ligands previously reported from our laboratory,^37–39^ were ineffective in inducing STAT3 degradation up to 5 μM. Compound **30**, designed using a spiro cereblon ligand we have previously employed for the development of potent and orally efficacious ER degraders,^41^ was only a weak and ineffective STAT3 degrader (DC_50_ > 5 μM and *D*_max_ = 45%).

Kymera Therapeutics^43^ has disclosed 3-(3-methyl-2-oxo-2,3-dihydro-1*H*-benzo­[*d*]­imidazol-1-yl)­piperidine-2,6-dione as a novel cereblon ligand. Employing this cereblon ligand, Kymera has reported the development of KT-474 as a highly potent and efficacious MDM2 degrader for clinical development.^44^ We designed and synthesized four STAT3 degraders (compounds **31**–**34**) using this cereblon ligand. Compound **31** with a piperidinyl group in the ortho position of the phenyl group attained DC_50_ = 0.42 μM and *D*_max_ = 90%. Replacement of the piperidinyl group in compound **31** with a piperazinyl group yielded compound **32**, which displayed DC_50_ = 0.19 μM and *D*_max_ = 95%. Compounds **33** and **34** were obtained by changing the piperidinyl group in compound **31** or the piperazinyl group in compound **32** from the ortho position to the meta position on the phenyl group. Compound **33** had DC_50_ = 0.99 μM and *D*_max_ = 80% and is therefore weaker and less effective than compound **31**. Compound **34** was a very weak and ineffective degrader (DC_50_ > 5 μM and *D*_max_ = 30%). Hence, our degradation data for compounds **31**–**34** highlight the importance of the tethering position to the cereblon ligand for achieving potent and effective STAT3 degradation.

3-(1-Methyl-1*H*-indazol-3-yl)­piperidine-2,6-dione has been used in the development of PROTAC degraders, including an orally bioavailable BCL6 degrader, BMS-986458, currently in clinical development.^45^ We have employed this cereblon ligand for the development of a highly potent and orally efficacious MDM2 degrader, MD-4251.^46^ We designed and synthesized four STAT3 degraders (compounds **35**–**38**) using this cereblon ligand. Compound **35** with a piperidinyl group connecting to the ortho position of the phenyl group in the cereblon ligand achieved DC_50_ = 0.63 μM and *D*_max_ = 80%. In comparison, compound **36** with a piperazinyl group connecting to the ortho position of the phenyl group in the cereblon ligand attained DC_50_ = 0.81 μM and *D*_max_ = 75%, which is similarly potent and effective as compound **35**. Compound **37** with a piperidinyl group connecting to the meta position of the phenyl group in the cereblon ligand displayed DC_50_ = 1.4 μM and *D*_max_ = 75% and is therefore 2-times less potent than compound **35**. Compound **38** with a piperazinyl group connecting to the meta position of the phenyl group in the cereblon ligand is a very weak and ineffective STAT3 degrader (DC_50_ > 5 μM and *D*_max_ = 15%).

3-(2,6-Difluorophenyl)­piperidine-2,6-dione has been identified as a structurally simple cereblon ligand.^47^ We synthesized compound **39** using this cereblon ligand, which attains DC_50_ = 2.1 μM and *D*_max_ = 60%. We synthesized compound **40** using a lenalidomide analogue, which has been successfully used for the design of ARV-471,^48^ an orally bioavailable ER degrader. Compound **40** displayed DC_50_ = 3.0 μM and *D*_max_ = 50% and is therefore a modestly potent and effective STAT3 degrader.

### Evaluation of Representative Potent STAT3 Degraders for Their Growth Inhibition in Cancer Cell Lines

We previously showed that our first-in-class STAT3 degrader SD-36 was highly effective in the inhibition of cell growth in the MOLM-16 leukemia cell line and in the SU-DHL-1 anaplastic large cell lymphoma cell line, which have highly activated STAT3.^33,34^ We selected several of the most potent STAT3 degraders identified from this study and evaluated their cell growth inhibition in these two cell lines. We included SD-36, SD-91, and SD-436, three of our previously reported STAT3 degraders, as the controls in our evaluation, with the data summarized in Table 5.

**Table 5:** Cell Growth Inhibitory Activities of STAT3 Degraders in Two Cancer Cell Lines

	cell growth inhibition (IC_50_ ± SD)	
compound code	MOLM-16 (nM)	SU-DHL-1 (nM)
SD-36	4.1 ± 1.4	809 ± 155
SD-91	1.2 ± 0.4	453 ± 75
SD-436	6.5 ± 1.9	929 ± 139
**27** (SD-965)	1.3 ± 0.2	260 ± 63
**25** (SD-964)	0.3 ± 0.1	181 ± 36
**31**	45 ± 12	2147 ± 312
**32**	14.4 ± 2.4	574 ± 81

The cell growth inhibition data showed that SD-965 and **25** achieved IC_50_ values of 1.3 and 0.3 nM, respectively, in the MOLM-16 leukemia cell line. SD-965 and **25** attained IC_50_ values of 260 and 181 nM, respectively, in the SU-DHL-1 lymphoma cell line. Compounds **31** and **32** are much weaker than SD-965 and **25** in the inhibition of cell growth in the MOLM-16 cell line and less potent than SD-965 and **25** in the SU-DHL-1 cell line.

In comparison, SD-965 is more potent than SD-36 and SD-436 in both cell lines. Compound **25** is the most potent among this series of STAT3 degraders in both cell lines. Hence, our cell growth data demonstrated that **25** and SD-965 are potent and promising STAT3 degraders for further *in vivo* evaluations.

### Pharmacodynamic (PD) Evaluation of **25** and SD-965 in Mice

Toward our goal of identifying new and efficacious STAT3 degraders, we evaluated **25** and SD-965 (compound **27**) for their pharmacodynamic (PD) effect in mice bearing SU-DHL-1 xenograft tumors.

Our PD data (Figure 3) showed that a single, intravenous administration of both **25** and SD-965 at 10 mg/kg reduced STAT3 protein by 90% in the SU-DHL-1 human xenograft tumor tissue, as well as in mouse spleen and liver native tissues at the 6 h time point. In mice treated with **25**, the levels of STAT3 protein rebounded to 50–60% of the control at the 24 h time point in the liver tissues, but remained low (<20%) in the tumor and spleen tissues. The levels of STAT3 protein were much higher in liver, spleen, and tumor tissues at the 48 h time point as compared to the levels at the 6 h time point. In comparison, in mice treated with SD-965, the levels of STAT3 protein remained at low levels (<10%) in all three tissues at 6 and 24 h time points. While the levels of STAT3 protein increased at the 48 h time point as compared to those at earlier time points in all three tissues in mice treated with SD-965, the levels were still much lower than those treated with **25** at the 48 h time point in each tissue. Hence, our PD data demonstrated that while both **25** and SD-965 are very effective in reducing the levels of STAT3 at 10 mg/kg intravenous administration, SD-965 achieves more durable depletion of STAT3 protein in mouse native tissues and in the SU-DHL-1 human xenograft tumor tissue. Based on the PD data, we performed further evaluation on SD-965.

**Figure 3:**
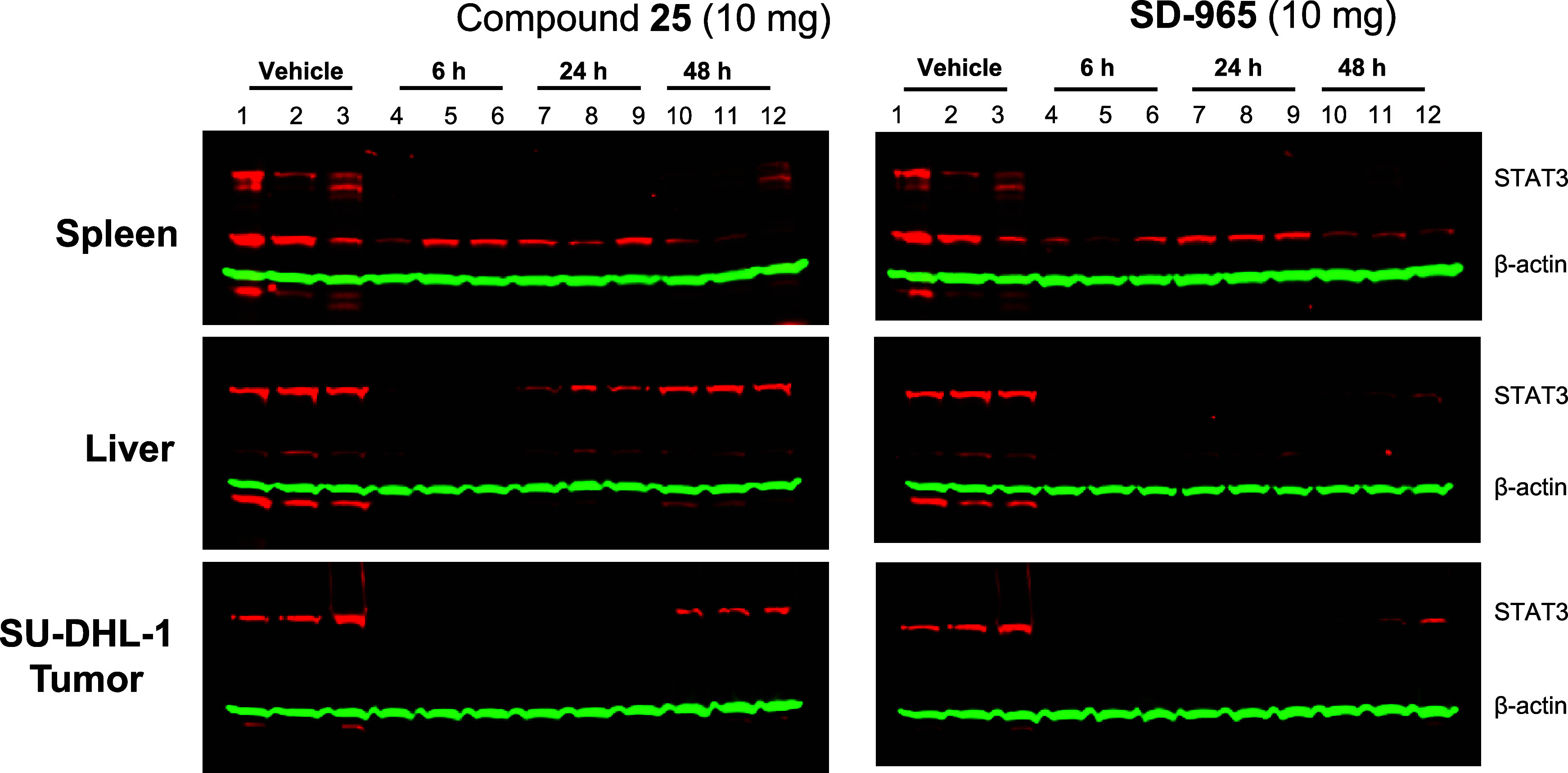
Pharmacodynamics analysis of STAT3 protein levels for **25** and SD-965 (compound **27**) in SU-DHL-1 xenograft tumors in mice. Mice were administered a single intravenous dose of 10 mg/kg with either **25** or SD-965. Mice were sacrificed at the indicated time points, and SU-DHL-1 xenograft tumor tissues and mouse spleen and liver tissues were collected for Western blot analysis.

### Evaluation of the Degradation Selectivity of SD-965 in Cells

We next evaluated the degradation selectivity of SD-965 in human peripheral blood mononuclear cells (PBMCs) and the SU-DHL-1 cell line, with the data summarized in Figure 4.

**Figure 4:**
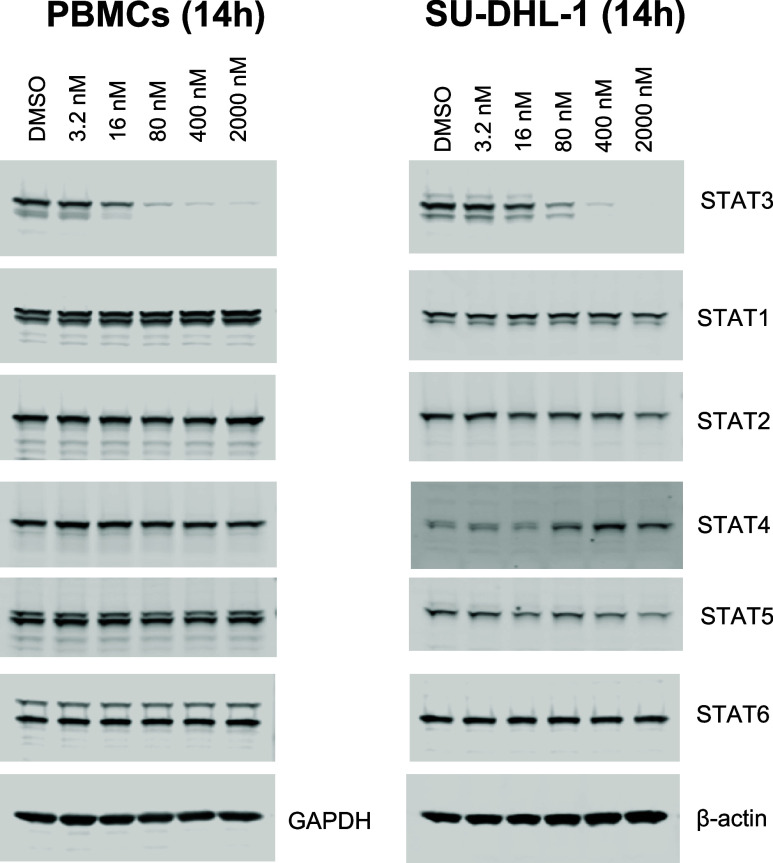
Evaluation of the degradation selectivity of SD-965 using Western blotting analysis in different cell lines. (A) Western blot analysis of STAT proteins in the PBMC cell line treated with SD-965 at 3.2–2000 nM for 14 h. (B) Western blot analysis in SU-DHL-1 cells treated with SD-965 at 3.2–2000 nM concentration for 14 h.

Human peripheral blood mononuclear cells (PBMCs) express all of the STAT members (Figure 4A). SD-965 was very effective in reducing the level of STAT3 protein at concentrations as low as 16 nM and reached a *D*_max_ value of >90%. SD-965 has no significant effect on other STAT proteins up to 2 μM, the highest concentration evaluated.

In the SU-DHL-1 cell line (Figure 4B), SD-965 attained DC_50_ = 80 nM and *D*_max_ > 90% for STAT3. SD-965 had no obvious effect on the levels of STAT1 and STAT6 proteins and modestly reduced the levels of STAT2 and STAT5 proteins at 2 μM. It increased the levels of STAT4 protein in the SU-DHL-1 cells in a dose-dependent manner, consistent with our previous reports^33–36^ for the effect of STAT3 degraders on this specific cell line.

The SU-DHL-1 cell line has a high level of pSTAT3 (Y705). We evaluated SD-965 for its ability to reduce total STAT3 and pSTAT3 (Y705) in the SU-DHL-1 cell. Our data (Figure 5A) showed that SD-965 effectively induced degradation of both total STAT3 and pSTAT3 (Y705) proteins.

**Figure 5:**
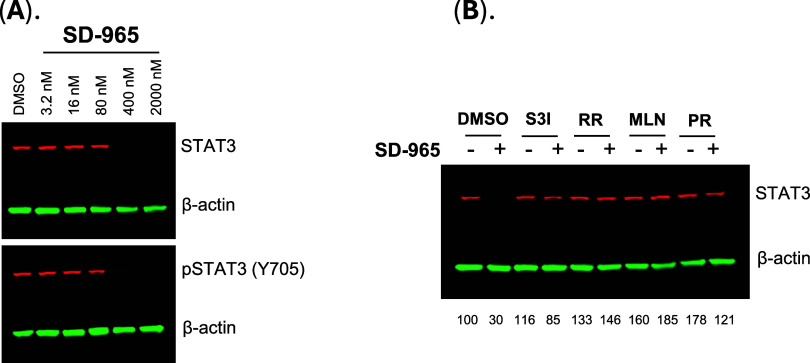
Further evaluation of SD-965 for its degradation activity and mechanism of action in SU-DHL-1 cells. (A) SD-965 effectively induced degradation of total STAT3 and pSTAT3 (Y705) proteins in SU-DHL-1 cells. SU-DHL-1 cells were treated as indicated for 6 h for Western blot analysis. (B) Degradation of STAT3 by SD-965 in SU-DHL-1 cells was effectively blocked by a STAT3 ligand (S3I-1655), a cereblon ligand (RR-11055), a selective NEDD8-activating enzyme inhibitor (MLN-4924), and a proteasome inhibitor (PR-171). SU-DHL-1 cells were pretreated with S3I-1655 (S3I, 40 μM) for 3 h, RR-11055 (RR, 20 μM), MLN-4924 (MLN, 0.5 μM), or PR-171 (PR, 0.2 μM) for 45 min, followed by treatment with SD-965 (0.5 μM) for an additional 4 h. Cells were then collected for Western blot analysis.

We evaluated SD-965 for its degradation mechanism in SU-DHL-1 cells. Our data (Figure 5B) demonstrated that STAT3 degradation induced by SD-965 was effectively blocked by a STAT3 inhibitor, a cereblon ligand, a selective NEDD8-activating enzyme inhibitor, and a proteasome inhibitor. Hence, our data confirmed that SD-965 is a *bona fide* PROTAC degrader of STAT3.

Hence, SD-965 is a potent and *bona fide* STAT3 degrader and achieves an excellent degradation selectivity for STAT3 over other STAT proteins.

### Evaluation of the Degradation Selectivity of SD-965 on a Global Level

We further evaluated the cellular degradation selectivity of SD-965 through an unbiased proteomics analysis, with the data summarized in Figure 6.

**Figure 6:**
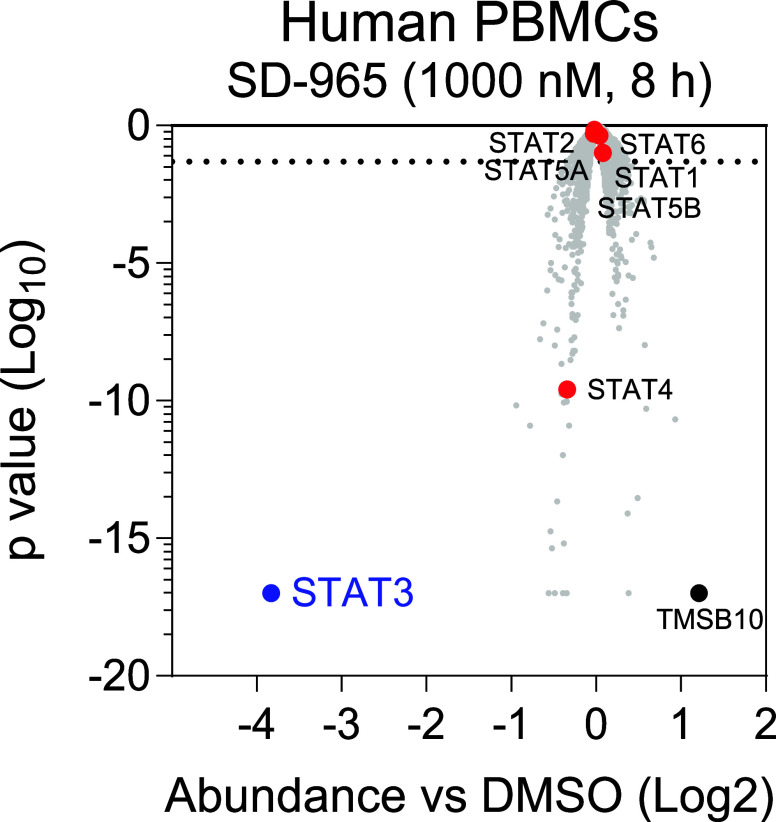
Multiplexed quantitative proteomics analysis of SD-965 for its degradation selectivity. Human PBMC cells were treated with SD-965 at 1 μM for 8 h.

To identify the potential off-target effect, we treated the human PBMC cells with SD-965 at a high concentration of 1 μM for 8 h. Our data showed that SD-965 effectively reduced the levels of STAT3 protein and is highly selective over other 7000 proteins analyzed.

### Profiling SD-965 for ADME and Pharmacokinetics

We evaluated SD-965 for its microsomal and plasma stability in mouse, rat, dog, monkey, and human species (Table 6). Our data showed that SD-965 has excellent microsomal and plasma stability.

**Table 6:** Microsome and Plasma Stability of SD-965

species	mouse	rat	dog	monkey	human
plasma stability *T*_1/2_ (min)	>120	>120	>120	>120	>120
microsome stability *T*_1/2_ (min)	>120	>120	>120	>120	>120

We evaluated SD-965 for its pharmacokinetics (PK) in mice, with data summarized in Table 7. SD-965 demonstrates a moderate half-life of 1.4 h, a slow clearance (CL = 496 mL/h/kg), a high plasma exposure (AUC = 4034 (h·ng)/mL), and a modest volume of distribution (*V*_ss_ = 530 mL/kg) with 2 mg/kg intravenous administration.

**Table 7:** Summary of Mouse PK Parameters for SD-965^a^

compound	species	IV (mg/kg)	*T*_1/2_ (h)	AUC_(0–24h)_ (h·ng/mL)	*V*_ss_ (mL/kg)	Cl (mL/h/kg)
SD-965	mice	2.00	1.44 ± 0.44	3976 ± 82	530 ± 50	496 ± 9

a The definitions are as follows: IV, intravenous administration; *T*_1/2_, elimination half-life; AUC, area-under-the-curve; *V*_ss_, volume of distribution at steady state; Cl, clearance. Three mice were used in the PK study.

### Evaluation of SD-965 for Its Antitumor Activity in the SU-DHL-1 Lymphoma Xenograft Model

Based on the promising PD data, we evaluated SD-965 for its antitumor activity in the SU-DHL-1 lymphoma xenograft tumor model, with the data summarized in Figure 7.

In the first efficacy experiment, we evaluated SD-965 for its antitumor activity dosed at 25 mg/kg weekly for 3 weeks (Figure 7A,B). SD-965 effectively inhibited tumor growth and induced a maximum tumor regression by 58% (Figure 7A). SD-965 was well tolerated and in fact induced a weight gain of 13% at the end of the experiment (Figure 7B).

**Figure 7:**
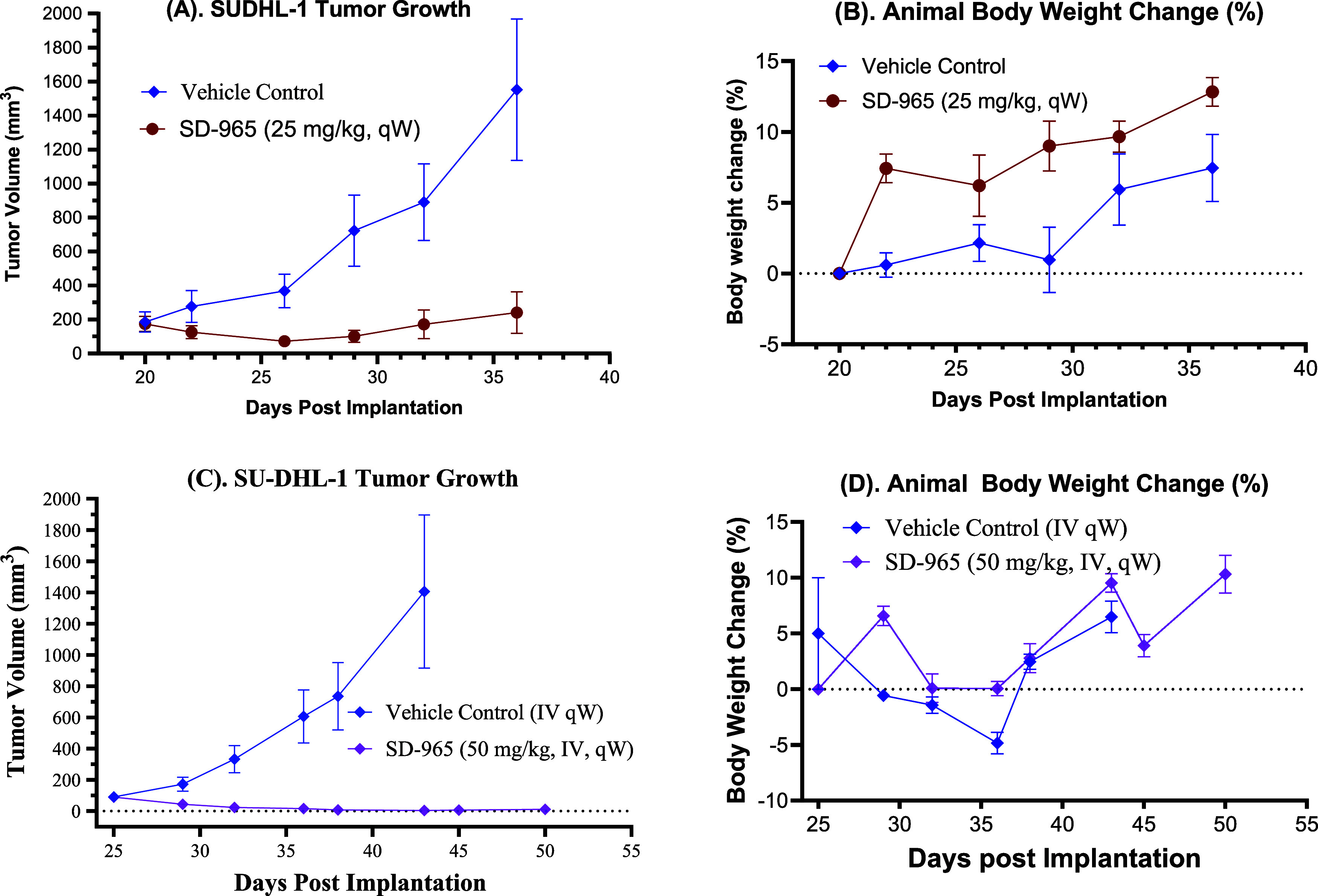
Antitumor activity of SD-965 in the SU-DHL-1 xenograft tumor model. In the first efficacy experiment (A, B), SD-965 was dosed weekly at 25 mg/kg for 3 weeks, and in the second efficacy experiment (C, D), SD-965 was dosed at 50 mg/kg weekly for 4 weeks.

Although SD-965 was effective in inducing tumor regression in the SU-DHL-1 xenograft tumor model at 25 mg/kg weekly intravenous administration, it did not achieve complete tumor regression. We further evaluated SD-965 at a higher dose in the SU-DHL-1 lymphoma xenograft tumor model, with the data summarized in Figure 7C,D. Our efficacy data (Figure 7C) showed that weekly intravenous administration of SD-965 at 50 mg/kg induced rapid tumor regression. SD-965 reduced the tumor volume by 52% on day 5 after the first dose and by 96% on day 16, with 60% of mice without palpable tumors. SD-965 did not induce animal weight loss or other signs of toxicity (Figure 7D).

Based on the promising antitumor activity of SD-965 in the SU-DHL-1 tumor model, we further evaluated its pharmacodynamics and antitumor activity in the MOLM-16 myeloid leukemia model in mice, with the data summarized in Figure 8.

**Figure 8:**
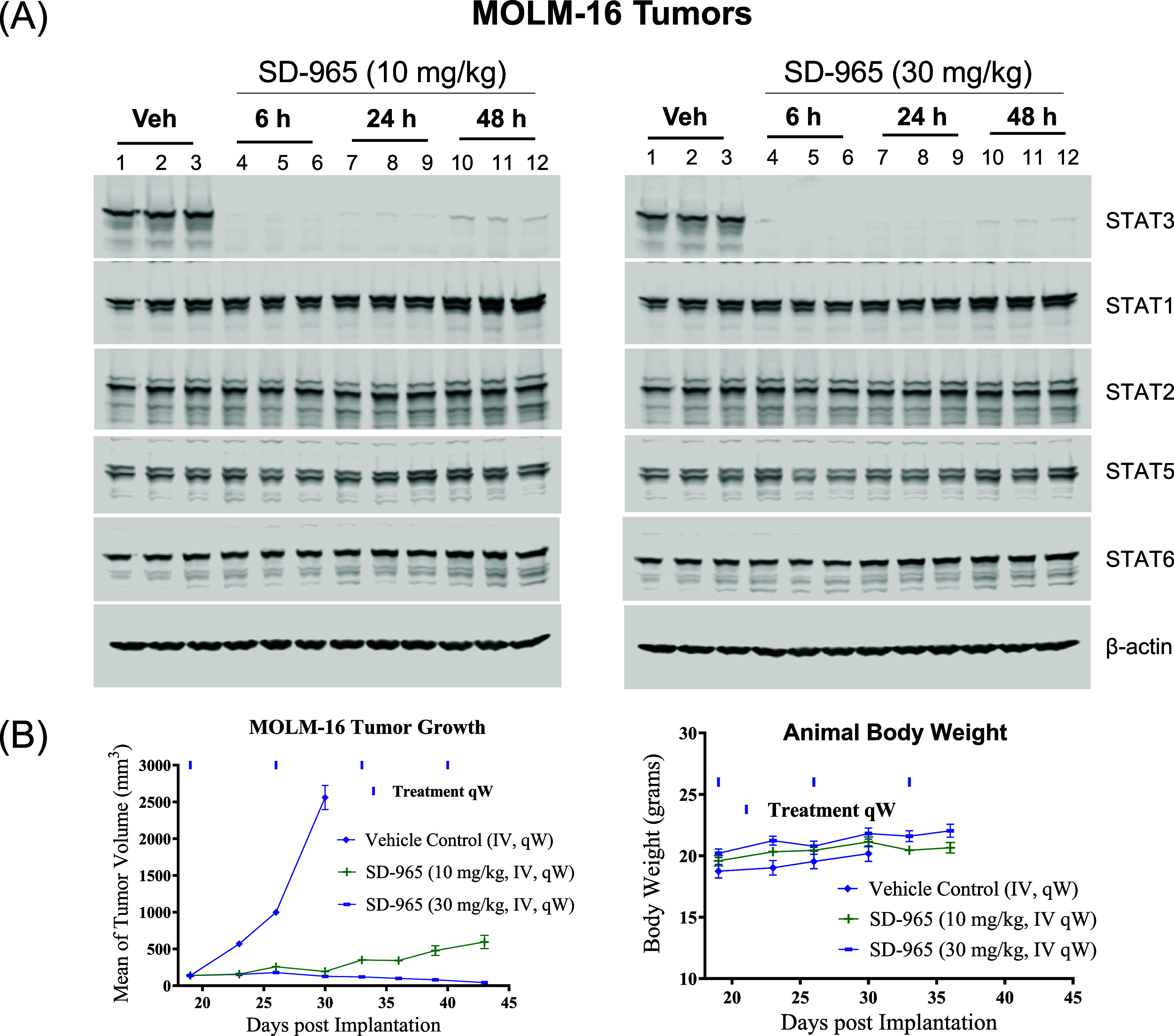
(A) Pharmacodynamics analysis of STAT proteins in the MOLM-16 human acute myeloid leukemia xenograft tumors in SCID mice treated with SD-965 (10 and 30 mg/kg) or vehicle. SCID mice were treated intravenously with a single dose of vehicle or SD-965 at 10 and 30 mg/kg via tail vein injection and were sacrificed for tissue collection at the indicated time points. Tumor lysates were analyzed by immunoblotting of the STAT3 and other STAT proteins. (B) Antitumor activity of SD-965 in the MOLM-16 acute myeloid leukemia xenograft model in SCID mice.

SD-965 was evaluated for its ability to reduce STAT3 protein in the MOLM-16 tumor tissue with a single intravenous administration at 10 and 30 mg/kg. Additionally, we investigated its degradation selectivity on other STAT proteins.

Our PD data (Figure 8A) showed that a single dose of SD-965 at 10 and 30 mg/kg induced near complete (>95%) STAT3 depletion at both 6 and 24 h time points. At the 48 h time point, SD-965 at 10 and 30 mg/kg reduced the STAT3 protein levels by >85 and >95%, respectively (Figure 8A). Hence, a single dose of SD-965 is highly effective in reducing the levels of STAT3 protein in the MOLM-16 tumor tissue.

SD-965 at 10 and 30 mg/kg had no significant effect on the levels of STAT1, STAT2, STAT5, and STAT6 proteins in the MOLM-16 tumor tissue in all of the 3 time points examined. The MOLM-16 cell line has an undetectable level of STAT4 protein. Hence, SD-965 is highly selective in reducing the levels of STAT3 protein over those of other STAT proteins in the MOLM-16 tumor tissue.

Based on the promising PD data, we evaluated SD-965 for its antitumor efficacy in the MOLM-16 tumor model, with the data summarized in Figure 8B. SD-965 dosed at 10 mg/kg weekly intravenously achieved a maximum of 98% tumor growth inhibition. SD-965, dosed at 30 mg/kg weekly intravenously, attained 69% tumor regression after four doses. SD-965 did not induce any weight loss or other signs of toxicity.

### Summary

In this study, we reported our design, synthesis, and evaluation of novel STAT3 PROTAC degraders using a number of high-affinity cereblon ligands and through optimization of the linker and the STAT3 ligands. Our study yielded a number of potent and effective STAT3 degraders with improved STAT3 degradation potencies and cell growth inhibition activity over our previously reported STAT3 degraders, including SD-36, SD-91, and SD-436. Further pharmacodynamic studies in mice identified SD-965 as the most effective STAT3 degrader *in vivo*. SD-965 is potent and effective in inducing degradation of STAT3 protein in cells and displays an excellent degradation selectivity over other STAT proteins. SD-965 is highly selective in reducing the levels of STAT3 protein over other >7000 proteins analyzed in our global proteomics analysis in human PBMCs. *In vivo*, a single intravenous dose of SD-965 is very effective in inducing complete STAT3 depletion in human xenograft tumor tissues and mouse native tissues. Additionally, SD-965 is highly selective in reducing the levels of STAT3 protein over other STAT proteins in the MOLM-16 xenograft tumor tissues. SD-965 was capable of inducing 96% tumor regression in the SU-DHL-1 xenograft tumor model and 69% tumor regression in the MOLM-16 tumor model. Of significance, SD-965 did not induce any weight loss or other signs of toxicity in mice.

Taken together, our data demonstrate that SD-965 is a very promising STAT3 degrader and warrants extensive evaluation as a potential development candidate for the treatment of human cancers and other human diseases.

### Chemistry

The general synthetic route for the compounds listed in Table 1 is summarized in Scheme 1. Our synthesis started with cereblon ligand RR-11055,^40^ which was converted to acids **S2a**–**S2e** through amidation, followed by deprotection under acidic conditions. For the other portion of the degrader molecules, our previously reported intermediate **S3** ^33,34^ was converted to **S4** by deprotecting the Cbz protecting group in the presence of Pd–C under a hydrogen atmosphere. Then, **S5a**–**S5e** was synthesized by an amide coupling reaction followed by the removal of the Boc-protecting groups under acidic conditions, which were subsequently coupled with **S6** ^49^ in the presence of HOBt and diisopropylethylamine (DIPEA) to provide the final compounds **12**–**16**.

**Scheme 1:**
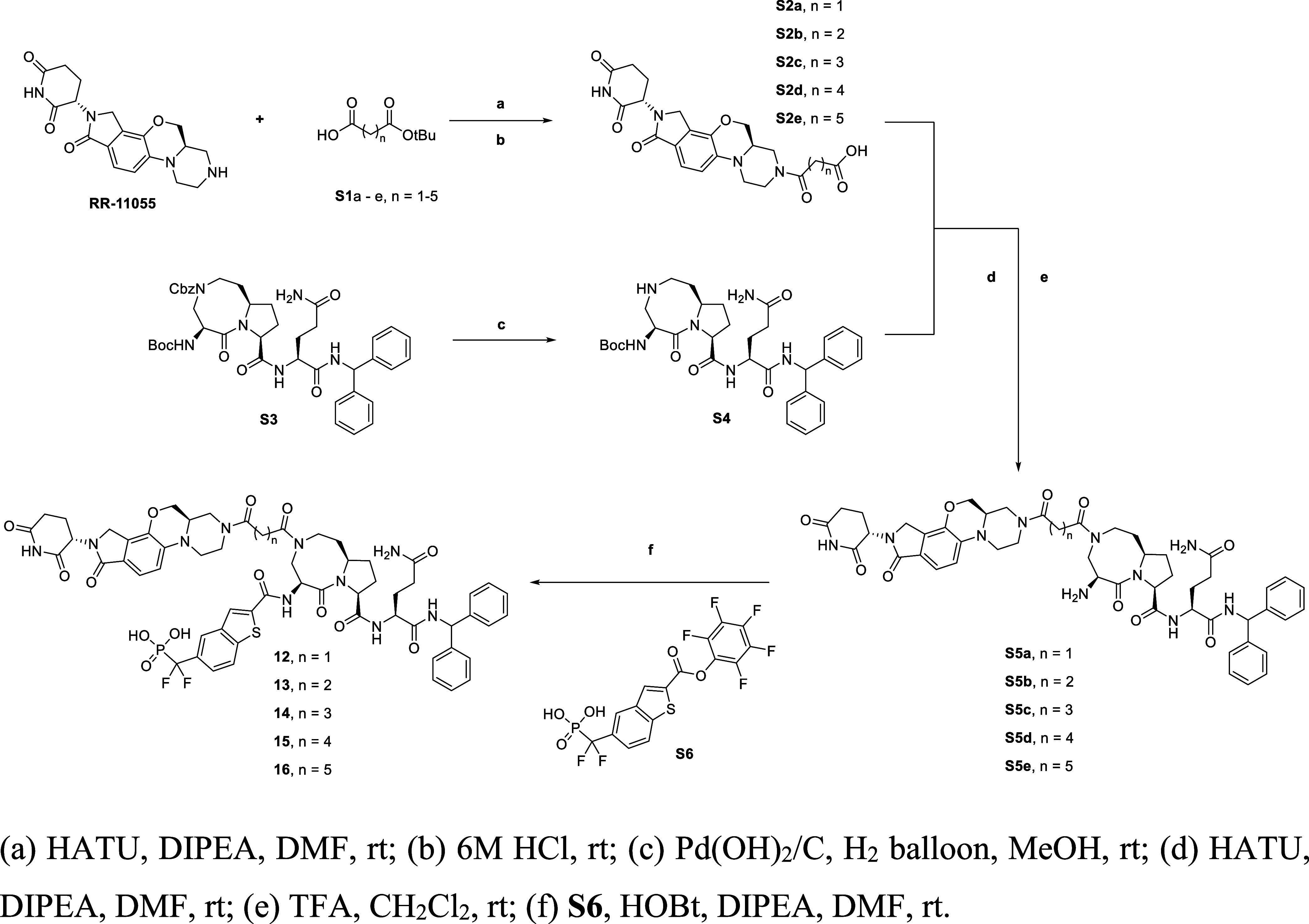
Synthesis of Compounds **12**–**16** Is Presented in Table 1

The general synthetic route for the compounds listed in Table 2 is summarized in Scheme 2. For the amide tail, our synthesis started with an amide coupling reaction to get **S8a**–**S8f**. For the ether tail, a Mitsunobu reaction was used to construct the ether bond to generate intermediate **S8g**–**S8j**. With **S8a**–**S8j** in the hands, we removed their Boc-protecting groups under acidic conditions, then linked them with our previously reported intermediate **S10** by amidation, followed by the removal of the Fmoc-protecting groups under Et_2_NH to get **S12a**–**S12j**. Finally, removed their Boc-protecting groups under acidic conditions, which were subsequently coupled with **S6** ^49^ in the presence of HOBt and DIPEA to provide the final compounds **17**–**26**.

**Scheme 2:**
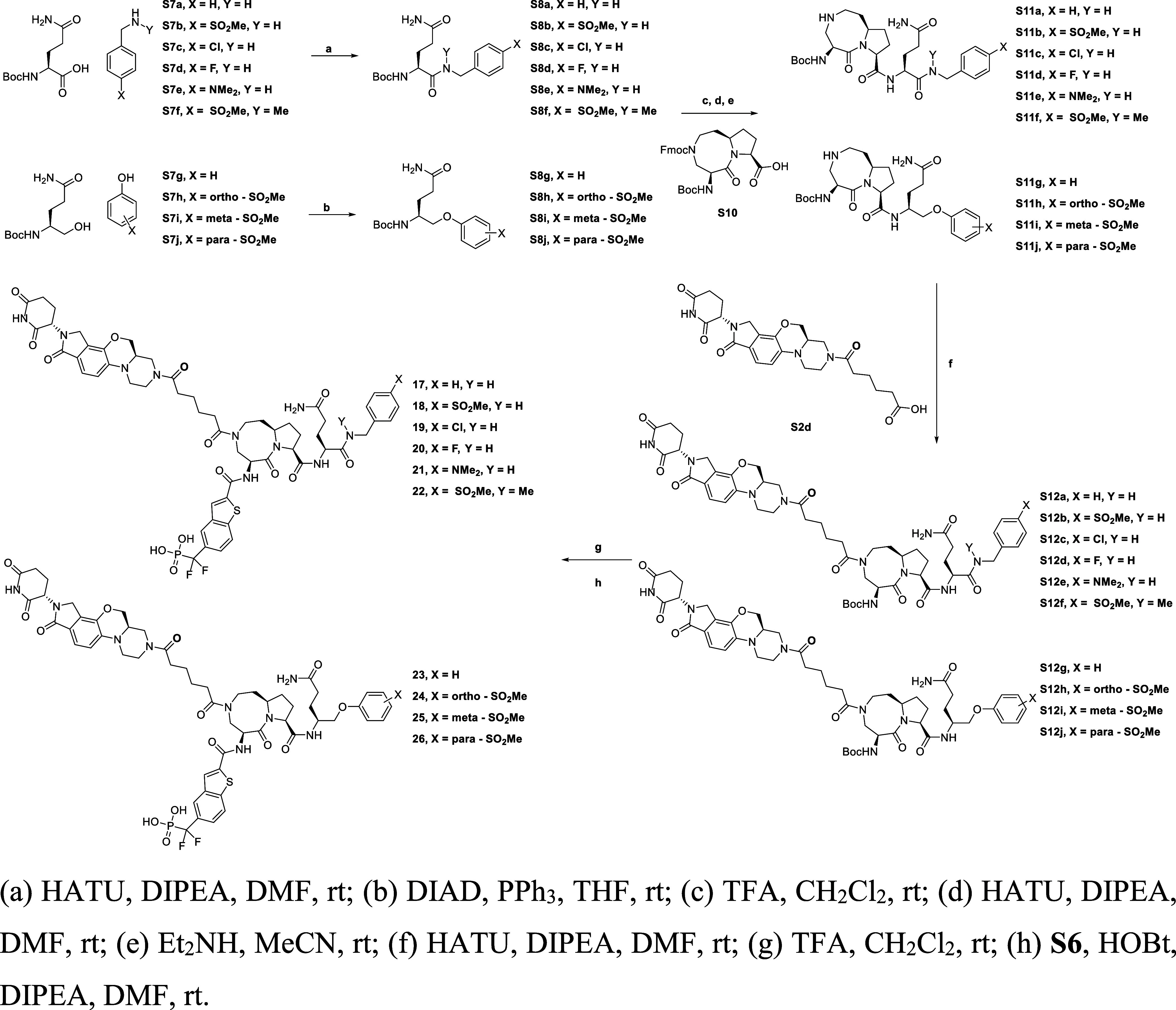
Synthesis of Compounds **17–26** Is Presented in Table 2

The general synthetic route for compound **SD-965** is summarized in Scheme 3. The synthesis started from the same intermediate for making **25**, **S12i**. Removed their Boc-protecting groups under TFA, then crude residue was subsequently coupled with **S13** ^50^ in the presence of HOBt and DIPEA to provide compound **SD-965**.

**Scheme 3:**
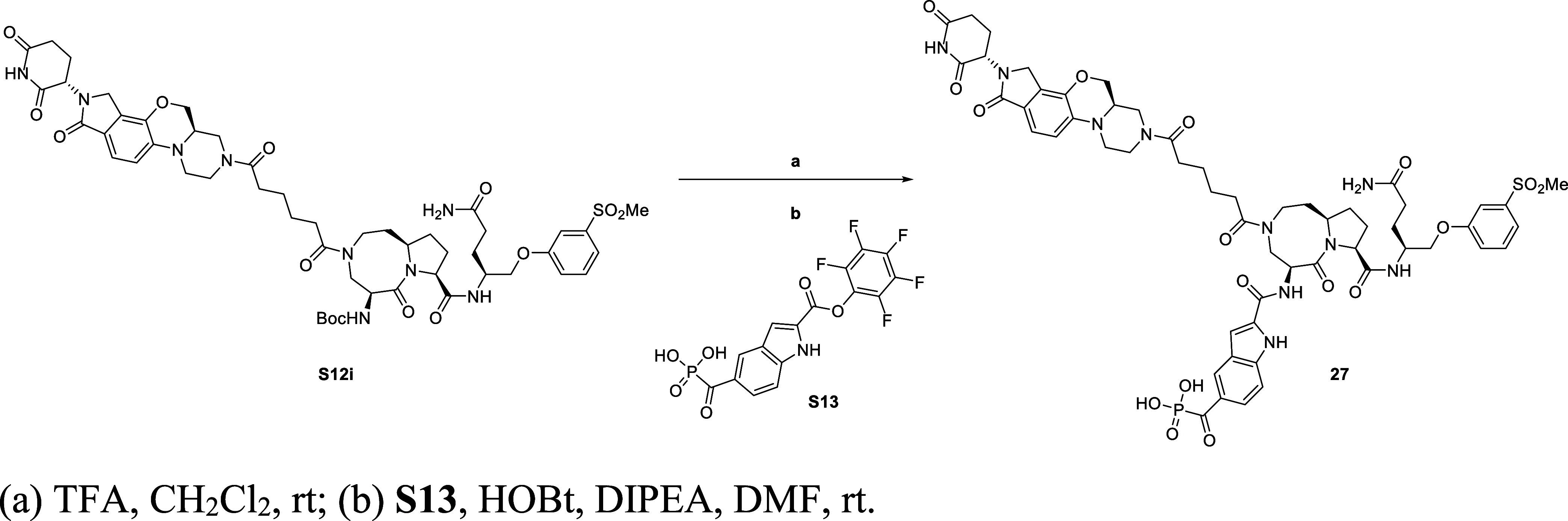
Synthesis of Compounds **SD-965** Is Presented in Table 3

The general synthetic route for the compounds listed in Table 4 is shown in Scheme 4. The synthesis started with reported cereblon ligands **S14a**–**S14m**. **S14a**,^37^ **S14b**,^38^ **S14c**,^41^ and **S14I** ^46^ were synthesized using our previously reported procedures. **S14m** in hydrochloride salt form was purchased from 1PlusChem (San Diego). **S14d**,^51^ **S14e**,^52^ **S14f**,^53^ **S14g**,^53^ **S14h** ^54^ (**54**), **S14i**,^55^ **S14j**,^55^ **S14k** ^54^ were made following those reported methods. **S14a**–**S14k** were converted to acids **S16a**–**S16m** through amidation with **S15**, followed by the removal of *t*-butyl under 6 M HCl. Then, the final compounds **28**–**40** were synthesized through three steps: (1) an amide coupling reaction between **S16a**–**S16m** and **S11i**, (2) followed by the removal of the Boc-protecting groups under acidic conditions, and (3) which were subsequently coupled with **S13** in the presence of HOBt and DIPEA.

**Scheme 4:**
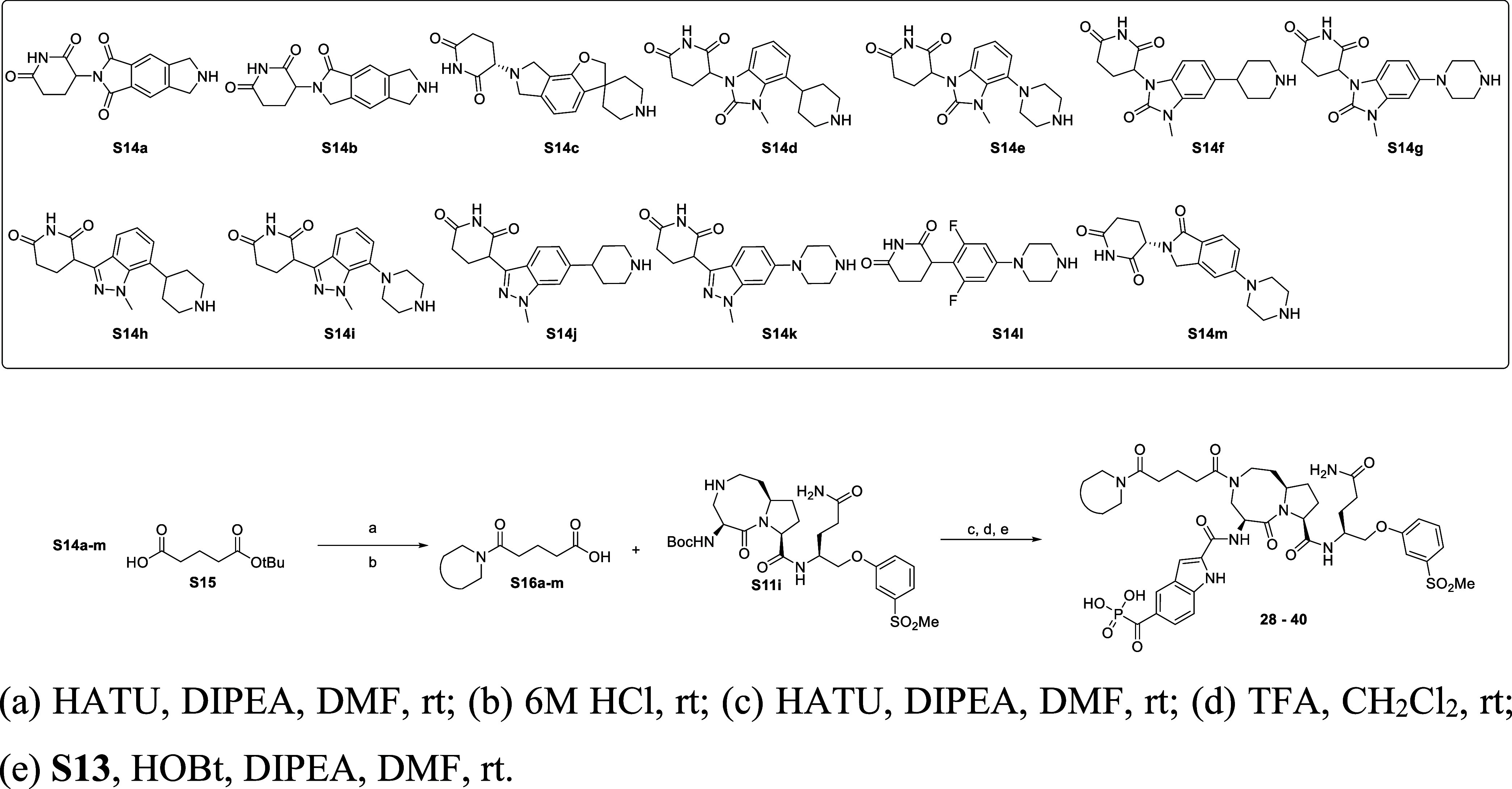
Synthesis of Compounds **28**–**40** Included in Table 4

## Experimental Section

### General Information

All commercial reagents and solvents were used as supplied without further purification, with the following exceptions: tetrahydrofuran (THF) was freshly distilled from sodium wire. The reactions were performed under a N_2_ atmosphere in anhydrous solvents. The final products were purified by reverse phase high-performance liquid chromatography (RP-HPLC) with solvent A (0.1% of TFA in water) and solvent B (0.1% of TFA in CH_3_CN) as eluents with a flow rate of 60 mL/min. The purity of compounds was determined by Waters ACQUITY UPLC, and all of the final compounds were >95% pure. Proton nuclear magnetic resonance (^1^H NMR) and carbon nuclear magnetic resonance (^13^C NMR) spectroscopies were performed on Bruker Advance 400 and 600 NMR spectrometers, and chemical shifts are reported in parts per million (ppm) relative to an internal standard. Mass spectrometry (MS) analysis was carried out with a Thermo-Scientific LCQ Fleet mass spectrometer or a Waters ultraperformance liquid chromatography (UPLC)-mass spectrometer.

#### *tert*-Butyl ((5*S*,8*S*,10*aR*)-8-(((*S*)-5-Amino-1-(benzhydrylamino)-1,5-dioxopentan-2-yl)­carbamoyl)-6-oxodecahydropyrrolo­[1,2-*a*]­[1,5]­diazocin-5-yl)­carbamate (**S4**)

To a stirred solution of compound **S3** (500 mg, 0.66 mmol, 1 equiv) in methanol (10 mL) at room temperature, Pd­(OH)_2_/C (50 mg) was added, air was removed, and filled with H_2_ three times. The reaction mixture was stirred for another 4 h under a hydrogen environment. After the complete disappearance of the starting material, the reaction was filtered through a pad of Celite and washed with methanol. The filtrate was concentrated under reduced pressure to furnish compound **S4** as a white solid. UPLC-MS (ESI) *m*/*z*: calcd, 621.33 for C_33_H_44_N_6_O_6_ [M + H]^+^; found, 621.37. ^1^H NMR (400 MHz, methanol-*d*_4_) δ 7.42–7.22 (m, 10H), 6.18 (s, 1H), 4.99 (dd, *J* = 11.9, 5.9 Hz, 1H), 4.68 (t, *J* = 9.1 Hz, 1H), 4.59–4.43 (m, 2H), 3.54 (dt, *J* = 14.5, 3.9 Hz, 1H), 3.25 (td, *J* = 13.1, 8.1 Hz, 2H), 2.82 (t, *J* = 12.1 Hz, 1H), 2.50–2.27 (m, 3H), 2.22 (dtd, *J* = 16.3, 8.2, 4.7 Hz, 2H), 2.06 (h, *J* = 8.7 Hz, 1H), 1.93 (dtd, *J* = 10.1, 7.9, 3.7 Hz, 2H), 1.81 (ddd, *J* = 26.5, 13.7, 5.0 Hz, 2H), 1.47 (s, 9H).

### General Procedure for the Synthesis of Compounds **12**–**16**

**S1a**–**S1e** (0.1 mol, 1 equiv) was dissolved in 1 mL of DMF, DIPEA (0.053 mL, 0.3 mol, 3 equiv) and HATU (33.8 mg, 0.1 mol, 1 equiv) were added, and the mixture was stirred for 10 min, then RR-11055 was added, and the mixture was stirred for 30 min. The products were purified using preparative HPLC using acetonitrile/H_2_O (0.1% TFA). 6 M HCl was used to remove *tert*-butyl to get the desired intermediates **S2a**–**S2e**. **S2a**–**S2e** (1 equiv) was dissolved in DMF, DIPEA (3 equiv) and HATU (1 equiv) were added, and the mixture was stirred for 10 min, then **S4** was added, and the mixture was stirred for 30 min. The products were purified using preparative HPLC using acetonitrile: H_2_O (0.1% TFA). TFA/dichloromethane (DCM) = 1:1 was used to remove Boc to get the desired intermediates **S5a**–**S5e**. To a stirred solution of crude amine (1 equiv), compound **S6** (1.5 equiv), and HOBt (1.5 equiv) in DMF, DIPEA (5 equiv) was added at room temperature. The reaction mixture was then stirred at room temperature for an hour. After the complete disappearance of the starting amine, the product was purified using a preparative HPLC to provide compounds **12**–**16** as a white solid.

#### ((2-(((5*S*,8*S*,10*aR*)-8-(((*S*)-5-Amino-1-(benzhydrylamino)-1,5-dioxopentan-2-yl)­carbamoyl)-3-(3-((*R*)-2-((*S*)-2,6-dioxopiperidin-3-yl)-1-oxo-2,3,5*a*,6,8,9-hexahydro-1*H*-pyrazino­[1′,2′:4,5]­[1,4]­oxazino­[2,3-*e*]­isoindol-7­(5*H*)-yl)-3-oxopropanoyl)-6-oxodecahydropyrrolo­[1,2-*a*]­[1,5]­diazocin-5-yl)­carbamoyl)­benzo­[*b*]­thiophen-5-yl)­difluoromethyl)­phosphonic Acid (**12**)

UPLC-MS (ESI) *m*/*z*: calcd, 618.20 for C_59_H_61_F_2_N_10_O_14_PS [M + 2H]^+^/2; found, 617.93. ^1^H NMR (400 MHz, DMSO-*d*_6_/D_2_O) δ 8.30–8.19 (m, 1H), 8.17–7.97 (m, 2H), 7.62–7.44 (m, 1H), 7.39–7.15 (m, 11H), 7.12–6.85 (m, 1H), 6.20–5.94 (m, 1H), 5.10–4.88 (m, 2H), 4.52–4.20 (m, 6H), 4.19–3.79 (m, 6H), 3.39–3.11 (m, 3H), 3.06–2.76 (m, 2H), 2.70–2.53 (m, 3H), 2.44–2.30 (m, 2H), 2.27–1.50 (m, 12H).

#### ((2-(((5*S*,8*S*,10*aR*)-8-(((*S*)-5-Amino-1-(benzhydrylamino)-1,5-dioxopentan-2-yl)­carbamoyl)-3-(4-((*R*)-2-((*S*)-2,6-dioxopiperidin-3-yl)-1-oxo-2,3,5*a*,6,8,9-hexahydro-1*H*-pyrazino­[1′,2′:4,5]­[1,4]­oxazino­[2,3-*e*]­isoindol-7­(5*H*)-yl)-4-oxobutanoyl)-6-oxodecahydropyrrolo­[1,2-*a*]­[1,5]­diazocin-5-yl)­carbamoyl)­benzo­[*b*]­thiophen-5-yl)­difluoromethyl)­phosphonic Acid (**13**)

UPLC-MS (ESI) *m*/*z*: calcd, 625.21 for C_60_H_63_F_2_N_10_O_14_PS [M + 2H]^+^/2; found, 625.18. ^1^H NMR (400 MHz, DMSO-*d*_6_/D_2_O) δ 8.31–8.20 (m, 1H), 8.17–8.06 (m, 2H), 7.62–7.55 (m, 1H), 7.38–7.15 (m, 11H), 7.14–6.98 (m, 1H), 6.07 (s, 1H), 5.13–4.94 (m, 2H), 4.51–4.20 (m, 6H), 4.17–3.84 (m, 6H), 3.48–3.34 (m, 1H), 3.33–3.06 (m, 1H), 3.00–2.72 (m, 4H), 2.71–2.53 (m, 4H), 2.47–2.24 (m, 2H), 2.24–1.53 (m, 12H).

#### ((2-(((5*S*,8*S*,10*aR*)-8-(((*S*)-5-Amino-1-(benzhydrylamino)-1,5-dioxopentan-2-yl)­carbamoyl)-3-(5-((*R*)-2-((*S*)-2,6-dioxopiperidin-3-yl)-1-oxo-2,3,5*a*,6,8,9-hexahydro-1*H*-pyrazino­[1′,2′:4,5]­[1,4]­oxazino­[2,3-*e*]­isoindol-7­(5*H*)-yl)-5-oxopentanoyl)-6-oxodecahydropyrrolo­[1,2-*a*]­[1,5]­diazocin-5-yl)­carbamoyl)­benzo­[*b*]­thiophen-5-yl)­difluoromethyl)­phosphonic Acid (**14**)

UPLC-MS (ESI) *m*/*z*: calcd, 632.22 for C_61_H_65_F_2_N_10_O_14_PS [M + 2H]^+^/2; 632.13. found, ^1^H NMR (400 MHz, DMSO-*d*_6_/D_2_O) δ 8.32–8.24 (m, 1H), 8.11 (q, *J* = 8.9 Hz, 2H), 7.62–7.56 (m, 1H), 7.35–7.16 (m, 11H), 7.09–6.97 (m, 1H), 6.11–6.04 (m, 1H), 5.06–4.91 (m, 2H), 4.50–4.18 (m, 6H), 4.16–3.78 (m, 6H), 3.47–3.31 (m, 2H), 3.29–3.17 (m, 1H), 3.15–3.02 (m, 1H), 2.96–2.69 (m, 3H), 2.69–2.53 (m, 2H), 2.47–2.27 (m, 5H), 2.24–1.55 (m, 12H).

#### ((2-(((5*S*,8*S*,10*aR*)-8-(((*S*)-5-Amino-1-(benzhydrylamino)-1,5-dioxopentan-2-yl)­carbamoyl)-3-(6-((*R*)-2-((*S*)-2,6-dioxopiperidin-3-yl)-1-oxo-2,3,5*a*,6,8,9-hexahydro-1*H*-pyrazino­[1′,2′:4,5]­[1,4]­oxazino­[2,3-*e*]­isoindol-7­(5*H*)-yl)-6-oxohexanoyl)-6-oxodecahydropyrrolo­[1,2-*a*]­[1,5]­diazocin-5-yl)­carbamoyl)­benzo­[*b*]­thiophen-5-yl)­difluoromethyl)­phosphonic Acid (**15**)

UPLC-MS (ESI) *m*/*z*: calcd, 639.22 for C_62_H_67_F_2_N_10_O_14_PS [M + 2H]^+^/2; found 639.03. ^1^H NMR (400 MHz, DMSO-*d*_6_/D_2_O) δ 8.34–8.21 (m, 1H), 8.17–8.00 (m, 2H), 7.62–7.55 (m, 1H), 7.41–7.13 (m, 11H), 7.08–6.92 (m, 1H), 6.13–5.96 (m, 1H), 5.07–4.70 (m, 2H), 4.51–4.30 (m, 4H), 4.30–4.15 (m, 3H), 4.15–3.92 (m, 5H), 3.47–3.28 (m, 2H), 3.28–3.13 (m, 1H), 3.11–2.98 (m, 1H), 2.95–2.68 (m, 3H), 2.65–2.52 (m, 2H), 2.48–2.25 (m, 5H), 2.24–2.05 (m, 3H), 2.04–1.41 (m, 11H).

#### ((2-(((5*S*,8*S*,10*aR*)-8-(((*S*)-5-Amino-1-(benzhydrylamino)-1,5-dioxopentan-2-yl)­carbamoyl)-3-(7-((*R*)-2-((*S*)-2,6-dioxopiperidin-3-yl)-1-oxo-2,3,5*a*,6,8,9-hexahydro-1*H*-pyrazino­[1′,2′:4,5]­[1,4]­oxazino­[2,3-*e*]­isoindol-7­(5*H*)-yl)-7-oxoheptanoyl)-6-oxodecahydropyrrolo­[1,2-*a*]­[1,5]­diazocin-5-yl)­carbamoyl)­benzo­[*b*]­thiophen-5-yl)­difluoromethyl)­phosphonic Acid (**16**)

UPLC-MS (ESI) *m*/*z*: calcd, 646.23 for C_63_H_69_F_2_N_10_O_14_PS [M + 2H]^+^/2; found 646.10. ^1^H NMR (400 MHz, DMSO-*d*_6_/D_2_O) δ 8.36–8.21 (m, 1H), 8.16–8.01 (m, 2H), 7.66–7.52 (m, 1H), 7.39–7.11 (m, 11H), 7.09–6.95 (m, 1H), 6.15–5.98 (m, 1H), 5.07–4.69 (m, 2H), 4.52–4.30 (m, 4H), 4.30–4.15 (m, 2H), 4.15–4.05 (m, 1H), 4.04–3.81 (m, 5H), 3.48–3.29 (m, 2H), 3.28–3.13 (m, 1H), 3.12–2.99 (m, 1H), 2.96–2.80 (m, 2H), 2.80–2.68 (m, 1H), 2.68–2.52 (m, 2H), 2.47–2.25 (m, 5H), 2.24–2.04 (m, 3H), 2.03–1.44 (m, 11H), 1.40–1.25 (m, 2H).

#### *tert*-Butyl (*S*)-(5-Amino-1-(benzylamino)-1,5-dioxopentan-2-yl)­carbamate (**S8a**)

To a stirred solution of Boc-l-glutamine (246 mg, 1 mmol, 1equiv) in 5 mL DMF, DIPEA (0.52 mL, 3 mmol, 3 equiv) and HATU (380 mg, 1 mmol, 1 equiv) were added, and the mixture was stirred for 30 min. Then, benzylamine (128 mg, 1.2 mmol, 1.2 equiv) was added, and the mixture was stirred for 1 h. The product was purified using preparative HPLC with acetonitrile/H_2_O to get **S8a** as a white solid. UPLC-MS (ESI) *m*/*z*: calcd, 335.18 for C_17_H_25_N_3_O_4_ [M + H]^+^; found, 336.39. ^1^H NMR (400 MHz, DMSO-*d*_6_) δ 8.30 (t, *J* = 6.0 Hz, 1H), 7.45–7.14 (m, 6H), 6.93 (d, *J* = 7.9 Hz, 1H), 6.77 (s, 1H), 4.37–4.18 (m, 2H), 4.00–3.84 (m, 1H), 2.24–1.99 (m, 2H), 1.93–1.79 (m, 1H), 1.78–1.63 (m, 1H), 1.39 (s, 9H).

#### *tert*-Butyl (*S*)-(5-Amino-1-((4-(methylsulfonyl)­benzyl)­amino)-1,5-dioxopentan-2-yl)­carbamate (**S8b**)

This intermediate was synthesized by the same procedure as that of **S8a** as a white solid. UPLC-MS (ESI) *m*/*z*: calcd, 413.16 for C_18_H_27_N_3_O_6_S [M + Na]^+^; found, 436.29. ^1^H NMR (400 MHz, DMSO-*d*_6_) 8.48 (t, *J* = 6.1 Hz, 1H), 7.84 (d, *J* = 8.3 Hz, 2H), 7.50 (d, *J* = 8.2 Hz, 2H), 7.28 (s, 1H), 7.02 (d, *J* = 7.6 Hz, 1H), 6.78 (s, 1H), 4.38 (d, *J* = 6.0 Hz, 2H), 3.97–3.84 (m, 1H), 3.19 (s, 3H), 2.21–2.03 (m, 2H), 1.93–1.80 (m, 1H), 1.79–1.65 (m, 1H), 1.39 (s, 9H).

#### *tert*-Butyl (*S*)-(5-Amino-1-((4-chlorobenzyl)­amino)-1,5-dioxopentan-2-yl)­carbamate (**S8c**)

This intermediate was synthesized by the same procedure as that of **S8a** as a white solid. UPLC-MS (ESI) *m*/*z*: calcd, 369.15 for C_17_H_24_ClN_3_O_4_ [M + Na]^+^; found, 392.34. ^1^H NMR (400 MHz, DMSO-*d*_6_) ^1^H NMR (400 MHz, DMSO) δ 8.35 (t, *J* = 6.0 Hz, 1H), 7.41–7.31 (m, 2H), 7.30–7.18 (m, 3H), 6.96 (d, *J* = 7.8 Hz, 1H), 6.76 (s, 1H), 4.26 (d, *J* = 6.0 Hz, 2H), 3.94–3.82 (m, 1H), 2.20–1.99 (m, 2H), 1.91–1.76 (m, 1H), 1.76–1.62 (m, 1H), 1.38 (s, 9H).

#### *tert*-Butyl (*S*)-(5-Amino-1-((4-fluorobenzyl)­amino)-1,5-dioxopentan-2-yl)­carbamate (**S8d**)

This intermediate was synthesized by the same procedure as that of **S8a** as a white solid. UPLC-MS (ESI) *m*/*z*: calcd, 353.18 for C_17_H_24_FN_3_O_4_ [M + H]^+^; found, 354.36. ^1^H NMR (400 MHz, DMSO-*d*_6_) δ 8.33 (t, *J* = 6.0 Hz, 1H), 7.36–7.19 (m, 3H), 7.18–7.04 (m, 2H), 6.94 (d, *J* = 7.9 Hz, 1H), 6.77 (s, 1H), 4.25 (d, *J* = 6.0 Hz, 2H), 3.94–3.84 (m, 1H), 2.18–2.00 (m, 2H), 1.92–1.76 (m, 1H), 1.76–1.63 (m, 1H), 1.38 (s, 9H).

#### *tert*-Butyl (*S*)-(5-Amino-1-((4-(dimethylamino)­benzyl)­amino)-1,5-dioxopentan-2-yl)­carbamate (**S8e**)

This intermediate was synthesized by the same procedure as that of **S8a** as a white solid. UPLC-MS (ESI) *m*/*z*: calcd, 378.23 for C_19_H_30_N_4_O_4_ [M + H]^+^; found, 379.48. ^1^H NMR (400 MHz, DMSO-*d*_6_) δ 8.14–8.03 (m, 1H), 7.24 (s, 1H), 7.12–6.97 (m, 2H), 6.91–6.78 (m, 1H), 6.74 (s, 1H), 6.69–6.63 (m, 2H), 4.25–4.04 (m, 2H), 3.93–3.83 (m, 1H), 2.85 (s, 6H), 2.21–1.95 (m, 2H), 1.89–1.75 (m, 1H), 1.74–1.60 (m, 1H), 1.38 (s, 9H).

#### *tert*-Butyl (*S*)-(5-Amino-1-(methyl­(4-(methylsulfonyl)­benzyl)­amino)-1,5-dioxopentan-2-yl)­carbamate (**S8f**)

This intermediate was synthesized by the same procedure as that of **S8a** as a white solid. UPLC-MS (ESI) *m*/*z*: calcd, 427.18 for C_19_H_29_N_3_O_6_S [M + Na]^+^; found, 450.33. ^1^H NMR (400 MHz, DMSO-*d*_6_) δ 7.97–7.80 (m, 2H), 7.63–7.40 (m, 2H), 7.28 (d, *J* = 17.0 Hz, 1H), 7.13–7.00 (m, 1H), 6.85–6.68 (m, 1H), 5.18–4.64 (m, 1H), 4.63–4.48 (m, 1H), 4.46–4.28 (m, 1H), 3.24–3.16 (m, 3H), 3.10–2.69 (m, 3H), 2.27–2.03 (m, 2H), 1.92–1.78 (m, 1H), 1.73–1.57 (m, 1H), 1.45–1.27 (m, 9H).

#### *tert*-Butyl (*S*)-(5-Amino-5-oxo-1-phenoxypentan-2-yl)­carbamate (**S8g**)

To a stirred solution of *tert*-butyl (*S*)-(5-amino-1-hydroxy-5-oxopentan-2-yl)­carbamate (464 mg, 2 mmol, 1 equiv), phenol (188 mg, 2 mmol, 1 equiv), and PPh_3_ (1.1 g, 4.2 mmol, 2.1 equiv) in 5 mL of THF, DIAD (0.79 mL, 4 mmol, 2 equiv) was dropped into the flask. The mixture was stirred for 2 h. The solvent was removed under vacuum, and the crude residue was purified using preparative HPLC with acetonitrile/H_2_O to get **S8g** as a white solid. UPLC-MS (ESI) *m*/*z*: calcd, 308.17 for C_16_H_24_N_2_O_4_ [M + Na]^+^; found, 331.35. ^1^H NMR (400 MHz, DMSO-*d*_6_) δ 7.34–7.22 (m, 3H), 6.95–6.89 (m, 3H), 6.84 (d, *J* = 8.5 Hz, 1H), 6.74 (s, 1H), 3.96–3.78 (m, 2H), 3.77–3.66 (m, 1H), 2.23–1.98 (m, 2H), 1.89–1.73 (m, 1H), 1.66–1.51 (m, 1H), 1.38 (s, 9H).

#### *tert*-Butyl (*S*)-(5-Amino-1-(2-(methylsulfonyl)­phenoxy)-5-oxopentan-2-yl)­carbamate (**S8h**)

This intermediate was synthesized by the same procedure as that of **S8g** as a white solid. UPLC-MS (ESI) *m*/*z*: calcd, 386.15 for C_17_H_26_N_2_O_6_S [M + Na]^+^; found, 409.32. ^1^H NMR (400 MHz, DMSO-*d*_6_) 7.80 (dd, *J* = 7.8, 1.7 Hz, 1H), 7.72–7.63 (m, 1H), 7.35–7.20 (m, 2H), 7.19–7.10 (m, 1H), 6.87 (d, *J* = 8.5 Hz, 1H), 6.76 (s, 1H), 4.15–4.00 (m, 2H), 3.90–3.77 (m, 1H), 3.25 (s, 3H), 2.20–2.08 (m, 2H), 1.91–1.79 (m, 1H), 1.70–1.57 (m, 1H), 1.37 (s, 9H).

#### *tert*-Butyl (*S*)-(5-Amino-1-(3-(methylsulfonyl)­phenoxy)-5-oxopentan-2-yl)­carbamate (**S8i**)

This intermediate was synthesized by the same procedure as that of **S8g** as a white solid. UPLC-MS (ESI) *m*/*z*: calcd, 386.15 for C_17_H_26_N_2_O_6_S [M + Na]^+^; found, 409.30. ^1^H NMR (400 MHz, DMSO-*d*_6_) δ 7.59–7.52 (m, 1H), 7.51–7.46 (m, 1H), 7.42 (s, 1H), 7.32–7.23 (m, 2H), 6.89 (d, *J* = 8.5 Hz, 1H), 6.74 (s, 1H), 4.06–3.91 (m, 2H), 3.80–3.70 (m, 1H), 3.22 (s, 3H), 2.21–2.04 (m, 2H), 1.89–1.76 (m, 1H), 1.71–1.57 (m, 1H), 1.38 (s, 9H).

#### *tert*-Butyl (*S*)-(5-Amino-1-(4-(methylsulfonyl)­phenoxy)-5-oxopentan-2-yl)­carbamate (**S8j**)

This intermediate was synthesized by the same procedure as that of **S8g** as a white solid. UPLC-MS (ESI) *m*/*z*: calcd, 386.15 for C_17_H_26_N_2_O_6_S [M + Na]^+^; found, 409.32. ^1^H NMR (400 MHz, DMSO-*d*_6_) δ 7.91–7.78 (m, 2H), 7.27 (s, 1H), 7.18–7.07 (m, 2H), 6.88 (d, *J* = 8.5 Hz, 1H), 6.79–6.66 (m, 1H), 4.03–3.93 (m, 2H), 3.82–3.68 (m, 1H), 3.14 (s, 3H), 2.23–2.04 (m, 2H), 1.86–1.74 (m, 1H), 1.72–1.53 (m, 1H), 1.38 (s, 9H).

#### *tert*-Butyl ((5*S*,8*S*,10*aR*)-8-(((*S*)-5-Amino-1-(benzylamino)-1,5-dioxopentan-2-yl)­carbamoyl)-6-oxodecahydropyrrolo­[1,2-*a*]­[1,5]­diazocin-5-yl)­carbamate (**S11a**)

**S8a** (111 mg, 0.33 mmol, 1 equiv) was dissolved in 2.5 mL of DCM, and 2.5 mL of TFA was added. The mixture was stirred for 30 min, then the solvent was removed under vacuum to get crude residue **S9a** without further purification. To a stirred solution of **S10** (145 mg, 0.26 mmol, 0.8 equiv) in 2 mL of DMF, DIPEA (0.58 mL, 3.3 mmol, 10 equiv) and HATU (99 mg, 0.26 mmol, 0.8 equiv) were added, and the mixture was stirred for 30 min. Then, the crude residue **S9a** was added. The mixture was stirred for 1 h, quenched with NaHCO_3_ aqueous solution, extracted with EtOAc (5 mL × 5), washed with brine three times, and dried with anhydrous sodium sulfate. Then, it was filtered and the solvent was removed under vacuum. The residual crude product was dissolved in 2 mL of MeCN, and 1 mL of Et_2_NH was added; the mixture was stirred for 2 h. The solvent was removed under vacuum, and the crude residue was purified using preparative HPLC with acetonitrile/H_2_O (0.1% HCOOH) to get **S11a** as a white solid. UPLC-MS (ESI) *m*/*z*: calcd, 544.30 for C_27_H_40_N_6_O_6_ [M + Na]^+^; found, 545.35. ^1^H NMR (400 MHz, DMSO-*d*_6_) δ 8.91–8.82 (m, 1H), 8.53–8.41 (m, 1H), 7.36–7.18 (m, 7H), 6.87–6.69 (m, 1H), 4.98–4.81 (m, 1H), 4.69–4.57 (m, 1H), 4.53–4.42 (m, 1H), 4.37–4.18 (m, 3H), 3.35–3.24 (m, 1H), 3.05–2.94 (m, 1H), 2.58–2.52 (m, 2H), 2.41–2.28 (m, 1H), 2.21–1.65 (m, 10H), 1.38 (s, 9H).

#### *tert*-Butyl ((5*S*,8*S*,10*aR*)-8-(((*S*)-5-Amino-1-((4-(methylsulfonyl)­benzyl)­amino)-1,5-dioxopentan-2-yl)­carbamoyl)-6-oxodecahydropyrrolo­[1,2-*a*]­[1,5]­diazocin-5-yl)­carbamate (**S11b**)

This intermediate was synthesized by the same procedure as that of **S11a** as a white solid. UPLC-MS (ESI) *m*/*z*: calcd, 622.28 for C_28_H_42_N_6_O_8_S [M + H]^+^; found, 623.04. ^1^H NMR (400 MHz, DMSO-*d*_6_) δ 8.94–8.82 (m, 1H), 8.64–8.57 (m, 1H), 7.91–7.84 (m, 2H), 7.52–7.46 (m, 3H), 7.31–7.26 (m, 1H), 6.81 (s, 1H), 4.99–4.84 (m, 1H), 4.67–4.58 (m, 1H), 4.54–4.31 (m, 3H), 4.29–4.19 (m, 1H), 3.19 (s, 3H), 3.05–2.86 (m, 1H), 2.70–2.53 (m, 3H), 2.40–2.25 (m, 1H), 2.23–2.00 (m, 4H), 1.99–1.87 (m, 1H), 1.86–1.68 (m, 5H), 1.38 (s, 9H).

#### *tert*-Butyl ((5*S*,8*S*,10*aR*)-8-(((*S*)-5-Amino-1-((4-chlorobenzyl)­amino)-1,5-dioxopentan-2-yl)­carbamoyl)-6-oxodecahydropyrrolo­[1,2-*a*]­[1,5]­diazocin-5-yl)­carbamate (**S11c**)

This intermediate was synthesized by the same procedure as that of **S11a** as a white solid. UPLC-MS (ESI) *m*/*z*: calcd, 578.26 for C_27_H_39_ClN_6_O_6_ [M + H]^+^; found, 578.93. ^1^H NMR (400 MHz, DMSO-*d*_6_) δ 9.11–8.85 (m, 1H), 8.06–7.82 (m, 1H), 7.40–7.33 (m, 2H), 7.32–7.21 (m, 3H), 6.92–6.74 (m, 2H), 4.78–4.56 (m, 1H), 4.48–4.20 (m, 4H), 4.18–4.05 (m, 1H), 3.23–3.08 (m, 1H), 2.87–2.77 (m, 1H), 2.75–2.56 (m, 2H), 2.40–2.25 (m, 1H), 2.15–1.96 (m, 4H), 1.94–1.63 (m, 5H), 1.55–1.42 (m, 1H), 1.37 (s, 9H).

#### *tert*-Butyl ((5*S*,8*S*,10*aR*)-8-(((*S*)-5-Amino-1-((4-fluorobenzyl)­amino)-1,5-dioxopentan-2-yl)­carbamoyl)-6-oxodecahydropyrrolo­[1,2-*a*]­[1,5]­diazocin-5-yl)­carbamate (**S11d**)

This intermediate was synthesized by the same procedure as that of **S11a** as a white solid. UPLC-MS (ESI) *m*/*z*: calcd, 562.29 for C_27_H_39_FN_6_O_6_ [M + H]^+^; found, 563.33. ^1^H NMR (400 MHz, DMSO-*d*_6_) δ 9.03–8.80 (m, 1H), 8.56–8.45 (m, 1H), 7.32–7.21 (m, 4H), 7.18–7.11 (m, 2H), 6.80 (s, 1H), 5.02–4.76 (m, 1H), 4.70–4.56 (m, 1H), 4.55–4.40 (m, 1H), 4.36–4.10 (m, 3H), 3.34–3.19 (m, 2H), 3.07–2.91 (m, 1H), 2.40–2.23 (m, 1H), 2.10 (tt, *J* = 16.5, 6.2 Hz, 4H), 1.98–1.65 (m, 7H), 1.38 (s, 9H).

#### *tert*-Butyl ((5*S*,8*S*,10*aR*)-8-(((*S*)-5-Amino-1-((4-(dimethylamino)­benzyl)­amino)-1,5-dioxopentan-2-yl)­carbamoyl)-6-oxodecahydropyrrolo­[1,2-*a*]­[1,5]­diazocin-5-yl)­carbamate (**S11e**)

This intermediate was synthesized by the same procedure as that of **S11a** as a white solid. UPLC-MS (ESI) *m*/*z*: calcd, 587.34 for C_29_H_45_N_7_O_6_ [M + H]^+^; found, 588.41. ^1^H NMR (400 MHz, DMSO-*d*_6_) δ 8.87–8.79 (m, 1H), 8.36–8.19 (m, 1H), 7.29 (d, *J* = 7.3 Hz, 1H), 7.22 (s, 1H), 7.06 (d, *J* = 8.4 Hz, 2H), 6.79 (s, 1H), 6.68 (d, *J* = 8.3 Hz, 2H), 4.98–4.82 (m, 1H), 4.70–4.56 (m, 1H), 4.53–4.37 (m, 1H), 4.28–4.15 (m, 2H), 4.13–4.02 (m, 1H), 3.31–3.22 (m, 1H), 3.06–2.93 (m, 1H), 2.86 (s, 6H), 2.40–2.26 (m, 2H), 2.20–2.01 (m, 3H), 1.99–1.67 (m, 8H), 1.38 (s, 9H).

#### *tert*-Butyl ((5*S*,8*S*,10*aR*)-8-(((*S*)-5-Amino-1-(methyl­(4-(methylsulfonyl)­benzyl)­amino)-1,5-dioxopentan-2-yl)­carbamoyl)-6-oxodecahydropyrrolo­[1,2-*a*]­[1,5]­diazocin-5-yl)­carbamate (**S11f**)

This intermediate was synthesized by the same procedure as that of **S11a** as a white solid. UPLC-MS (ESI) *m*/*z*: calcd, 636.29 for C_29_H_44_N_6_O_8_S [M + H]^+^; found, 637.08. ^1^H NMR (400 MHz, DMSO-*d*_6_) δ 9.01–8.84 (m, 1H), 7.97–7.82 (m, 2H), 7.54–7.37 (m, 2H), 7.30–7.04 (m, 2H), 6.84–6.65 (m, 1H), 5.16–4.35 (m, 6H), 3.24–3.17 (m, 3H), 3.12–2.92 (m, 4H), 2.86–2.74 (m, 1H), 2.70–2.51 (m, 2H), 2.41–2.28 (m, 1H), 2.28–2.01 (m, 4H), 2.00–1.88 (m, 1H), 1.86–1.61 (m, 5H), 1.45–1.29 (m, 9H).

#### *tert*-Butyl ((5*S*,8*S*,10*aR*)-8-(((*S*)-5-Amino-5-oxo-1-phenoxypentan-2-yl)­carbamoyl)-6-oxodecahydropyrrolo­[1,2-*a*]­[1,5]­diazocin-5-yl)­carbamate (**S11g**)

This intermediate was synthesized by the same procedure as that of **S11a** as a white solid. UPLC-MS (ESI) *m*/*z*: calcd, 517.29 for C_26_H_39_N_5_O_6_ [M + H]^+^; found, 518.33. ^1^H NMR (400 MHz, DMSO-*d*_6_) δ 8.73–8.56 (m, 1H), 7.43–7.24 (m, 3H), 7.17 (s, 1H), 6.98–6.86 (m, 3H), 6.77 (s, 1H), 5.05–4.79 (m, 1H), 4.68–4.39 (m, 2H), 4.09–4.03 (m, 1H), 4.01–3.92 (m, 1H), 3.92–3.84 (m, 1H), 3.34–3.21 (m, 1H), 3.01 (t, *J* = 12.3 Hz, 1H), 2.77–2.54 (m, 2H), 2.35–1.60 (m, 11H), 1.38 (s, 9H).

#### *tert*-Butyl ((5*S*,8*S*,10*aR*)-8-(((*S*)-5-Amino-1-(2-(methylsulfonyl)­phenoxy)-5-oxopentan-2-yl)­carbamoyl)-6-oxodecahydropyrrolo­[1,2-*a*]­[1,5]­diazocin-5-yl)­carbamate (**S11h**)

This intermediate was synthesized by the same procedure as that of **S11a** as a white solid. UPLC-MS (ESI) *m*/*z*: calcd, 595.27 for C_27_H_41_N_5_O_8_S [M + H]^+^; found, 596.31. ^1^H NMR (400 MHz, DMSO-*d*_6_) δ 8.78–8.57 (m, 1H), 7.89–7.76 (m, 1H), 7.74–7.62 (m, 1H), 7.43–7.27 (m, 2H), 7.22–7.08 (m, 2H), 6.78 (s, 1H), 5.09–4.78 (m, 1H), 4.69–4.52 (m, 1H), 4.52–4.41 (m, 1H), 4.27–4.12 (m, 2H), 4.11–4.02 (m, 1H), 3.35–3.29 (m, 1H), 3.27 (s, 3H), 3.07–2.95 (m, 1H), 2.69–2.53 (m, 2H), 2.36–2.13 (m, 3H), 2.11–1.81 (m, 5H), 1.81–1.60 (m, 3H), 1.38 (s, 9H).

#### *tert*-Butyl ((5*S*,8*S*,10*aR*)-8-(((*S*)-5-Amino-1-(3-(methylsulfonyl)­phenoxy)-5-oxopentan-2-yl)­carbamoyl)-6-oxodecahydropyrrolo­[1,2-*a*]­[1,5]­diazocin-5-yl)­carbamate (**S11i**)

This intermediate was synthesized by the same procedure as that of **S11a** as a white solid. UPLC-MS (ESI) *m*/*z*: calcd, 595.27 for C_27_H_41_N_5_O_8_S [M + H]^+^; found, 596.34. ^1^H NMR (400 MHz, DMSO-*d*_6_) δ 8.73–8.60 (m, 1H), 7.64–7.48 (m, 2H), 7.46–7.40 (m, 1H), 7.35–7.24 (m, 2H), 7.18 (s, 1H), 6.78 (s, 1H), 5.00–4.80 (m, 1H), 4.61–4.40 (m, 2H), 4.24–4.04 (m, 3H), 3.57–3.36 (m, 2H), 3.34–3.25 (m, 1H), 3.23 (s, 3H), 3.08–2.94 (m, 1H), 2.35–2.21 (m, 1H), 2.20–2.00 (m, 3H), 1.99–1.81 (m, 3H), 1.81–1.63 (m, 4H), 1.38 (s, 9H).

#### *tert*-Butyl ((5*S*,8*S*,10*aR*)-8-(((*S*)-5-Amino-1-(4-(methylsulfonyl)­phenoxy)-5-oxopentan-2-yl)­carbamoyl)-6-oxodecahydropyrrolo­[1,2-*a*]­[1,5]­diazocin-5-yl)­carbamate (**S11j**)

This intermediate was synthesized by the same procedure as that of **S11a** as a white solid. UPLC-MS (ESI) *m*/*z*: calcd, 595.27 for C_27_H_41_N_5_O_8_S [M + H]^+^; found, 596.35. ^1^H NMR (400 MHz, DMSO-*d*_6_) δ 8.72–8.63 (m, 1H), 7.91–7.76 (m, 2H), 7.39–7.27 (m, 1H), 7.23–7.11 (m, 3H), 6.78 (s, 1H), 5.03–4.82 (m, 1H), 4.63–4.39 (m, 2H), 4.16–3.94 (m, 3H), 3.36–3.21 (m, 3H), 3.16 (s, 3H), 3.08–2.94 (m, 1H), 2.41–2.21 (m, 2H), 2.19–2.00 (m, 2H), 1.99–1.61 (m, 7H), 1.38 (s, 9H).

#### *tert*-Butyl ((5*S*,8*S*,10*aR*)-8-(((*S*)-5-Amino-1-(benzylamino)-1,5-dioxopentan-2-yl)­carbamoyl)-3-(6-((*R*)-2-((*S*)-2,6-dioxopiperidin-3-yl)-1-oxo-2,3,5*a*,6,8,9-hexahydro-1*H*-pyrazino­[1′,2′:4,5]­[1,4]­oxazino­[2,3-*e*]­isoindol-7­(5*H*)-yl)-6-oxohexanoyl)-6-oxodecahydropyrrolo­[1,2-*a*]­[1,5]­diazocin-5-yl)­carbamate (**S12a**)

To a stirred solution of **S2d** (100 mg, 0.2 mmol, 1 equiv) in 2 mL of DMF, DIPEA (0.17 mL, 1 mmol, 5 equiv) and HATU (76 mg, 0.2 mmol, 1 equiv) were added, and the mixture was stirred for 30 min. Then, **S11a** was added, the mixture was stirred for 1 h, and purified using preparative HPLC with acetonitrile/H_2_O to get **S12a** as a white solid. UPLC-MS (ESI) *m*/*z*: calcd, 1010.49 for C_51_H_66_N_10_O_12_ [M + H]^+^; found, 1011.64. ^1^H NMR (400 MHz, DMSO-*d*_6_) δ 10.93 (s, 1H), 8.87 (s, 1H), 8.36 (t, *J* = 6.0 Hz, 1H), 8.18 (d, *J* = 7.9 Hz, 1H), 7.36–7.16 (m, 7H), 6.79–6.51 (m, 2H), 5.03 (dd, *J* = 13.3, 5.1 Hz, 1H), 4.82–4.71 (m, 1H), 4.55–3.87 (m, 12H), 3.76–3.58 (m, 2H), 3.29–3.05 (m, 1H), 2.90 (ddd, *J* = 18.3, 14.6, 5.4 Hz, 1H), 2.82–2.53 (m, 3H), 2.48–2.33 (m, 4H), 2.21–2.04 (m, 4H), 2.02–1.48 (m, 14H), 1.37 (s, 9H).

#### *tert*-Butyl ((5*S*,8*S*,10*aR*)-8-(((*S*)-5-Amino-1-((4-(methylsulfonyl)­benzyl)­amino)-1,5-dioxopentan-2-yl)­carbamoyl)-3-(6-((*R*)-2-((*S*)-2,6-dioxopiperidin-3-yl)-1-oxo-2,3,5*a*,6,8,9-hexahydro-1*H*-pyrazino­[1′,2′:4,5]­[1,4]­oxazino­[2,3-*e*]­isoindol-7­(5*H*)-yl)-6-oxohexanoyl)-6-oxodecahydropyrrolo­[1,2-*a*]­[1,5]­diazocin-5-yl)­carbamate (**S12b**)

This intermediate was synthesized by the same procedure as that of **S12a** as a white solid. UPLC-MS (ESI) *m*/*z*: calcd, 1088.46 for C_52_H_68_N_10_O_14_S [M + H]^+^; found, 1089.59. ^1^H NMR (400 MHz, DMSO-*d*_6_) δ 10.93 (s, 1H), 8.57–8.42 (m, 1H), 8.31–8.09 (m, 1H), 7.89–7.81 (m, 2H), 7.56–7.46 (m, 2H), 7.31–7.15 (m, 2H), 7.12–7.03 (m, 1H), 6.82–6.64 (m, 1H), 6.63–6.45 (m, 1H), 5.03 (dd, *J* = 13.3, 5.1 Hz, 1H), 4.69–3.80 (m, 13H), 3.75–3.57 (m, 2H), 3.18 (s, 3H), 3.15–3.04 (m, 1H), 2.99–2.83 (m, 2H), 2.81–2.69 (m, 1H), 2.69–2.53 (m, 2H), 2.47–2.31 (m, 3H), 2.27–2.08 (m, 4H), 2.05–1.88 (m, 4H), 1.87–1.71 (m, 4H), 1.68–1.48 (m, 7H), 1.37 (s, 9H).

#### *tert*-Butyl ((5*S*,8*S*,10*aR*)-8-(((*S*)-5-Amino-1-((4-chlorobenzyl)­amino)-1,5-dioxopentan-2-yl)­carbamoyl)-3-(6-((*R*)-2-((*S*)-2,6-dioxopiperidin-3-yl)-1-oxo-2,3,5*a*,6,8,9-hexahydro-1*H*-pyrazino­[1′,2′:4,5]­[1,4]­oxazino­[2,3-*e*]­isoindol-7­(5*H*)-yl)-6-oxohexanoyl)-6-oxodecahydropyrrolo­[1,2-*a*]­[1,5]­diazocin-5-yl)­carbamate (**S12c**)

This intermediate was synthesized by the same procedure as that of **S12a** as a white solid. UPLC-MS (ESI) *m*/*z*: calcd, 1044.45 for C_51_H_65_ClN_10_O_12_ [M + H]^+^; found, 1045.68. ^1^H NMR (400 MHz, DMSO-*d*_6_) δ 10.94 (s, 1H), 8.59–8.29 (m, 1H), 8.27–8.08 (m, 1H), 7.40–7.32 (m, 2H), 7.30–7.24 (m, 2H), 7.23–7.17 (m, 2H), 7.12–7.02 (m, 1H), 6.83–6.64 (m, 1H), 6.62–6.48 (m, 1H), 5.12–4.98 (m, 1H), 4.53–3.88 (m, 13H), 3.76–3.60 (m, 2H), 3.30–3.05 (m, 1H), 2.99–2.84 (m, 1H), 2.82–2.55 (m, 3H), 2.48–2.30 (m, 4H), 2.27–2.09 (m, 4H), 2.04–1.49 (m, 15H), 1.38 (s, 9H).

#### *tert*-Butyl ((5*S*,8*S*,10*aR*)-8-(((*S*)-5-Amino-1-((4-fluorobenzyl)­amino)-1,5-dioxopentan-2-yl)­carbamoyl)-3-(6-((*R*)-2-((*S*)-2,6-dioxopiperidin-3-yl)-1-oxo-2,3,5*a*,6,8,9-hexahydro-1*H*-pyrazino­[1′,2′:4,5]­[1,4]­oxazino­[2,3-*e*]­isoindol-7­(5*H*)-yl)-6-oxohexanoyl)-6-oxodecahydropyrrolo­[1,2-*a*]­[1,5]­diazocin-5-yl)­carbamate (**S12d**)

This intermediate was synthesized by the same procedure as that of **S12a** as a white solid. UPLC-MS (ESI) *m*/*z*: calcd, 1028.48 for C_51_H_65_FN_10_O_12_ [M + H]^+^; found, 1029.68. ^1^H NMR (400 MHz, DMSO-*d*_6_) δ 10.93 (s, 1H), 8.56–8.29 (m, 1H), 8.24–8.03 (m, 1H), 7.32–7.23 (m, 2H), 7.22–7.16 (m, 2H), 7.16–7.09 (m, 2H), 7.08–7.04 (m, 1H), 6.79–6.38 (m, 2H), 5.15–4.93 (m, 1H), 4.56–3.84 (m, 13H), 3.79–3.56 (m, 2H), 3.27–3.00 (m, 1H), 2.95–2.84 (m, 1H), 2.81–2.54 (m, 3H), 2.47–2.28 (m, 4H), 2.26–2.08 (m, 4H), 2.03–1.47 (m, 15H), 1.37 (s, 9H).

#### *tert*-Butyl ((5*S*,8*S*,10*aR*)-8-(((*S*)-5-Amino-1-((4-(dimethylamino)­benzyl)­amino)-1,5-dioxopentan-2-yl)­carbamoyl)-3-(6-((*R*)-2-((*S*)-2,6-dioxopiperidin-3-yl)-1-oxo-2,3,5*a*,6,8,9-hexahydro-1*H*-pyrazino­[1′,2′:4,5]­[1,4]­oxazino­[2,3-*e*]­isoindol-7­(5*H*)-yl)-6-oxohexanoyl)-6-oxodecahydropyrrolo­[1,2-*a*]­[1,5]­diazocin-5-yl)­carbamate (**S12e**)

This intermediate was synthesized by the same procedure as that of **S12a** as a white solid. UPLC-MS (ESI) *m*/*z*: calcd, 1053.53 for C_53_H_71_N_11_O_12_ [M + H]^+^; found, 1054.74. ^1^H NMR (400 MHz, DMSO-*d*_6_) δ 10.93 (s, 1H), 8.87–8.68 (m, 1H), 8.66–8.34 (m, 1H), 8.27–8.04 (m, 2H), 7.24–7.15 (m, 2H), 7.11–7.03 (m, 3H), 6.83–6.45 (m, 2H), 5.09–4.97 (m, 1H), 4.53–3.86 (m, 13H), 3.73–3.61 (m, 2H), 3.26–3.02 (m, 1H), 2.95–2.88 (m, 1H), 2.86 (s, 6H), 2.78–2.54 (m, 3H), 2.43–2.19 (m, 4H), 2.19–2.02 (m, 4H), 2.01–1.45 (m, 15H), 1.40–1.31 (m, 9H).

#### *tert*-Butyl ((5*S*,8*S*,10*aR*)-8-(((*S*)-5-Amino-1-(methyl­(4-(methylsulfonyl)­benzyl)­amino)-1,5-dioxopentan-2-yl)­carbamoyl)-3-(6-((*R*)-2-((*S*)-2,6-dioxopiperidin-3-yl)-1-oxo-2,3,5*a*,6,8,9-hexahydro-1*H*-pyrazino­[1′,2′:4,5]­[1,4]­oxazino­[2,3-*e*]­isoindol-7­(5*H*)-yl)-6-oxohexanoyl)-6-oxodecahydropyrrolo­[1,2-*a*]­[1,5]­diazocin-5-yl)­carbamate (**S12f**)

This intermediate was synthesized by the same procedure as that of **S12a** as a white solid. UPLC-MS (ESI) *m*/*z*: calcd, 1102.48 for C_53_H_70_N_10_O_14_S [M + H]^+^; found, 1103.01. ^1^H NMR (400 MHz, DMSO-*d*_6_) δ 10.93 (s, 1H), 8.44–8.22 (m, 1H), 7.97–7.79 (m, 2H), 7.56–7.38 (m, 2H), 7.28–7.13 (m, 2H), 7.12–7.02 (m, 1H), 6.82–6.66 (m, 1H), 6.65–6.48 (m, 1H), 5.12–4.88 (m, 1H), 4.80–3.88 (m, 12H), 3.72–3.54 (m, 3H), 3.25–3.13 (m, 4H), 3.12–2.98 (m, 3H), 2.95–2.83 (m, 1H), 2.82–2.69 (m, 1H), 2.68–2.54 (m, 2H), 2.48–2.30 (m, 5H), 2.28–2.09 (m, 3H), 2.04–1.46 (m, 15H), 1.44–1.27 (m, 9H).

#### *tert*-Butyl ((5*S*,8*S*,10*aR*)-8-(((*S*)-5-Amino-5-oxo-1-phenoxypentan-2-yl)­carbamoyl)-3-(6-((*R*)-2-((*S*)-2,6-dioxopiperidin-3-yl)-1-oxo-2,3,5*a*,6,8,9-hexahydro-1*H*-pyrazino­[1′,2′:4,5]­[1,4]­oxazino­[2,3-*e*]­isoindol-7­(5*H*)-yl)-6-oxohexanoyl)-6-oxodecahydropyrrolo­[1,2-*a*]­[1,5]­diazocin-5-yl)­carbamate (**S12g**)

This intermediate was synthesized by the same procedure as that of **S12a** as a white solid. UPLC-MS (ESI) *m*/*z*: calcd, 983.48 for C_50_H_65_N_9_O_12_ [M + H]^+^; found, 984.72. ^1^H NMR (400 MHz, DMSO-*d*_6_) δ 10.94 (s, 1H), 8.12–7.90 (m, 1H), 7.37–7.23 (m, 2H), 7.23–7.12 (m, 2H), 7.11–7.01 (m, 1H), 6.99–6.87 (m, 3H), 6.80–6.69 (m, 1H), 6.68–6.58 (m, 1H), 5.03 (dd, *J* = 13.3, 5.1 Hz, 1H), 4.63–3.82 (m, 13H), 3.74–3.57 (m, 1H), 3.31–3.02 (m, 1H), 2.96–2.83 (m, 2H), 2.82–2.53 (m, 4H), 2.40 (d, *J* = 13.9 Hz, 5H), 2.25–2.10 (m, 2H), 2.02–1.49 (m, 13H), 1.37 (s, 9H), 1.29–1.21 (m, 2H).

#### *tert*-Butyl ((5*S*,8*S*,10*aR*)-8-(((*S*)-5-Amino-1-(2-(methylsulfonyl)­phenoxy)-5-oxopentan-2-yl)­carbamoyl)-3-(6-((*R*)-2-((*S*)-2,6-dioxopiperidin-3-yl)-1-oxo-2,3,5*a*,6,8,9-hexahydro-1*H*-pyrazino­[1′,2′:4,5]­[1,4]­oxazino­[2,3-*e*]­isoindol-7­(5*H*)-yl)-6-oxohexanoyl)-6-oxodecahydropyrrolo­[1,2-*a*]­[1,5]­diazocin-5-yl)­carbamate (**S12h**)

This intermediate was synthesized by the same procedure as that of **S12a** as a white solid. UPLC-MS (ESI) *m*/*z*: calcd, 1061.45 for C_51_H_67_N_9_O_14_S [M + H]^+^; found, 1062.62. ^1^H NMR (400 MHz, DMSO-*d*_6_) δ 10.95 (s, 1H), 8.43–7.93 (m, 1H), 7.88–7.75 (m, 1H), 7.74–7.57 (m, 1H), 7.34–7.25 (m, 1H), 7.23–7.10 (m, 3H), 7.10–7.01 (m, 1H), 6.86–6.46 (m, 2H), 5.03 (dd, *J* = 13.6, 5.1 Hz, 1H), 4.67–3.81 (m, 14H), 3.79–3.52 (m, 2H), 3.25 (s, 3H), 3.21–3.03 (m, 2H), 2.97–2.82 (m, 1H), 2.82–2.53 (m, 2H), 2.46–2.30 (m, 5H), 2.27–2.14 (m, 2H), 2.10–1.47 (m, 13H), 1.37 (d, *J* = 4.7 Hz, 9H), 1.25 (t, *J* = 5.6 Hz, 2H).

#### *tert*-Butyl ((5*S*,8*S*,10*aR*)-8-(((*S*)-5-Amino-1-(3-(methylsulfonyl)­phenoxy)-5-oxopentan-2-yl)­carbamoyl)-3-(6-((*R*)-2-((*S*)-2,6-dioxopiperidin-3-yl)-1-oxo-2,3,5*a*,6,8,9-hexahydro-1*H*-pyrazino­[1′,2′:4,5]­[1,4]­oxazino­[2,3-*e*]­isoindol-7­(5*H*)-yl)-6-oxohexanoyl)-6-oxodecahydropyrrolo­[1,2-*a*]­[1,5]­diazocin-5-yl)­carbamate (**S12i**)

This intermediate was synthesized by the same procedure as that of **S12a** as a white solid. UPLC-MS (ESI) *m*/*z*: calcd, 1061.45 for C_51_H_67_N_9_O_14_S [M + H]^+^; found, 1062.66. ^1^H NMR (400 MHz, DMSO-*d*_6_) δ 10.94 (s, 1H), 8.19–7.97 (m, 1H), 7.67–7.39 (m, 3H), 7.35–7.24 (m, 1H), 7.23–7.14 (m, 2H), 7.10–6.99 (m, 1H), 6.81–6.49 (m, 2H), 5.03 (dd, *J* = 13.3, 5.1 Hz, 1H), 4.58–3.87 (m, 13H), 3.30–3.03 (m, 6H), 2.97–2.83 (m, 1H), 2.82–2.54 (m, 3H), 2.47–2.28 (m, 4H), 2.27–2.08 (m, 4H), 2.00–1.47 (m, 15H), 1.37 (s, 9H).

#### *tert*-Butyl ((5*S*,8*S*,10*aR*)-8-(((*S*)-5-Amino-1-(4-(methylsulfonyl)­phenoxy)-5-oxopentan-2-yl)­carbamoyl)-3-(6-((*R*)-2-((*S*)-2,6-dioxopiperidin-3-yl)-1-oxo-2,3,5*a*,6,8,9-hexahydro-1*H*-pyrazino­[1′,2′:4,5]­[1,4]­oxazino­[2,3-*e*]­isoindol-7­(5*H*)-yl)-6-oxohexanoyl)-6-oxodecahydropyrrolo­[1,2-*a*]­[1,5]­diazocin-5-yl)­carbamate (**S12j**)

This intermediate was synthesized by the same procedure as that of **S12a** as a white solid. UPLC-MS (ESI) *m*/*z*: calcd, 1061.45 for C_51_H_67_N_9_O_14_S [M + H]^+^; found, 1062.59. ^1^H NMR (400 MHz, DMSO-*d*_6_) δ 10.95 (d, *J* = 2.8 Hz, 1H), 8.16–7.96 (m, 1H), 7.87–7.79 (m, 2H), 7.22–7.02 (m, 5H), 6.83–6.54 (m, 2H), 5.12–4.90 (m, 1H), 4.57–3.81 (m, 13H), 3.33–3.02 (m, 7H), 2.95–2.84 (m, 1H), 2.81–2.53 (m, 3H), 2.46–2.27 (m, 4H), 2.26–2.11 (m, 3H), 2.08–1.44 (m, 15H), 1.37 (s, 9H).

#### (2-(((5*S*,8*S*,10*aR*)-8-(((*S*)-5-Amino-1-((benzylamino)-1,5-dioxopentan-2-yl)­carbamoyl)-3-(6-((*R*)-2-((*S*)-2,6-dioxopiperidin-3-yl)-1-oxo-2,3,5*a*,6,8,9-hexahydro-1*H*-pyrazino­[1′,2′:4,5]­[1,4]­oxazino­[2,3-*e*]­isoindol-7­(5*H*)-yl)-6-oxohexanoyl)-6-oxodecahydropyrrolo­[1,2-*a*]­[1,5]­diazocin-5-yl)­carbamoyl)­benzo­[*b*]­thiophen-5-yl)­difluoromethyl)­phosphonic Acid (**17**)

**S12a** (100 mg, 0.1 mmol, 1 equiv) was dissolved in 2 mL DCM, and 2 mL TFA was added. The mixture was stirred for 30 min, then the solvent was removed under vacuum to get the crude residue without further purification. The crude residue was dissolved in 2 mL of DMF, DIPEA (0.17 mL, 1 mmol, 10 equiv), **S6** (57 mg, 0.12 mmol, 1.2 equiv), and HOBt (16.2 mg, 0.12 mmol, 1.2 equiv) were added, and the mixture was stirred for 2 h. The mixture was purified using preparative HPLC with acetonitrile/H_2_O (0.1% TFA) to get **17** as a white solid. UPLC-MS (ESI) *m*/*z*: calcd, 601.21 for C_56_H_63_F_2_N_10_O_14_PS [M + 2H]^+^/2; found, 601.40. ^1^H NMR (400 MHz, DMSO-*d*_6_/D_2_O) δ 8.44–8.31 (m, 1H), 8.29–8.21 (m, 1H), 8.11–7.92 (m, 2H), 7.68–7.55 (m, 1H), 7.33–7.15 (m, 5H), 7.09–6.94 (m, 1H), 5.15–4.90 (m, 2H), 4.56–4.36 (m, 4H), 4.33–4.15 (m, 5H), 4.15–3.81 (m, 3H), 3.16–3.00 (m, 1H), 2.96–2.82 (m, 1H), 2.81–2.54 (m, 5H), 2.46–2.28 (m, 6H), 2.24–2.06 (m, 4H), 2.04–1.85 (m, 3H), 1.84–1.43 (m, 10H).

#### ((2-(((5*S*,8*S*,10*aR*)-8-(((*S*)-5-Amino-1-((4-(methylsulfonyl)­benzyl)­amino)-1,5-dioxopentan-2-yl)­carbamoyl)-3-(6-((*R*)-2-((*S*)-2,6-dioxopiperidin-3-yl)-1-oxo-2,3,5*a*,6,8,9-hexahydro-1*H*-pyrazino­[1′,2′:4,5]­[1,4]­oxazino­[2,3-*e*]­isoindol-7­(5*H*)-yl)-6-oxohexanoyl)-6-oxodecahydropyrrolo­[1,2-*a*]­[1,5]­diazocin-5-yl)­carbamoyl)­benzo­[*b*]­thiophen-5-yl)­difluoromethyl)­phosphonic Acid (**18**)

This compound was synthesized by the same procedure as that of **17** as a white solid. UPLC-MS (ESI) *m*/*z*: calcd, 640.20 for C_57_H_65_F_2_N_10_O_16_PS_2_ [M + 2H]^+^/2; found, 639.69. ^1^H NMR (400 MHz, DMSO-*d*_6_/D_2_O) δ 8.33–8.24 (m, 1H), 8.13–8.06 (m, 2H), 7.89–7.76 (m, 2H), 7.60–7.56 (m, 1H), 7.54–7.43 (m, 2H), 7.23–7.13 (m, 1H), 7.09–6.95 (m, 1H), 5.13–4.71 (m, 2H), 4.52–4.32 (m, 5H), 4.30–4.14 (m, 2H), 4.14–3.83 (m, 7H), 3.28–3.01 (m, 4H), 2.98–2.80 (m, 1H), 2.80–2.54 (m, 3H), 2.44–2.29 (m, 4H), 2.26–2.06 (m, 3H), 2.04–1.85 (m, 4H), 1.83–1.43 (m, 12H).

#### ((2-(((5*S*,8*S*,10*aR*)-8-(((*S*)-5-Amino-1-((4-chlorobenzyl)­amino)-1,5-dioxopentan-2-yl)­carbamoyl)-3-(6-((*R*)-2-((*S*)-2,6-dioxopiperidin-3-yl)-1-oxo-2,3,5*a*,6,8,9-hexahydro-1*H*-pyrazino­[1′,2′:4,5]­[1,4]­oxazino­[2,3-*e*]­isoindol-7­(5*H*)-yl)-6-oxohexanoyl)-6-oxodecahydropyrrolo­[1,2-*a*]­[1,5]­diazocin-5-yl)­carbamoyl)­benzo­[*b*]­thiophen-5-yl)­difluoromethyl)­phosphonic Acid (**19**)

This compound was synthesized by the same procedure as that of **17** as a white solid. UPLC-MS (ESI) *m*/*z*: calcd, 618.19 for C_56_H_62_ClF_2_N_10_O_14_PS [M + 2H]^+^/2; found, 617.87. ^1^H NMR (400 MHz, DMSO-*d*_6_/D_2_O) δ 8.33–8.25 (m, 1H), 8.15–8.05 (m, 2H), 7.66–7.55 (m, 1H), 7.37–7.29 (m, 2H), 7.28–7.22 (m, 2H), 7.22–7.15 (m, 1H), 7.08–6.97 (m, 1H), 5.10–4.71 (m, 2H), 4.52–4.31 (m, 3H), 4.31–4.14 (m, 5H), 4.14–3.78 (m, 6H), 3.29–3.13 (m, 1H), 2.96–2.81 (m, 1H), 2.80–2.53 (m, 3H), 2.47–2.30 (m, 4H), 2.25–2.05 (m, 3H), 2.03–1.84 (m, 4H), 1.83–1.45 (m, 12H).

#### ((2-(((5*S*,8*S*,10*aR*)-8-(((*S*)-5-Amino-1-((4-fluorobenzyl)­amino)-1,5-dioxopentan-2-yl)­carbamoyl)-3-(6-((*R*)-2-((*S*)-2,6-dioxopiperidin-3-yl)-1-oxo-2,3,5*a*,6,8,9-hexahydro-1*H*-pyrazino­[1′,2′:4,5]­[1,4]­oxazino­[2,3-*e*]­isoindol-7­(5*H*)-yl)-6-oxohexanoyl)-6-oxodecahydropyrrolo­[1,2-*a*]­[1,5]­diazocin-5-yl)­carbamoyl)­benzo­[*b*]­thiophen-5-yl)­difluoromethyl)­phosphonic Acid (**20**)

This compound was synthesized by the same procedure as that of **17** as a white solid. UPLC-MS (ESI) *m*/*z*: calcd, 610.20 for C_56_H_62_F_3_N_10_O_14_PS [M + 2H]^+^/2; found, 610.06. ^1^H NMR (400 MHz, DMSO-*d*_6_/D_2_O) δ 8.30–8.25 (m, 1H), 8.15–8.06 (m, 2H), 7.64–7.51 (m, 1H), 7.31–7.15 (m, 3H), 7.15–6.97 (m, 3H), 5.06–4.71 (m, 2H), 4.51–4.34 (m, 4H), 4.32–4.15 (m, 5H), 4.14–3.81 (m, 5H), 3.26–3.03 (m, 1H), 2.96–2.69 (m, 2H), 2.68–2.54 (m, 2H), 2.46–2.28 (m, 4H), 2.21–2.05 (m, 4H), 2.04–1.83 (m, 4H), 1.83–1.45 (m, 11H).

#### ((2-(((5*S*,8*S*,10*aR*)-8-(((*S*)-5-Amino-1-((4-(dimethylamino)­benzyl)­amino)-1,5-dioxopentan-2-yl)­carbamoyl)-3-(6-((*R*)-2-((*S*)-2,6-dioxopiperidin-3-yl)-1-oxo-2,3,5*a*,6,8,9-hexahydro-1*H*-pyrazino­[1′,2′:4,5]­[1,4]­oxazino­[2,3-*e*]­isoindol-7­(5*H*)-yl)-6-oxohexanoyl)-6-oxodecahydropyrrolo­[1,2-*a*]­[1,5]­diazocin-5-yl)­carbamoyl)­benzo­[*b*]­thiophen-5-yl)­difluoromethyl)­phosphonic Acid (**21**)

This compound was synthesized by the same procedure as that of **17** as a white solid. UPLC-MS (ESI) *m*/*z*: calcd, 622.73 for C_58_H_68_F_2_N_11_O_14_PS [M + 2H]^+^/2; found, 622.70. ^1^H NMR (400 MHz, DMSO-*d*_6_/D_2_O) δ 8.31–8.20 (m, 1H), 8.16–8.02 (m, 2H), 7.65–7.55 (m, 1H), 7.36–7.24 (m, 4H), 7.23–7.14 (m, 1H), 7.07–6.95 (m, 1H), 5.16–4.74 (m, 2H), 4.49–4.35 (m, 3H), 4.35–4.21 (m, 4H), 4.20–4.07 (m, 5H), 4.01–3.80 (m, 3H), 3.77–3.54 (m, 1H), 3.51–3.31 (m, 1H), 3.30–3.13 (m, 1H), 3.02 (s, 6H), 2.93–2.68 (m, 2H), 2.67–2.54 (m, 2H), 2.48–2.28 (m, 4H), 2.24–2.07 (m, 4H), 2.05–1.46 (m, 12H).

#### ((2-(((5*S*,8*S*,10*aR*)-8-(((*S*)-5-Amino-1-(methyl­(4-(methylsulfonyl)­benzyl)­amino)-1,5-dioxopentan-2-yl)­carbamoyl)-3-(6-((*R*)-2-((*S*)-2,6-dioxopiperidin-3-yl)-1-oxo-2,3,5*a*,6,8,9-hexahydro-1*H*-pyrazino­[1′,2′:4,5]­[1,4]­oxazino­[2,3-*e*]­isoindol-7­(5*H*)-yl)-6-oxohexanoyl)-6-oxodecahydropyrrolo­[1,2-*a*]­[1,5]­diazocin-5-yl)­carbamoyl)­benzo­[*b*]­thiophen-5-yl)­difluoromethyl)­phosphonic Acid (**22**)

This compound was synthesized by the same procedure as that of **17** as a white solid. UPLC-MS (ESI) *m*/*z*: calcd, 647.2 for C_58_H_67_F_2_N_10_O_16_PS_2_ [M + 2H]^+^/2; found, 647.27. ^1^H NMR (400 MHz, DMSO-*d*_6_/D_2_O) δ 8.32–8.19 (m, 1H), 8.15–8.04 (m, 2H), 7.93–7.80 (m, 2H), 7.64–7.55 (m, 1H), 7.54–7.36 (m, 2H), 7.25–7.12 (m, 1H), 7.08–6.93 (m, 1H), 5.04–4.77 (m, 2H), 4.76–4.61 (m, 2H), 4.60–4.34 (m, 4H), 4.32–4.16 (m, 2H), 4.15–4.05 (m, 1H), 4.04–3.87 (m, 5H), 3.74–3.54 (m, 1H), 3.51–3.30 (m, 1H), 3.26–3.12 (m, 4H), 3.11–2.95 (m, 3H), 2.94–2.68 (m, 2H), 2.65–2.54 (m, 2H), 2.47–2.29 (m, 4H), 2.27–2.07 (m, 3H), 2.07–1.83 (m, 4H), 1.64 (d, *J* = 74.4 Hz, 10H).

#### ((2-(((5*S*,8*S*,10*aR*)-8-(((*S*)-5-Amino-5-oxo-1-phenoxypentan-2-yl)­carbamoyl)-3-(6-((*R*)-2-((*S*)-2,6-dioxopiperidin-3-yl)-1-oxo-2,3,5*a*,6,8,9-hexahydro-1*H*-pyrazino­[1′,2′:4,5]­[1,4]­oxazino­[2,3-*e*]­isoindol-7­(5*H*)-yl)-6-oxohexanoyl)-6-oxodecahydropyrrolo­[1,2-*a*]­[1,5]­diazocin-5-yl)­carbamoyl)­benzo­[*b*]­thiophen-5-yl)­difluoromethyl)­phosphonic Acid (**23**)

This compound was synthesized by the same procedure as that of **17** as a white solid. UPLC-MS (ESI) *m*/*z*: calcd, 1173.38 for C_55_H_62_F_2_N_9_O_14_PS [M + H]^+^/2; found, 1174.27. ^1^H NMR (400 MHz, DMSO-*d*_6_/D_2_O) δ 8.33–8.23 (m, 1H), 8.17–8.07 (m, 2H), 7.61–7.54 (m, 1H), 7.32–7.15 (m, 3H), 7.08–6.98 (m, 1H), 6.96–6.87 (m, 3H), 5.05–4.78 (m, 2H), 4.53–4.37 (m, 2H), 4.37–4.15 (m, 3H), 4.14–3.95 (m, 3H), 3.95–3.80 (m, 4H), 3.43–3.31 (m, 2H), 3.28–3.16 (m, 1H), 2.97–2.69 (m, 2H), 2.68–2.53 (m, 2H), 2.47–2.28 (m, 4H), 2.26–2.05 (m, 4H), 2.04–1.47 (m, 15H).

#### ((2-(((5*S*,8*S*,10*aR*)-8-(((*S*)-5-Amino-1-(2-(methylsulfonyl)­phenoxy)-5-oxopentan-2-yl)­carbamoyl)-3-(6-((*R*)-2-((*S*)-2,6-dioxopiperidin-3-yl)-1-oxo-2,3,5*a*,6,8,9-hexahydro-1*H*-pyrazino­[1′,2′:4,5]­[1,4]­oxazino­[2,3-*e*]­isoindol-7­(5*H*)-yl)-6-oxohexanoyl)-6-oxodecahydropyrrolo­[1,2-*a*]­[1,5]­diazocin-5-yl)­carbamoyl)­benzo­[*b*]­thiophen-5-yl)­difluoromethyl)­phosphonic Acid (**24**)

This compound was synthesized by the same procedure as that of **17** as a white solid. UPLC-MS (ESI) *m*/*z*: calcd, 626.69 for C_56_H_64_F_2_N_9_O_16_PS_2_ [M + 2H]^+^/2; found, 626.77. ^1^H NMR (400 MHz, DMSO-*d*_6_/D_2_O) δ 8.34–8.25 (m, 1H), 8.17–8.02 (m, 2H), 7.83–7.74 (m, 1H), 7.71–7.62 (m, 1H), 7.61–7.54 (m, 1H), 7.32–7.24 (m, 1H), 7.23–7.11 (m, 2H), 7.08–6.96 (m, 1H), 5.08–4.79 (m, 2H), 4.53–4.35 (m, 2H), 4.34–4.16 (m, 3H), 4.15–3.79 (m, 7H), 3.47–3.31 (m, 1H), 3.27–3.18 (m, 4H), 3.13–3.02 (m, 1H), 2.96–2.81 (m, 1H), 2.79–2.53 (m, 3H), 2.47–2.30 (m, 4H), 2.27–2.02 (m, 4H), 2.02–1.45 (m, 15H).

#### ((2-(((5*S*,8*S*,10*aR*)-8-(((*S*)-5-Amino-1-(3-(methylsulfonyl)­phenoxy)-5-oxopentan-2-yl)­carbamoyl)-3-(6-((*R*)-2-((*S*)-2,6-dioxopiperidin-3-yl)-1-oxo-2,3,5*a*,6,8,9-hexahydro-1*H*-pyrazino­[1′,2′:4,5]­[1,4]­oxazino­[2,3-*e*]­isoindol-7­(5*H*)-yl)-6-oxohexanoyl)-6-oxodecahydropyrrolo­[1,2-*a*]­[1,5]­diazocin-5-yl)­carbamoyl)­benzo­[*b*]­thiophen-5-yl)­difluoromethyl)­phosphonic Acid (**25**)

This compound was synthesized by the same procedure as that of **17** as a white solid. UPLC-MS (ESI) *m*/*z*: calcd, 626.69 for C_56_H_64_F_2_N_9_O_16_PS_2_ [M + 2H]^+^/2; found, 626.67. ^1^H NMR (400 MHz, DMSO-*d*_6_/D_2_O) δ 8.32–8.26 (m, 1H), 8.14–8.05 (m, 2H), 7.61–7.52 (m, 2H), 7.51–7.45 (m, 1H), 7.44–7.37 (m, 1H), 7.31–7.24 (m, 1H), 7.23–7.14 (m, 1H), 7.08–6.95 (m, 1H), 5.06–4.77 (m, 2H), 4.53–4.37 (m, 2H), 4.36–4.15 (m, 3H), 4.14–3.80 (m, 7H), 3.43–3.00 (m, 6H), 2.96–2.80 (m, 1H), 2.79–2.54 (m, 3H), 2.46–2.29 (m, 4H), 2.28–2.06 (m, 4H), 2.03–1.41 (m, 15H).

#### ((2-(((5*S*,8*S*,10*aR*)-8-(((*S*)-5-Amino-1-(4-(methylsulfonyl)­phenoxy)-5-oxopentan-2-yl)­carbamoyl)-3-(6-((*R*)-2-((*S*)-2,6-dioxopiperidin-3-yl)-1-oxo-2,3,5*a*,6,8,9-hexahydro-1*H*-pyrazino­[1′,2′:4,5]­[1,4]­oxazino­[2,3-*e*]­isoindol-7­(5*H*)-yl)-6-oxohexanoyl)-6-oxodecahydropyrrolo­[1,2-*a*]­[1,5]­diazocin-5-yl)­carbamoyl)­benzo­[*b*]­thiophen-5-yl)­difluoromethyl)­phosphonic Acid (**26**)

This compound was synthesized by the same procedure as that of **17** as a white solid. UPLC-MS (ESI) *m*/*z*: calcd, 626.69 for C_56_H_64_F_2_N_9_O_16_PS_2_ [M + 2H]^+^/2; found, 626.66. ^1^H NMR (400 MHz, DMSO-*d*_6_/D_2_O) δ 8.30–7.97 (m, 3H), 7.83–7.76 (m, 2H), 7.72–7.54 (m, 1H), 7.22–7.08 (m, 3H), 7.06–6.92 (m, 1H), 5.05–4.73 (m, 2H), 4.49–4.32 (m, 2H), 4.32–4.14 (m, 3H), 4.14–3.91 (m, 7H), 3.51–3.31 (m, 1H), 3.29–3.15 (m, 1H), 3.13–3.00 (m, 4H), 2.94–2.53 (m, 4H), 2.45–2.28 (m, 4H), 2.26–2.01 (m, 4H), 1.99–1.40 (m, 15H).

#### (2-(((5*S*,8*S*,10*aR*)-8-(((*S*)-5-Amino-1-(3-(methylsulfonyl)­phenoxy)-5-oxopentan-2-yl)­carbamoyl)-3-(6-((*R*)-2-((*S*)-2,6-dioxopiperidin-3-yl)-1-oxo-2,3,5*a*,6,8,9-hexahydro-1*H*-pyrazino­[1′,2′:4,5]­[1,4]­oxazino­[2,3-*e*]­isoindol-7­(5*H*)-yl)-6-oxohexanoyl)-6-oxodecahydropyrrolo­[1,2-*a*]­[1,5]­diazocin-5-yl)­carbamoyl)-1*H*-indole-5-carbonyl)­phosphonic Acid (**SD-965**)

**S6** was replaced with **S13** for the last step; the other procedure for the synthesis of this compound is the same as that of **17** to give a white solid. UPLC-MS (ESI) *m*/*z*: calcd, 607.62 for C_56_H_64_F_2_N_9_O_16_PS_2_ [M + 2H]^+^/2; found, 627.04. ^1^H NMR (400 MHz, DMSO-*d*_6_/D_2_O) δ 8.78 (s, 1H), 7.99–7.86 (m, 1H), 7.64–7.37 (m, 5H), 7.35–7.14 (m, 2H), 7.08–6.93 (m, 1H), 5.11–4.82 (m, 2H), 4.55–4.16 (m, 5H), 4.15–3.86 (m, 7H), 3.48–2.98 (m, 6H), 2.94–2.53 (m, 4H), 2.47–2.32 (m, 4H), 2.27–2.11 (m, 3H), 2.06–1.44 (m, 16H).

#### 6-(6-(2,6-Dioxopiperidin-3-yl)-5,7-dioxo-3,5,6,7-tetrahydropyrrolo­[3,4-*f*]­isoindol-2­(1*H*)-yl)-6-oxohexanoic Acid (**S16a**)

To a stirred solution of **S15** (188 mg, 1 mmol, 1 equiv) in 5 mL of DMF, DIPEA (0.52 mL, 3 mmol, 3 equiv) and HATU (380 mg, 1 mmol, 1 equiv) were added, and the mixture was stirred for 30 min. Then, **S14a** (240 mg, 0.8 mmol, 0.8 equiv) was added. This mixture was stirred for 1 h, quenched with NaHCO_3_ aqueous solution, extracted with EtOAc (5 mL × 5), dried with anhydrous sodium sulfate, filtered, and the solvent was removed under vacuum. The residual crude product was dissolved in 2 mL of MeCN, and 2 mL of TFA was added. Then, the mixture was stirred for 2 h, the solvent was removed under vacuum, and the crude residue was purified using preparative HPLC with acetonitrile/H_2_O (0.1% TFA) to get **S16a** as a white solid. UPLC-MS (ESI) *m*/*z*: calcd, 427.14 for C_21_H_21_N_3_O_7_ [M + H]^+^; found, 428.35. ^1^H NMR (400 MHz, DMSO-*d*_6_) δ 11.14 (s, 1H), 7.91 (d, *J* = 10.0 Hz, 2H), 5.15 (dd, *J* = 13.0, 5.4 Hz, 1H), 4.96 (s, 2H), 4.75 (s, 2H), 2.89 (ddd, *J* = 17.4, 14.1, 5.5 Hz, 1H), 2.68–2.52 (m, 2H), 2.43–2.34 (m, 2H), 2.30–2.20 (m, 2H), 2.13–1.98 (m, 1H), 1.73–1.43 (m, 4H).

#### 6-(6-(2,6-Dioxopiperidin-3-yl)-5-oxo-3,5,6,7-tetrahydropyrrolo­[3,4-*f*]­isoindol-2­(1*H*)-yl)-6-oxohexanoic Acid (**S16b**)

This intermediate was synthesized by the same procedure as that of **S16a** as a white solid. UPLC-MS (ESI) *m*/*z*: calcd, 413.16 for C_21_H_23_N_3_O_6_ [M + H]^+^; found, 414.34. ^1^H NMR (400 MHz, DMSO-*d*_6_) δ 11.00 (s, 1H), 7.69 (d, *J* = 10.1 Hz, 1H), 7.56 (d, *J* = 8.7 Hz, 1H), 5.11 (dd, *J* = 13.3, 5.0 Hz, 1H), 4.90 (d, *J* = 7.0 Hz, 2H), 4.70 (d, *J* = 7.3 Hz, 2H), 4.52–4.25 (m, 2H), 3.03–2.83 (m, 1H), 2.60 (d, *J* = 17.3 Hz, 1H), 2.45–2.32 (m, 3H), 2.28–2.21 (m, 2H), 2.05–1.96 (m, 1H), 1.67–1.48 (m, 4H).

#### (*S*)-6-(7-(2,6-Dioxopiperidin-3-yl)-6-oxo-7,8-dihydro-2*H*,6*H*-spiro­[furo­[2,3-*e*]­isoindole-3,4′-piperidin]-1′-yl)-6-oxohexanoic Acid (**S16c**)

This intermediate was synthesized by the same procedure as that of **S16a** as a white solid. UPLC-MS (ESI) *m*/*z*: calcd, 483.20 for C_25_H_29_N_3_O_7_ [M + H]^+^; found, 484.34. ^1^H NMR (400 MHz, DMSO-*d*_6_) δ 10.99 (s, 1H), 7.42 (d, *J* = 7.6 Hz, 1H), 7.28 (d, *J* = 7.5 Hz, 1H), 5.10 (ddd, *J* = 13.4, 5.3, 1.8 Hz, 1H), 4.69–4.59 (m, 2H), 4.44–4.35 (m, 2H), 4.23 (dd, *J* = 17.1, 3.2 Hz, 1H), 3.90 (d, *J* = 13.8 Hz, 1H), 3.16 (t, *J* = 13.0 Hz, 1H), 3.00–2.85 (m, 1H), 2.76–2.65 (m, 1H), 2.64–2.55 (m, 1H), 2.49–2.30 (m, 3H), 2.29–2.21 (m, 2H), 2.03–1.93 (m, 1H), 1.93–1.82 (m, 1H), 1.79–1.68 (m, 3H), 1.60–1.47 (m, 4H).

#### 6-(4-(1-(2,6-Dioxopiperidin-3-yl)-3-methyl-2-oxo-2,3-dihydro-1*H*-benzo­[*d*]­imidazol-4-yl)­piperidin-1-yl)-6-oxohexanoic Acid (**S16d**)

This intermediate was synthesized by the same procedure as that of **S16a** as a white solid. UPLC-MS (ESI) *m*/*z*: calcd, 470.22 for C_24_H_30_N_4_O_6_ [M + H]^+^; found, 471.07. ^1^H NMR (400 MHz, DMSO-*d*_6_) δ 11.09 (s, 1H), 6.99 (s, 3H), 5.45–5.28 (m, 1H), 4.60–4.53 (m, 1H), 4.04–3.95 (m, 1H), 3.62 (s, 3H), 3.56–3.46 (m, 1H), 3.23–3.12 (m, 1H), 2.95–2.82 (m, 1H), 2.78–2.58 (m, 3H), 2.40–2.32 (m, 2H), 2.28–2.18 (m, 2H), 2.05–1.94 (m, 1H), 1.92–1.79 (m, 2H), 1.73–1.63 (m, 1H), 1.60–1.47 (m, 5H).

#### 6-(4-(1-(2,6-Dioxopiperidin-3-yl)-3-methyl-2-oxo-2,3-dihydro-1*H*-benzo­[*d*]­imidazol-4-yl)­piperazin-1-yl)-6-oxohexanoic Acid (**S16e**)

This intermediate was synthesized by the same procedure as that of **S16a** as a white solid. UPLC-MS (ESI) *m*/*z*: calcd, 471.21 for C_23_H_29_N_5_O_6_ [M + H]^+^; found, 472.06. ^1^H NMR (400 MHz, DMSO-*d*_6_) δ 11.09 (s, 1H), 7.15–6.69 (m, 3H), 5.48–5.25 (m, 1H), 4.45–4.41 (m, 3H), 3.64 (s, 3H), 3.16–2.98 (m, 3H), 2.95–2.79 (m, 3H), 2.76–2.57 (m, 2H), 2.43–2.30 (m, 2H), 2.27–2.19 (m, 2H), 2.06–1.92 (m, 1H), 1.61–1.49 (m, 4H).

#### 6-(4-(1-(2,6-Dioxopiperidin-3-yl)-3-methyl-2-oxo-2,3-dihydro-1*H*-benzo­[*d*]­imidazol-5-yl)­piperidin-1-yl)-6-oxohexanoic Acid (**S16f**)

This intermediate was synthesized by the same procedure as that of **S16a** as a white solid. UPLC-MS (ESI) *m*/*z*: calcd, 470.22 for C_24_H_30_N_4_O_6_ [M + H]^+^; found, 471.06. ^1^H NMR (400 MHz, DMSO-*d*_6_) δ 11.08 (s, 1H), 7.10 (s, 1H), 7.04–6.98 (m, 1H), 6.95–6.88 (m, 1H), 5.38–5.29 (m, 1H), 4.60–4.52 (m, 1H), 4.03–3.95 (m, 1H), 3.32 (s, 3H), 3.13–3.03 (m, 1H), 2.95–2.83 (m, 1H), 2.83–2.53 (m, 4H), 2.39–2.31 (m, 2H), 2.27–2.16 (m, 2H), 2.04–1.94 (m, 1H), 1.84–1.71 (m, 3H), 1.67–1.42 (m, 5H).

#### 6-(4-(1-(2,6-Dioxopiperidin-3-yl)-3-methyl-2-oxo-2,3-dihydro-1*H*-benzo­[*d*]­imidazol-5-yl)­piperazin-1-yl)-6-oxohexanoic Acid (**S16g**)

This intermediate was synthesized by the same procedure as that of **S16a** as a white solid. UPLC-MS (ESI) *m*/*z*: calcd, 471.21 for C_23_H_29_N_5_O_6_ [M + H]^+^; found, 472.06. ^1^H NMR (400 MHz, DMSO-*d*_6_) δ 11.24–10.67 (m, 1H), 7.00–6.93 (m, 1H), 6.88 (s, 1H), 6.69–6.62 (m, 1H), 5.34–5.25 (m, 1H), 3.64–3.54 (m, 4H), 3.31 (s, 3H), 3.10–3.00 (m, 4H), 2.94–2.82 (m, 1H), 2.75–2.55 (m, 2H), 2.42–2.29 (m, 2H), 2.22–2.09 (m, 2H), 2.04–1.94 (m, 1H), 1.65–1.41 (m, 4H).

#### 6-(4-(3-(2,6-Dioxopiperidin-3-yl)-1-methyl-1*H*-indazol-7-yl)­piperidin-1-yl)-6-oxohexanoic Acid (**S16h**)

This intermediate was synthesized by the same procedure as that of **S16a** as a white solid. UPLC-MS (ESI) *m*/*z*: calcd, 454.22 for C_24_H_30_N_4_O_5_ [M + H]^+^; found, 455.05. ^1^H NMR (400 MHz, DMSO-*d*_6_) δ 10.88 (s, 1H), 7.55 (d, *J* = 8.0 Hz, 1H), 7.23 (d, *J* = 7.2 Hz, 1H), 7.06 (t, *J* = 7.6 Hz, 1H), 4.63–4.55 (m, 1H), 4.39–4.31 (m, 1H), 4.24 (s, 3H), 4.08–3.98 (m, 1H), 3.68–3.56 (m, 1H), 3.32–3.17 (m, 1H), 2.79–2.58 (m, 3H), 2.34 (ddq, *J* = 14.4, 9.9, 5.1, 4.2 Hz, 3H), 2.28–2.10 (m, 3H), 1.97–1.85 (m, 2H), 1.61–1.46 (m, 6H).

#### 6-(4-(3-(2,6-Dioxopiperidin-3-yl)-1-methyl-1*H*-indazol-7-yl)­piperazin-1-yl)-6-oxohexanoic Acid (**S16i**)

This intermediate was synthesized by the same procedure as that of **S16a** as a white solid. UPLC-MS (ESI) *m*/*z*: calcd, 455.22 for C_23_H_29_N_5_O_5_ [M + H]^+^; found, 456.01. ^1^H NMR (400 MHz, DMSO-*d*_6_) δ 10.88 (s, 1H), 7.47–7.30 (m, 1H), 7.11–6.95 (m, 2H), 4.56–4.42 (m, 2H), 4.39–4.30 (m, 1H), 4.27 (s, 3H), 4.12–3.92 (m, 2H), 3.44–3.28 (m, 2H), 3.26–3.11 (m, 2H), 2.72–2.56 (m, 2H), 2.44–2.09 (m, 6H), 1.59–1.47 (m, 4H).

#### 6-(4-(3-(2,6-Dioxopiperidin-3-yl)-1-methyl-1*H*-indazol-6-yl)­piperidin-1-yl)-6-oxohexanoic Acid (**S16j**)

This intermediate was synthesized by the same procedure as that of **S16a** as a white solid. UPLC-MS (ESI) *m*/*z*: calcd, 454.22 for C_24_H_30_N_4_O_5_ [M + H]^+^; found, 455.08. ^1^H NMR (400 MHz, DMSO-*d*_6_) δ 10.87 (s, 1H), 7.61 (d, *J* = 8.4 Hz, 1H), 7.44 (s, 1H), 7.03 (dd, *J* = 8.5, 1.3 Hz, 1H), 4.62–4.54 (m, 1H), 4.37–4.28 (m, 1H), 4.07–3.98 (m, 1H), 3.96 (s, 3H), 3.17–3.06 (m, 1H), 2.97–2.84 (m, 1H), 2.73–2.53 (m, 3H), 2.41–2.30 (m, 3H), 2.27–2.13 (m, 3H), 1.89–1.78 (m, 2H), 1.72–1.59 (m, 1H), 1.58–1.46 (m, 5H).

#### 6-(4-(3-(2,6-Dioxopiperidin-3-yl)-1-methyl-1*H*-indazol-6-yl)­piperazin-1-yl)-6-oxohexanoic Acid (**S16k**)

This intermediate was synthesized by the same procedure as that of **S16a** as a white solid. UPLC-MS (ESI) *m*/*z*: calcd, 455.22 for C_23_H_29_N_5_O_5_ [M + H]^+^; found, 456.05. ^1^H NMR (400 MHz, DMSO-*d*_6_) δ 10.87 (s, 1H), 7.66–7.57 (m, 1H), 7.21–6.96 (m, 2H), 4.38–4.26 (m, 1H), 3.95–3.89 (m, 3H), 3.75–3.61 (m, 4H), 3.41–3.23 (m, 4H), 2.71–2.55 (m, 3H), 2.40–2.29 (m, 2H), 2.28–2.06 (m, 3H), 1.57–1.47 (m, 4H).

#### 6-(4-(4-(2,6-Dioxopiperidin-3-yl)-3,5-difluorophenyl)­piperazin-1-yl)-6-oxohexanoic Acid (**S16l**)

This intermediate was synthesized by the same procedure as that of **S16a** as a white solid. UPLC-MS (ESI) *m*/*z*: calcd, 437.18 for C_21_H_25_F_2_N_3_O_5_ [M + H]^+^; found, 437.96. ^1^H NMR (400 MHz, DMSO-*d*_6_) δ 10.88 (s, 1H), 6.76–6.59 (m, 2H), 4.06 (dd, *J* = 12.7, 5.2 Hz, 1H), 3.61–3.52 (m, 4H), 3.27–3.14 (m, 4H), 2.85–2.71 (m, 1H), 2.39–2.31 (m, 2H), 2.28–2.02 (m, 4H), 2.01–1.90 (m, 1H), 1.61–1.45 (m, *J* = 4.3 Hz, 4H).

#### (S)-6-(4-(2-(2,6-Dioxopiperidin-3-yl)-1-oxoisoindolin-5-yl)­piperazin-1-yl)-6-oxohexanoic Acid (**S16m**)

This intermediate was synthesized by the same procedure as that of **S16a** as a white solid. UPLC-MS (ESI) *m*/*z*: calcd, 456.20 for C_23_H_28_N_4_O_6_ [M + H]^+^; found, 457.06. ^1^H NMR (400 MHz, DMSO-*d*_6_) δ 12.00 (s, 1H), 10.94 (s, 1H), 7.54 (d, *J* = 8.4 Hz, 1H), 7.17–6.97 (m, 2H), 5.05 (dd, *J* = 13.3, 5.1 Hz, 1H), 4.43–4.12 (m, 2H), 3.74–3.50 (m, 4H), 3.42–3.22 (m, 5H), 2.99–2.81 (m, 1H), 2.44–2.29 (m, 3H), 2.27–2.18 (m, 2H), 2.02–1.90 (m, 1H), 1.60–1.45 (m, *J* = 4.5, 4.1 Hz, 4H).

#### (2-(((5*S*,8*S*,10*aR*)-8-(((*S*)-5-Amino-1-(3-(methylsulfonyl)­phenoxy)-5-oxopentan-2-yl)­carbamoyl)-3-(6-(6-(2,6-dioxopiperidin-3-yl)-5,7-dioxo-3,5,6,7-tetrahydropyrrolo­[3,4-*f*]­isoindol-2­(1*H*)-yl)-6-oxohexanoyl)-6-oxodecahydropyrrolo­[1,2-*a*]­[1,5]­diazocin-5-yl)­carbamoyl)-1*H*-indole-5-carbonyl)­phosphonic Acid (**28**)

To a stirred solution of **S16a** (43 mg, 0.1 mmol, 1 equiv) in 2 mL of DMF, DIPEA (0.09 mL, 0.5 mmol, 5 equiv) and HATU (38 mg, 0.1 mmol, 1 equiv) were added, and the mixture was stirred for 10 min. Then, **S11** (54 mg, 0.09 mmol, 0.9 equiv) was added. The mixture was stirred for 1 h, purified by HPLC with acetonitrile/H_2_O (0.1% TFA), and concentrated for the next step. The concentrate was dissolved in 4 mL of TFA/DCM = 1:1 to remove the Boc protection group to get the corresponding primary amine. After removing TFA under vacuum, to a stirred solution of the residue (0.09 mmol, 1 equiv), compound **S13** (59 mg, 0.135 mmol, 1.5 equiv), and HOBt (18 mg, 0.135 mmol, 1.5 equiv) in DMF, DIPEA (0.16 mL, 0.9 mmol, 10 equiv) was added at room temperature. The reaction mixture was then stirred at room temperature for 2 h. After the complete disappearance of the starting amine, the product was purified using a preparative HPLC to provide compound **28** as a white solid. UPLC-MS (ESI) *m*/*z*: calcd, 1155.34 for C_53_H_58_N_9_O_17_PS [M + H]^+^; found, 1156.2. ^1^H NMR (400 MHz, DMSO-*d*_6_/D_2_O) δ 8.81–8.67 (m, 1H), 7.95–7.73 (m, 3H), 7.58–7.35 (m, 5H), 7.30–7.19 (m, 1H), 5.18–5.04 (m, 1H), 5.04–4.87 (m, 3H), 4.81–4.64 (m, 2H), 4.39–4.14 (m, 2H), 4.13–3.83 (m, 3H), 3.44–3.27 (m, 2H), 3.19 (s, 3H), 2.95–2.76 (m, 1H), 2.71–2.52 (m, 6H), 2.46–2.30 (m, 2H), 2.27–2.10 (m, 3H), 2.10–1.51 (m, 12H).

#### (2-(((5*S*,8*S*,10*aR*)-8-(((*S*)-5-Amino-1-(3-(methylsulfonyl)­phenoxy)-5-oxopentan-2-yl)­carbamoyl)-3-(6-(6-(2,6-dioxopiperidin-3-yl)-5-oxo-3,5,6,7-tetrahydropyrrolo­[3,4-*f*]­isoindol-2­(1*H*)-yl)-6-oxohexanoyl)-6-oxodecahydropyrrolo­[1,2-*a*]­[1,5]­diazocin-5-yl)­carbamoyl)-1*H*-indole-5-carbonyl)­phosphonic Acid (**29**)

This compound was synthesized by the same procedure as that of **28** as a white solid. UPLC-MS (ESI) *m*/*z*: calcd, 1141.36 for C_53_H_60_N_9_O_16_PS [M + H]^+^; found, 1142.0. ^1^H NMR (400 MHz, DMSO-*d*_6_/D_2_O) δ 8.83–8.68 (m, 1H), 7.98–7.86 (m, 1H), 7.74–7.62 (m, 1H), 7.60–7.36 (m, 6H), 7.34–7.19 (m, 1H), 5.15–4.96 (m, 2H), 4.94–4.86 (m, 2H), 4.74–4.62 (m, 2H), 4.49–4.17 (m, 4H), 4.13–3.85 (m, 4H), 3.20 (s, 3H), 2.96–2.82 (m, 1H), 2.73–2.56 (m, 5H), 2.47–2.37 (m, 3H), 2.25–2.10 (m, 2H), 2.07–1.96 (m, 2H), 1.90–1.51 (m, 10H).

#### (2-(((5*S*,8*S*,10*aR*)-8-(((*S*)-5-Amino-1-(3-(methylsulfonyl)­phenoxy)-5-oxopentan-2-yl)­carbamoyl)-3-(6-(7-((*S*)-2,6-dioxopiperidin-3-yl)-6-oxo-7,8-dihydro-2*H*,6*H*-spiro­[furo­[2,3-*e*]­isoindole-3,4′-piperidin]-1′-yl)-6-oxohexanoyl)-6-oxodecahydropyrrolo­[1,2-*a*]­[1,5]­diazocin-5-yl)­carbamoyl)-1*H*-indole-5-carbonyl)­phosphonic Acid (**30**)

This compound was synthesized by the same procedure as that of **28** as a white solid. UPLC-MS (ESI) *m*/*z*: calcd, 606.71 for C_57_H_66_N_9_O_17_PS [M + 2H]^+^/2; found, 606.70. ^1^H NMR (400 MHz, DMSO-*d*_6_/D_2_O) δ 8.77 (s, 1H), 8.00–7.85 (m, 1H), 7.60–7.33 (m, 6H), 7.28–7.19 (m, 2H), 5.08–4.93 (m, 2H), 4.63–4.56 (m, 2H), 4.41–4.26 (m, 5H), 4.26–4.16 (m, 2H), 4.07–3.96 (m, 3H), 3.92–3.81 (m, 2H), 3.21–3.16 (m, 5H), 2.94–2.81 (m, 1H), 2.74–2.54 (m, 4H), 2.43–2.30 (m, 3H), 2.24–2.11 (m, 2H), 2.07–1.95 (m, 3H), 1.93–1.82 (m, 2H), 1.78–1.50 (m, 12H).

#### (2-(((5*S*,8*S*,10*aR*)-8-(((*S*)-5-Amino-1-(3-(methylsulfonyl)­phenoxy)-5-oxopentan-2-yl)­carbamoyl)-3-(6-(4-(1-(2,6-dioxopiperidin-3-yl)-3-methyl-2-oxo-2,3-dihydro-1*H*-benzo­[*d*]­imidazol-4-yl)­piperidin-1-yl)-6-oxohexanoyl)-6-oxodecahydropyrrolo­[1,2-*a*]­[1,5]­diazocin-5-yl)­carbamoyl)-1*H*-indole-5-carbonyl)­phosphonic Acid (**31**)

This compound was synthesized by the same procedure as that of **28** as a white solid. UPLC-MS (ESI) *m*/*z*: calcd, 1198.42 for C_56_H_69_N_10_O_16_PS [M + H]^+^; found, 1198.94. ^1^H NMR (400 MHz, DMSO-*d*_6_/D_2_O) δ 8.76 (s, 1H), 8.03–7.85 (m, 1H), 7.60–7.50 (m, 2H), 7.50–7.36 (m, 3H), 7.31–7.20 (m, 1H), 7.08–6.82 (m, 3H), 5.54–5.15 (m, 1H), 5.10–4.77 (m, 1H), 4.66–4.45 (m, 1H), 4.39–4.07 (m, 3H), 4.11–3.91 (m, 4H), 3.80–3.26 (m, 5H), 3.24–3.02 (m, 4H), 2.92–2.77 (m, 1H), 2.75–2.56 (m, 4H), 2.44–2.30 (m, 2H), 2.28–2.05 (m, 3H), 2.05–1.92 (m, 3H), 1.92–1.36 (m, 16H).

#### (2-(((5*S*,8*S*,10*aR*)-8-(((*S*)-5-Amino-1-(3-(methylsulfonyl)­phenoxy)-5-oxopentan-2-yl)­carbamoyl)-3-(6-(4-(1-(2,6-dioxopiperidin-3-yl)-3-methyl-2-oxo-2,3-dihydro-1*H*-benzo­[*d*]­imidazol-4-yl)­piperazin-1-yl)-6-oxohexanoyl)-6-oxodecahydropyrrolo­[1,2-*a*]­[1,5]­diazocin-5-yl)­carbamoyl)-1*H*-indole-5-carbonyl)­phosphonic Acid (**32**)

This compound was synthesized by the same procedure as that of **28** as a white solid. UPLC-MS (ESI) *m*/*z*: calcd, 600.72 for C_55_H_66_N_11_O_16_PS [M + 2H]^+^/2; found, 600.65. ^1^H NMR (400 MHz, DMSO-*d*_6_/D_2_O) δ 8.78 (s, 1H), 7.98–7.90 (m, 1H), 7.59–7.39 (m, 6H), 7.32–7.23 (m, 1H), 7.01–6.83 (m, 2H), 5.43–5.22 (m, 1H), 5.08–4.83 (m, 1H), 4.54–4.15 (m, 3H), 4.13–3.83 (m, 5H), 3.80–3.74 (m, 1H), 3.61 (s, 3H), 3.33 (d, *J* = 12.4 Hz, 2H), 3.22–3.18 (m, 3H), 3.11–2.95 (m, 2H), 2.93–2.79 (m, 1H), 2.73–2.57 (m, 3H), 2.46–2.32 (m, 2H), 2.27–2.10 (m, 3H), 2.05–1.92 (m, 3H), 1.92–1.51 (m, 14H).

#### (2-(((5*S*,8*S*,10*aR*)-8-(((*S*)-5-Amino-1-(3-(methylsulfonyl)­phenoxy)-5-oxopentan-2-yl)­carbamoyl)-3-(6-(4-(1-(2,6-dioxopiperidin-3-yl)-3-methyl-2-oxo-2,3-dihydro-1*H*-benzo­[*d*]­imidazol-5-yl)­piperidin-1-yl)-6-oxohexanoyl)-6-oxodecahydropyrrolo­[1,2-*a*]­[1,5]­diazocin-5-yl)­carbamoyl)-1*H*-indole-5-carbonyl)­phosphonic Acid (**33**)

This compound was synthesized by the same procedure as that of **28** as a white solid. UPLC-MS (ESI) *m*/*z*: calcd, 600.2 for C_56_H_67_N_10_O_16_PS [M + 2H]^+^/2; found, 600.3. ^1^H NMR (400 MHz, DMSO-*d*_6_/D_2_O) δ 8.11 (d, *J* = 8.9 Hz, 1H), 7.60–7.39 (m, 6H), 7.29 (d, *J* = 7.7 Hz, 1H), 7.07 (d, *J* = 8.4 Hz, 1H), 7.00 (d, *J* = 8.1 Hz, 1H), 6.94–6.85 (m, 1H), 5.31 (dd, *J* = 12.9, 5.5 Hz, 1H), 4.97 (d, *J* = 30.0 Hz, 1H), 4.55 (d, *J* = 12.7 Hz, 1H), 4.43–4.14 (m, 2H), 4.12–3.83 (m, 6H), 3.31 (s, 3H), 3.20 (d, *J* = 3.0 Hz, 3H), 3.08 (t, *J* = 12.1 Hz, 1H), 2.88 (s, 1H), 2.72 (s, 2H), 2.65 (d, *J* = 14.1 Hz, 3H), 2.44–2.29 (m, 3H), 2.26–2.05 (m, 2H), 2.04–1.92 (m, 3H), 1.89–1.40 (m, 16H).

#### (2-(((5*S*,8*S*,10*aR*)-8-(((*S*)-5-Amino-1-(3-(methylsulfonyl)­phenoxy)-5-oxopentan-2-yl)­carbamoyl)-3-(6-(4-(1-(2,6-dioxopiperidin-3-yl)-3-methyl-2-oxo-2,3-dihydro-1*H*-benzo­[*d*]­imidazol-5-yl)­piperazin-1-yl)-6-oxohexanoyl)-6-oxodecahydropyrrolo­[1,2-*a*]­[1,5]­diazocin-5-yl)­carbamoyl)-1*H*-indole-5-carbonyl)­phosphonic Acid (**34**)

This compound was synthesized by the same procedure as that of **28** as a white solid. UPLC-MS (ESI) *m*/*z*: calcd, 600.72 for C_55_H_66_N_11_O_16_PS [M + 2H]^+^/2; found, 600.2. ^1^H NMR (400 MHz, DMSO-*d*_6_/D_2_O) δ 8.87 (s, 1H), 8.01–7.92 (m, 1H), 7.59–7.52 (m, 2H), 7.51–7.46 (m, 2H), 7.44–7.40 (m, 2H), 7.31–7.26 (m, 1H), 6.96–6.93 (m, 1H), 6.83–6.82 (m, 1H), 6.69–6.61 (m, 0H), 5.38–5.15 (m, 1H), 5.03–4.84 (m, 1H), 4.40–4.16 (m, 2H), 4.12–3.96 (m, 2H), 3.89–3.69 (m, 7H), 3.40–3.36 (m, 3H), 3.31–3.23 (m, 4H), 3.22–3.16 (m, 4H), 3.08–2.97 (m, 1H), 2.87 (d, *J* = 8.4 Hz, 1H), 2.69–2.61 (m, 3H), 2.46–2.31 (m, 5H), 2.24–2.07 (m, 2H), 2.03–1.92 (m, 2H), 1.88–1.77 (m, 2H), 1.73–1.48 (m, 7H).

#### (2-(((5*S*,8*S*,10*aR*)-8-(((*S*)-5-Amino-1-(3-(methylsulfonyl)­phenoxy)-5-oxopentan-2-yl)­carbamoyl)-3-(6-(4-(3-(2,6-dioxopiperidin-3-yl)-1-methyl-1*H*-indazol-7-yl)­piperidin-1-yl)-6-oxohexanoyl)-6-oxodecahydropyrrolo­[1,2-*a*]­[1,5]­diazocin-5-yl)­carbamoyl)-1*H*-indole-5-carbonyl)­phosphonic Acid (**35**)

This compound was synthesized by the same procedure as that of **28** as a white solid. UPLC-MS (ESI) *m*/*z*: calcd, 1182.42 for C_56_H_67_N_10_O_15_PS [M + H]^+^; found, 1182.90. ^1^H NMR (400 MHz, DMSO-*d*_6_/D_2_O) δ 8.78 (d, *J* = 1.8 Hz, 1H), 7.94 (dd, *J* = 8.8, 1.7 Hz, 1H), 7.60–7.44 (m, 4H), 7.41 (d, *J* = 7.5 Hz, 2H), 7.35–7.22 (m, 1H), 7.20 (q, *J* = 7.1, 6.4 Hz, 1H), 7.03 (dq, *J* = 15.1, 7.5 Hz, 1H), 4.95 (dd, *J* = 34.2, 10.2 Hz, 1H), 4.58 (d, *J* = 12.7 Hz, 1H), 4.40–4.27 (m, 2H), 4.26–4.15 (m, 4H), 4.02 (tq, *J* = 13.3, 6.2, 3.2 Hz, 4H), 3.90 (d, *J* = 13.2 Hz, 2H), 3.70–3.52 (m, 1H), 3.42–3.28 (m, 2H), 3.19 (d, *J* = 4.3 Hz, 4H), 2.81–2.54 (m, 4H), 2.46–2.25 (m, 4H), 2.25–2.05 (m, 4H), 2.03–1.43 (m, 15H).

#### (2-(((5*S*,8*S*,10*aR*)-8-(((*S*)-5-Amino-1-(3-(methylsulfonyl)­phenoxy)-5-oxopentan-2-yl)­carbamoyl)-3-(6-(4-(3-(2,6-dioxopiperidin-3-yl)-1-methyl-1*H*-indazol-7-yl)­piperazin-1-yl)-6-oxohexanoyl)-6-oxodecahydropyrrolo­[1,2-*a*]­[1,5]­diazocin-5-yl)­carbamoyl)-1*H*-indole-5-carbonyl)­phosphonic Acid (**36**)

This compound was synthesized by the same procedure as that of **28** as a white solid. UPLC-MS (ESI) *m*/*z*: calcd, 1183.4 for C_55_H_66_N_11_O_15_PS [M + H]^+^; found, 1184.3. ^1^H NMR (400 MHz, DMSO-*d*_6_/D_2_O) δ 8.78 (s, 1H), 7.93 (d, *J* = 8.6 Hz, 1H), 7.57–7.44 (m, 3H), 7.44–7.35 (m, 3H), 7.27 (t, *J* = 9.5 Hz, 1H), 7.06–6.94 (m, 2H), 5.09–4.84 (m, 1H), 4.58–4.40 (m, 2H), 4.38–4.28 (m, 2H), 4.27–4.15 (m, 4H), 4.12–3.85 (m, 4H), 3.43–3.28 (m, 2H), 3.25–3.05 (m, 5H), 2.95–2.81 (m, 1H), 2.75–2.52 (m, 3H), 2.45–2.24 (m, 4H), 2.24–2.06 (m, 4H), 2.03–1.42 (m, 15H).

#### (2-(((5*S*,8*S*,10*aR*)-8-(((*S*)-5-Amino-1-(3-(methylsulfonyl)­phenoxy)-5-oxopentan-2-yl)­carbamoyl)-3-(6-(4-(3-(2,6-dioxopiperidin-3-yl)-1-methyl-1*H*-indazol-6-yl)­piperidin-1-yl)-6-oxohexanoyl)-6-oxodecahydropyrrolo­[1,2-*a*]­[1,5]­diazocin-5-yl)­carbamoyl)-1*H*-indole-5-carbonyl)­phosphonic Acid (**37**)

This compound was synthesized by the same procedure as that of **28** as a white solid. UPLC-MS (ESI) *m*/*z*: calcd, 1182.4 for C_56_H_67_N_10_O_15_PS [M + H]^+^; found, 1183.2. ^1^H NMR (400 MHz, DMSO-*d*_6_/D_2_O) δ 8.78 (s, 1H), 7.99–7.89 (m, 1H), 7.63–7.51 (m, 3H), 7.51–7.36 (m, 4H), 7.33–7.22 (m, 1H), 7.05–6.93 (m, 1H), 5.13–4.82 (m, 1H), 4.57 (d, *J* = 12.7 Hz, 1H), 4.40–4.14 (m, 3H), 4.10–3.84 (m, 8H), 3.42–3.27 (m, 1H), 3.19 (s, 3H), 3.16–3.02 (m, 1H), 2.95–2.78 (m, 1H), 2.73–2.54 (m, 4H), 2.45–2.26 (m, 4H), 2.25–2.06 (m, 4H), 2.04–1.91 (m, 2H), 1.91–1.45 (m, 15H).

#### (2-(((5*S*,8*S*,10*aR*)-8-(((*S*)-5-Amino-1-(3-(methylsulfonyl)­phenoxy)-5-oxopentan-2-yl)­carbamoyl)-3-(6-(4-(3-(2,6-dioxopiperidin-3-yl)-1-methyl-1*H*-indazol-6-yl)­piperazin-1-yl)-6-oxohexanoyl)-6-oxodecahydropyrrolo­[1,2-*a*]­[1,5]­diazocin-5-yl)­carbamoyl)-1*H*-indole-5-carbonyl)­phosphonic Acid (**38**)

This compound was synthesized by the same procedure as that of **28** as a white solid. UPLC-MS (ESI) *m*/*z*: calcd, 1183.4 for C_55_H_66_N_11_O_15_PS [M + H]^+^; found, 1184.3. ^1^H NMR (400 MHz, DMSO-*d*_6_/D_2_O) δ 8.77 (s, 1H), 8.00–7.85 (m, 1H), 7.61–7.50 (m, 3H), 7.50–7.37 (m, 5H), 6.87–6.82 (m, 1H), 5.07–4.81 (m, 1H), 4.41–4.15 (m, 2H), 4.13–3.93 (m, 5H), 3.92–3.82 (m, 6H), 3.45–3.29 (m, 1H), 3.25–3.11 (m, 7H), 2.70–2.56 (m, 4H), 2.47–2.34 (m, 3H), 2.34–2.05 (m, 6H), 2.02–1.50 (m, 12H).

#### (2-(((5*S*,8*S*,10*aR*)-8-(((*S*)-5-Amino-1-(3-(methylsulfonyl)­phenoxy)-5-oxopentan-2-yl)­carbamoyl)-3-(6-(4-(4-(2,6-dioxopiperidin-3-yl)-3,5-difluorophenyl)­piperazin-1-yl)-6-oxohexanoyl)-6-oxodecahydropyrrolo­[1,2-*a*]­[1,5]­diazocin-5-yl)­carbamoyl)-1*H*-indole-5-carbonyl)­phosphonic Acid (**39**)

This compound was synthesized by the same procedure as that of **28** as a white solid. UPLC-MS (ESI) *m*/*z*: calcd, 1165.4 for C_53_H_62_F_2_N_9_O_15_PS [M + H]^+^; found, 1166.4. ^1^H NMR (400 MHz, DMSO-*d*_6_/D_2_O) δ 8.78 (s, 1H), 7.97–7.90 (m, 1H), 7.61–7.35 (m, 5H), 7.35–7.20 (m, 1H), 6.64–6.57 (m, 2H), 5.05–4.86 (m, 1H), 4.39–4.28 (m, 2H), 4.25–4.16 (m, 3H), 4.14–3.95 (m, 5H), 3.27–3.11 (m, 7H), 2.90–2.86 (m, 4H), 2.80–2.65 (m, 4H), 2.43–2.30 (m, 4H), 2.26–2.04 (m, 4H), 1.99–1.41 (m, 10H).

#### (2-(((5*S*,8*S*,10*aR*)-8-(((*S*)-5-Amino-1-(3-(methylsulfonyl)­phenoxy)-5-oxopentan-2-yl)­carbamoyl)-3-(6-(4-(2-((*S*)-2,6-dioxopiperidin-3-yl)-1-oxoisoindolin-5-yl)­piperazin-1-yl)-6-oxohexanoyl)-6-oxodecahydropyrrolo­[1,2-*a*]­[1,5]­diazocin-5-yl)­carbamoyl)-1*H*-indole-5-carbonyl)­phosphonic Acid (**40**)

This compound was synthesized by the same procedure as that of **28** as a white solid. UPLC-MS (ESI) *m*/*z*: calcd, 1184.4 for C_55_H_65_N_10_O_16_PS [M + H]^+^; found, 1185.8. ^1^H NMR (400 MHz, DMSO-*d*_6_/D_2_O) δ 8.80 (s, 1H), 7.94 (s, 1H), 7.67–7.38 (m, 6H), 7.35–7.22 (m, 1H), 7.10–6.94 (m, 2H), 5.16–4.81 (m, 1H), 4.41–4.15 (m, 5H), 4.12–3.91 (m, 6H), 3.61–3.15 (m, 12H), 2.99–2.79 (m, 1H), 2.77–2.54 (m, 4H), 2.43–2.30 (m, 2H), 2.26–2.09 (m, 3H), 2.02–1.89 (m, 1H), 1.88–1.78 (m, 2H), 1.68 (d, *J* = 9.6 Hz, 2H), 1.63–1.53 (m, 4H).

### HiBiT Assay

STAT3-HiBiT HeLa cell line was purchased from Promega. Cells seeded in 384-well white plates (Corning) were incubated with serially diluted compounds for 24 h at 37 °C with 5% CO_2_. At the end of treatment, Nano-Glo HiBiT Lytic Detection reagents (Promega) were added to the wells, and luminescence was acquired on the TECAN SPARK plate reader. Untreated cells were used as a control. Data points were fit with a four-parameter equation to generate a concentration–response curve. DC_50_ values were calculated using a nonlinear regression analysis to obtain the mean ± standard deviation (SD) from triplicate.

### Western Blot Analysis

Western blotting was performed as described previously.^22^ Cells were lysed in cell lysis buffer (Cell Signaling Technology, no. 9803), separated by SDS-PAGE NuPAGE gel (Thermo Fisher Scientific), and transferred to a polyvinylidene difluoride (PVDF) membrane (Millipore). PVDF membranes were first blocked for 1 h using 5% blotting-grade blocker (no. 1706404, Bio-Rad) in Tris-buffered saline with Tween 20 (TBST, Pierce) and then incubated with the primary and secondary antibodies. The secondary antibodies used are IRDye 800CW goat anti-mouse and IRDye 680RD goat anti-rabbit secondary antibodies (LI-COR Biosciences). Membranes were scanned, and bands were quantified with the Odyssey CLx Imaging System (LI-COR Biosciences).

### Cell Growth Assay

Cell viability analysis was performed as described previously. Cells grown in 384-well white plates (Corning Costar) were incubated with serially diluted compounds for 4 days. Cell viability was determined using the Cell Titer-Glo luminescent cell viability assay (Promega) following the manufacturer’s instructions.

### Global Proteomics Analysis

PBMCs cells were treated with 10 μM of SD-965 for 8 h. Global proteomic analysis was performed using the same procedure as described in detail in our previous study.^33^

### Pharmacodynamic and Efficacy Studies in Mice

All *in vivo* studies were performed under animal protocols (PRO00009463 and PRO00011174) approved by the Institutional Animal Care and Use Committee (IACUC) of the University of Michigan, in accordance with the recommendations in the Guide for the Care and Use of Laboratory Animals of the National Institutes of Health.

For PD and efficacy studies in tumor-bearing mice, CB.17 SCID mice were injected subcutaneously with 3 × 10^6^ MOLM-16 cells or 10 × 10^6^ SU-DHL-1 cells in 5 mg/mL Matrigel (Corning) for tumor growth. When the tumors reached an average volume of 100–200 mm,^3^ mice were randomly assigned to different experimental groups for treatment. Drugs or vehicle control (10% PEG400 + 90% phosphate-buffered saline (PBS)) were given at the indicated dose schedules in the figures. Tumor sizes and animal weights were measured 2 times per week. Tumor volumes were calculated as tumor volume (mm^3^) = (length × width^2^)/2. For PD studies, mice were euthanized at the indicated time points in the figures, and tumor tissues or mouse organs were harvested for Western blotting analysis or analysis of drug concentrations in tumor or native tissues.

### Pharmacokinetic (PK) Study in Mice and Microsomal Metabolic and Plasma Stability Studies

All of these studies were performed in Shanghai Medicilon Inc., Shanghai, 201200, China. The detailed protocols for the PK studies and microsomal and plasma stability studies were the same as described previously.^37^ The formulation used in the PK study for SD-965 was 5% DMSO, 10% Solutol, and 85% saline.

## Supplementary Material





## Notes

HiBit assay graphs for degradation induced by SD-965, **25**, **31**, and **32**, cell growth inhibitory activity graphs of SD-965, **25**, **31**, and **32** in two cancer cell lines, UPLC spectra for purity of synthesized degraders, ^1^H NMR spectra for SD-965, **25**, **31**, and **32** and ^13^C NMR data for SD-965, representation of a previously reported cocrystal structure of SD-36 in complex with STAT3 (PDF)
Molecular string file for all of the final degrader compounds (CSV)

## Abbreviations Used

AUC: area-under-the-curve
Cl: clearance
*C*_max_: maximum drug concentration
DC_50_: the concentration needed to reduce the protein by 50%
*D*_max_: maximal degradation
IV: intravenous administration
PROTAC: proteolysis-targeting chimera
PK: pharmacokinetic
PD: pharmacodynamic
*T*_1/2_: elimination half-life
*V*_ss_: volume of distribution at steady state
