# Supplementary material for: Discovery of SD-965 as a Potent, Selective, and Efficacious STAT3 PROTAC Degrader
Source: J Med Chem. 2026 Mar 23;69(7):8364–87. doi: 10.1021/acs.jmedchem.5c03767 (PMC13071879; doi:10.1021/acs.jmedchem.5c03767)

Supporting Information

Discovery of SD-965 as a Potent, Selective and Efficacious STAT3 PROTAC Degrader

Dimin Wu,[†,‡] Haibin Zhou,[†,‡,*] Longchuan Bai,[†,‡,*] Ranjan Kumar Acharyya,[†] Hoda Metwally,[†] Donna McEachern,[†] Mi Wang,[†] Jelena Tošović,[†] Rohan Kalyan Rej,[†] Meilin Wang[#], Bo Wen[#], Duxin Sun[#], Shaomeng Wang[†,¥,§,±,*]

[†]Department of Internal Medicine, Division of Hematology/Oncology, University of Michigan, Ann Arbor, Michigan 48109, United States

[¥]Department of Pharmacology, University of Michigan, Ann Arbor, Michigan 48109, United States

[§]Department of Medicinal Chemistry, College of Pharmacy, University of Michigan, Ann Arbor, Michigan 48109, United States

[#]Department of Pharmaceutical Sciences, College of Pharmacy, University of Michigan, Ann Arbor, Michigan 48109, United States

[±]The Rogel Cancer Center, University of Michigan, Ann Arbor, Michigan 48109, United States

[‡]These authors contributed equally.

[*]Corresponding authors: Haibin Zhou (haibinz@med.umich.edu), Longchuan Bai (lbai@med.umich.edu), Shaomeng Wang (shaomeng@med.umich.edu)

Contents

1. HiBit assay analysis graphs for degradation induced by **SD-965**, **25**, **31**, **32** 3

2. Cell growth inhibitory activities graphs of **SD-965**, **25**, **31**, **32** in two cancer cell lines. 4

3. UPLC-MS spectra of STAT3 degraders 5

4. NMR spectra of STAT3 degraders 34

5. Co-crystal structure of SD-36 with STAT3 39

1. **Figure S1**. HiBit assay analysis graphs for degradation induced by **SD-965**, **25**, **31**, **32**.

STAT3-HiBiT

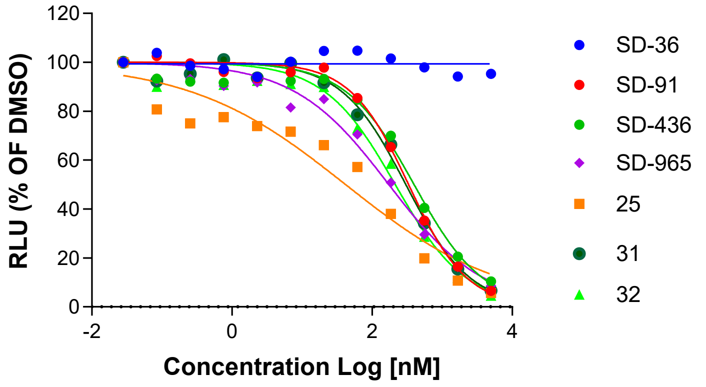

STAT3-HiBiT

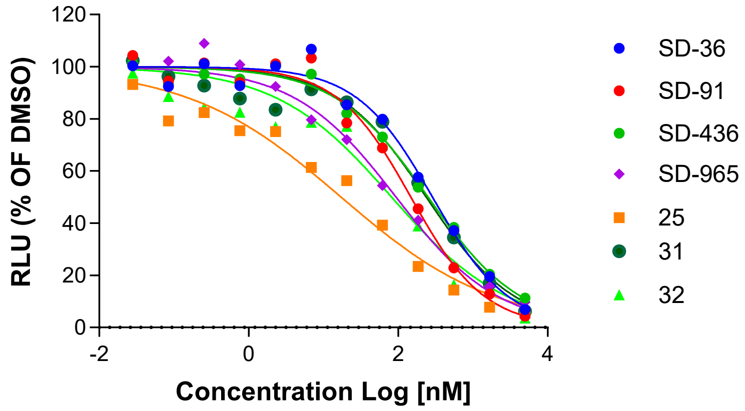

STAT3-HiBiT

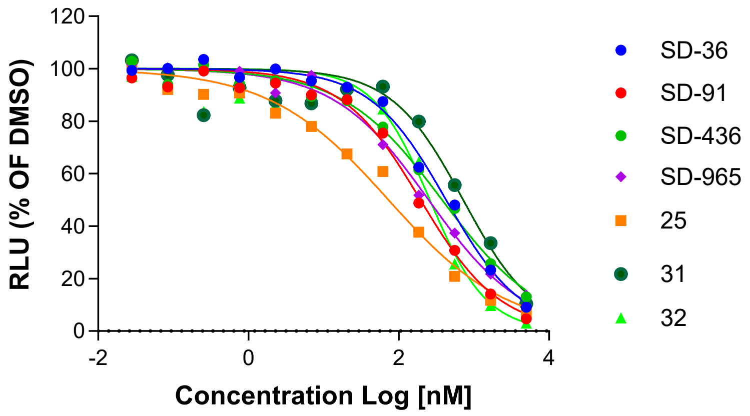

2. **Figure S2**. Cell growth inhibitory activities graphs of **SD-965**, **25**, **31**, **32** in two cancer cell lines.

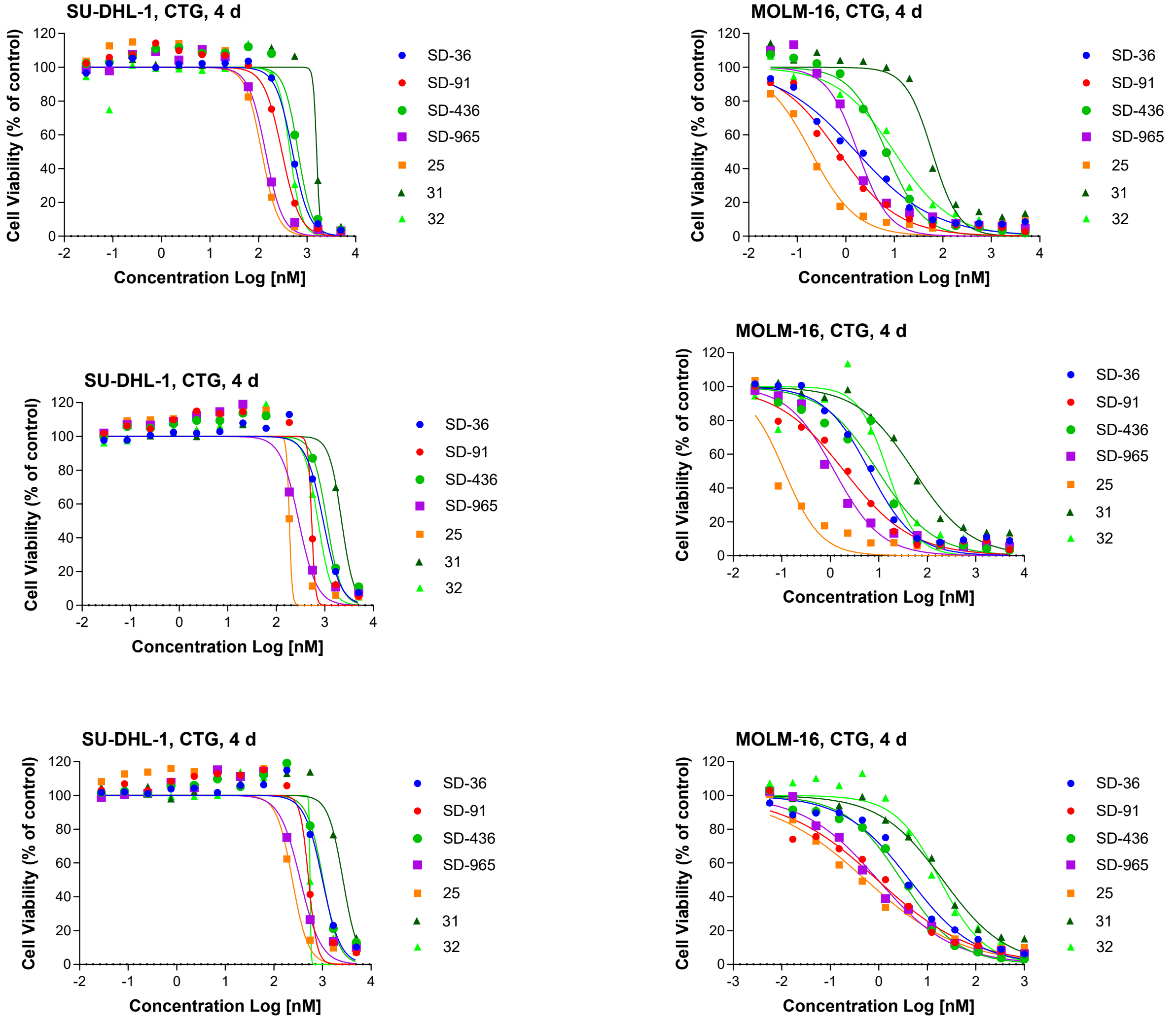

3. **Figure S3**. UPLC-MS spectra of STAT3 degraders

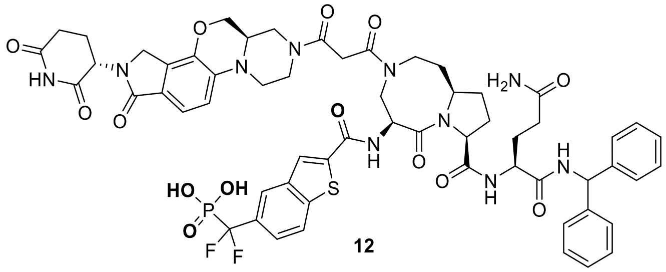

Chemical Formula: $C_{59}H_{61}F_2N_{10}O_{14}PS$
Molecular Weight: 1235.22

SAMPLE INFORMATION

Sample Name:	DW1488-4-2	Acquired By:	System
Sample Type:	Unknown	Date Acquired:	7/16/2025 4:32:41 PM EDT
Vial:	1:E,3	Acq. Method Set:	10to100% Bin 10
Injection #:	1	Date Processed:	min_Delay2min 7/17/2025 2:27:20 PM EDT
Injection Volume:	5.00 ul	Processing Method:	Bruce
Run Time:	10.0 Minutes	Channel Name:	254.0nm
Sample Set Name:	0	Proc. Chnl. Descr.:	PDA Spectrum (210-500)nm

Auto-Scaled Chromatogram

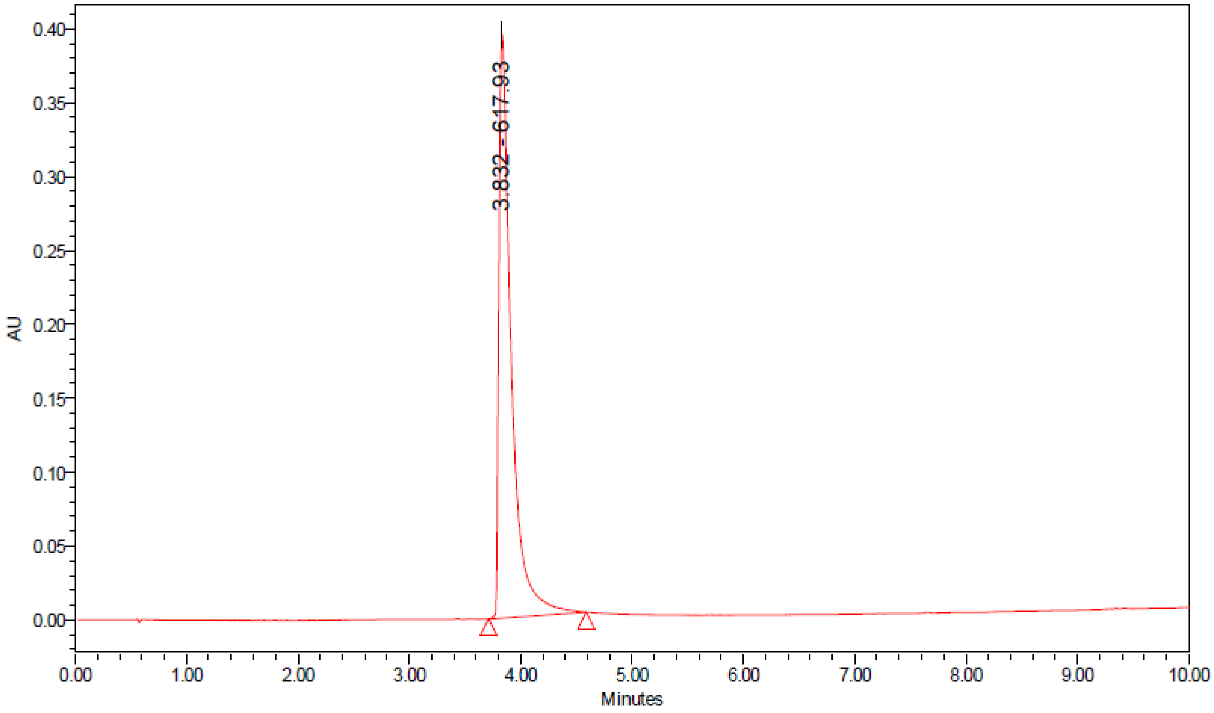

Peak Results

	RT	Area	Height	% Area	Base Peak (m/z)
1	3.832	3077917	394985	100.00	617.93

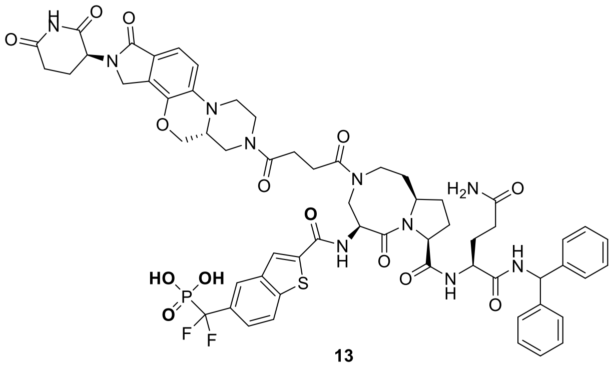

13

Chemical Formula: $C_{60}H_{63}F_2N_{10}O_{14}PS$
Molecular Weight: 1249.25

S A M P L E   I N F O R M A T I O N

Sample Name:	DW911-3-2	Acquired By:	System
Sample Type:	Unknown	Date Acquired:	1/20/2026 9:11:33 AM EST
Vial:	1:C,1	Acq. Method Set:	10to100% Bin 10
Injection #:	1	Date Processed:	m/z_Delay4min 1/20/2026 5:47:48 PM EST
Injection Volume:	5.00 ul	Processing Method:	Bruce
Run Time:	10.0 Minutes	Channel Name:	254.0nm
Sample Set Name:	1	Proc. Chnl. Descr.:	PDA Spectrum (210-500)nm

Auto-Scaled Chromatogram

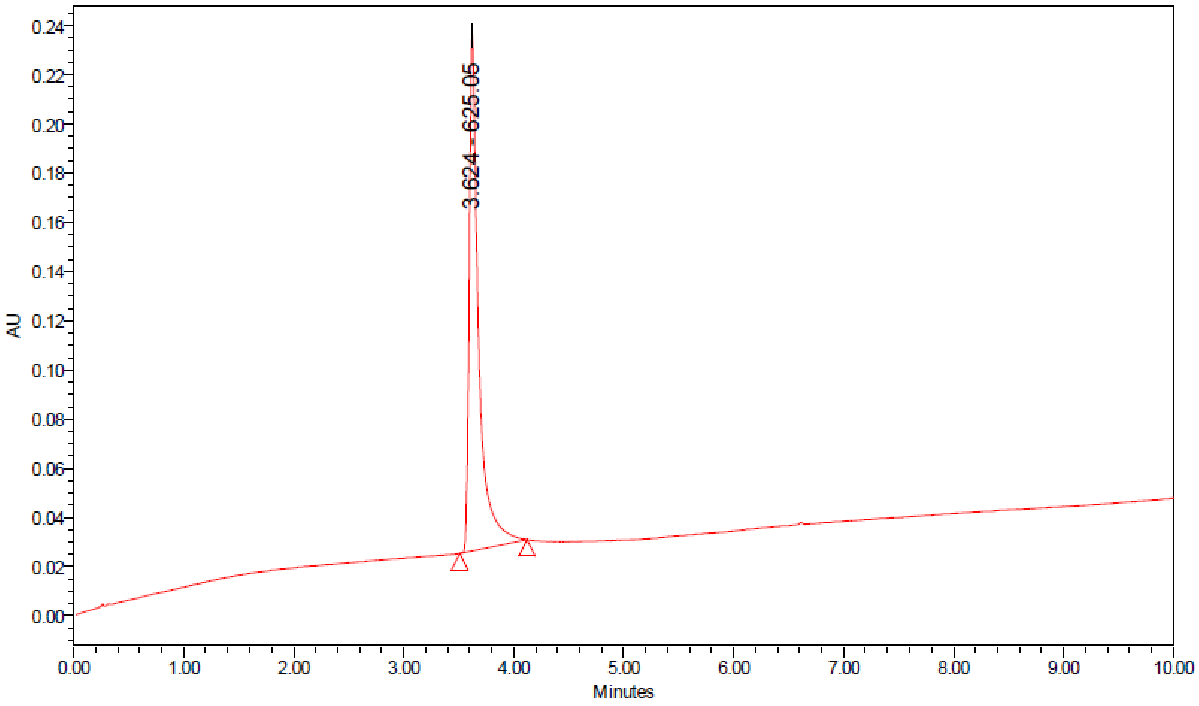

Peak Results

	RT	Area	Height	% Area	Base Peak (m/z)
1	3.624	1250928	209787	100.00	625.05

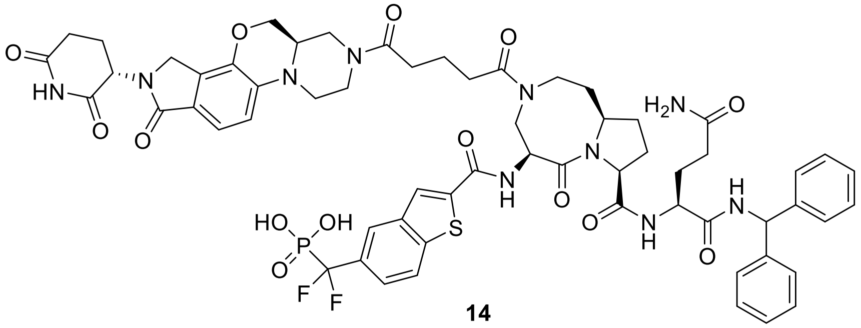

14

Chemical Formula: $C_{61}H_{65}F_2N_{10}O_{14}PS$
Molecular Weight: 1263.28

SAMPLE INFORMATION

Sample Name:	DW908-3-2	Acquired By:	System
Sample Type:	Unknown	Date Acquired:	1/20/2026 9:58:35 AM EST
Vial:	1:C,4	Acq. Method Set:	10to100% Bin 10
Injection #:	1	Date Processed:	[illegible]:13 PM EST
Injection Volume:	5.00 ul	Processing Method:	Bruce
Run Time:	10.0 Minutes	Channel Name:	254.0nm@7
Sample Set Name:	1	Proc. Chnl. Descr.:	PDA Spectrum (210-500)nm

Auto-Scaled Chromatogram

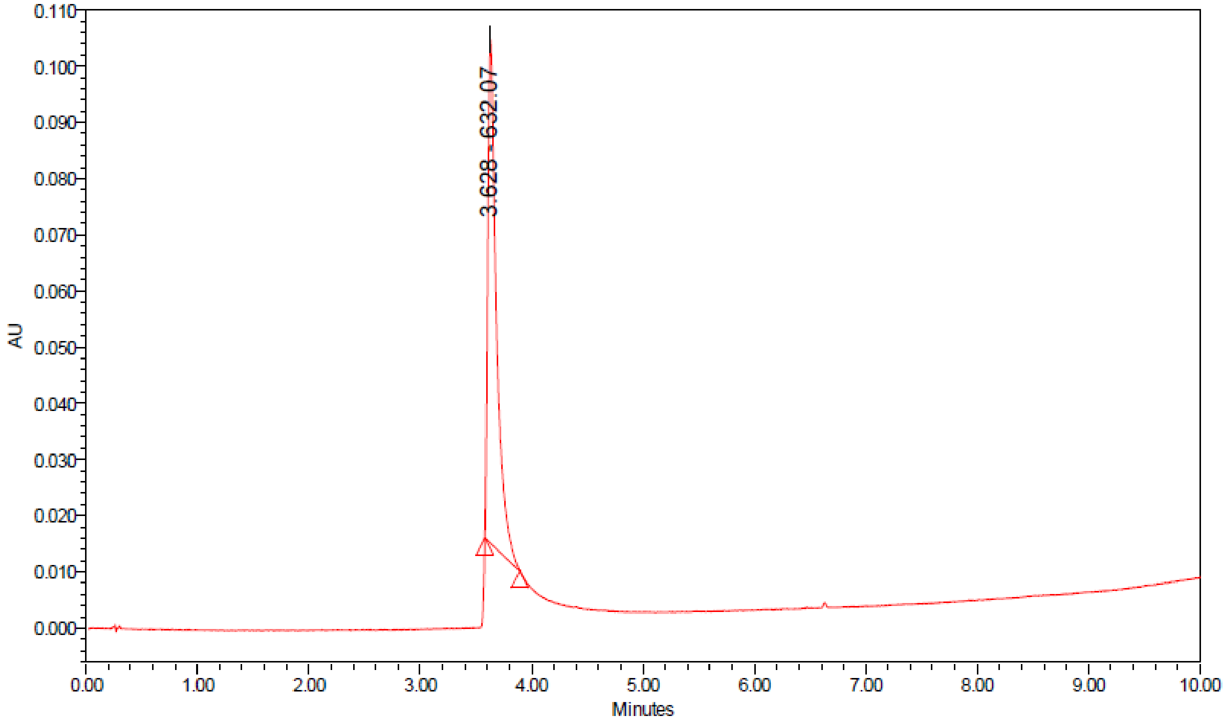

Peak Results

	RT	Area	Height	% Area	Base Peak (m/z)
1	3.628	485649	89690	100.00	632.07

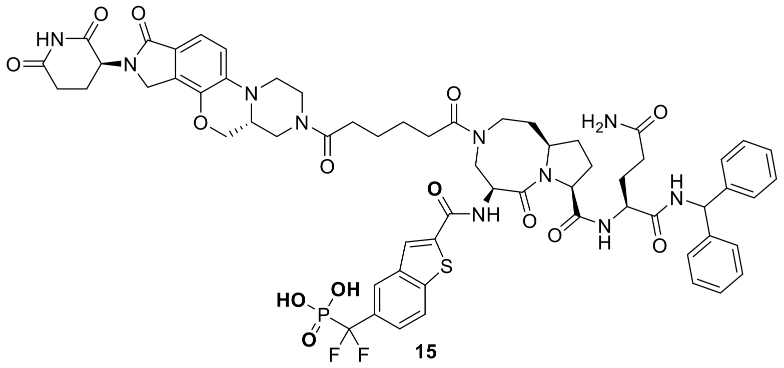

15

Chemical Formula: $C_{62}H_{67}F_2N_{10}O_{14}PS$
Molecular Weight: 1277.30

SAMPLE INFORMATION

Sample Name:	DW909-5-1	Acquired By:	System
Sample Type:	Unknown	Date Acquired:	1/19/2026 5:19:05 PM EST
Vial:	1:F,6	Acq. Method Set:	10to100% Bin 10
Injection #:	1	Date Processed:	1/19/2026 5:21:49 PM EST
Injection Volume:	5.00 ul	Processing Method:	Bruce
Run Time:	10.0 Minutes	Channel Name:	254.0nm
Sample Set Name:	1	Proc. Chnl. Descr.:	PDA Spectrum (210-500)nm

Auto-Scaled Chromatogram

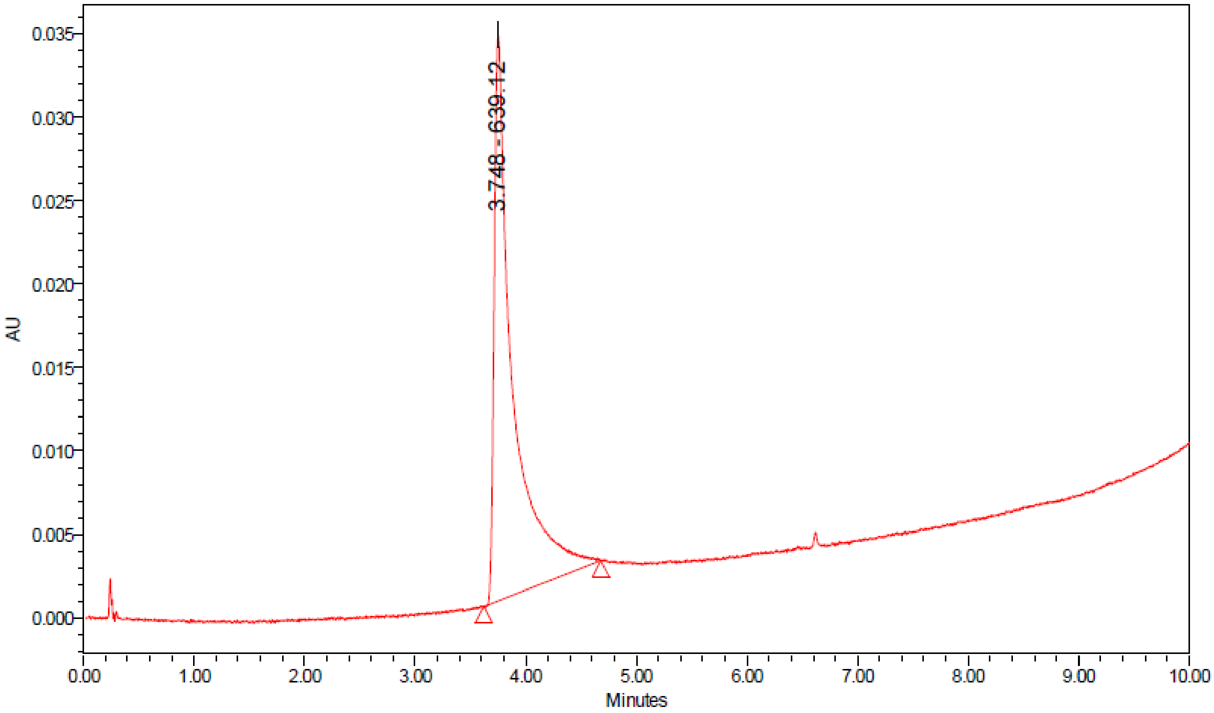

Peak Results

	RT	Area	Height	% Area	Base Peak (m/z)
1	3.748	401053	33963	100.00	639.12

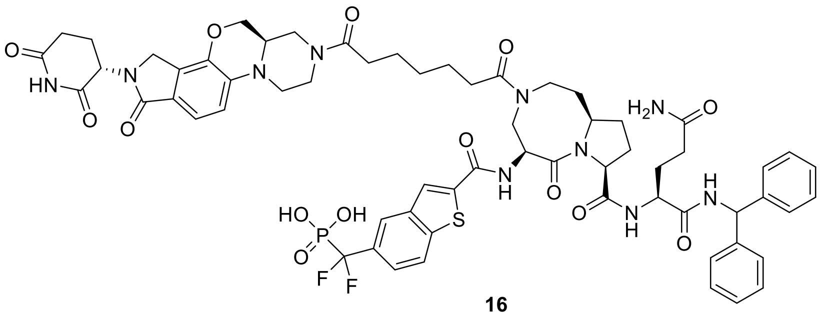

16

Chemical Formula: $C_{63}H_{69}F_2N_{10}O_{14}PS$

Molecular Weight: 1291.33

SAMPLE INFORMATION

Sample Name:	DW921-2-3	Acquired By:	System
Sample Type:	Unknown	Date Acquired:	1/20/2026 10:45:37 AM EST
Vial:	1:C,7	Acq. Method Set:	10to100% Bin 10
Injection #:	1	Date Processed:	[illegible]47:41 PM EST
Injection Volume:	5.00 ul	Processing Method:	Bruce
Run Time:	10.0 Minutes	Channel Name:	254.0nm
Sample Set Name:	1	Proc. Chnl. Descr.:	PDA Spectrum (210-500)nm

Auto-Scaled Chromatogram

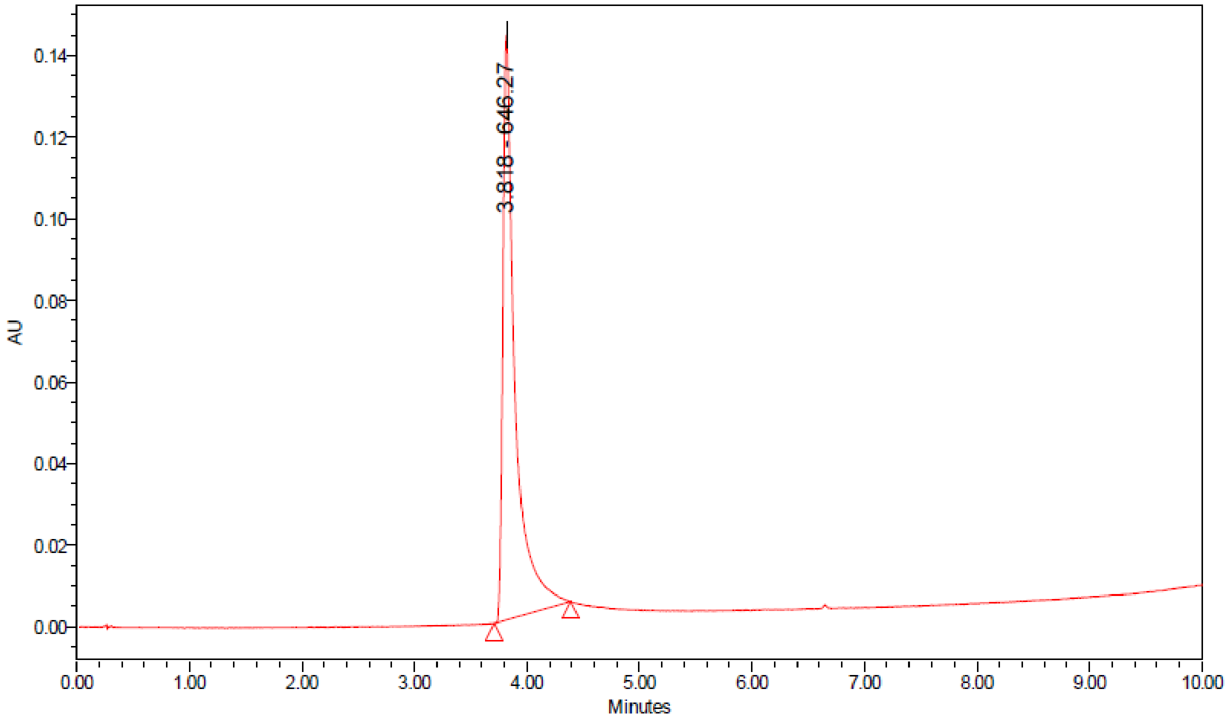

Peak Results

	RT	Area	Height	% Area	Base Peak (m/z)
1	3.818	1090368	143357	100.00	646.27

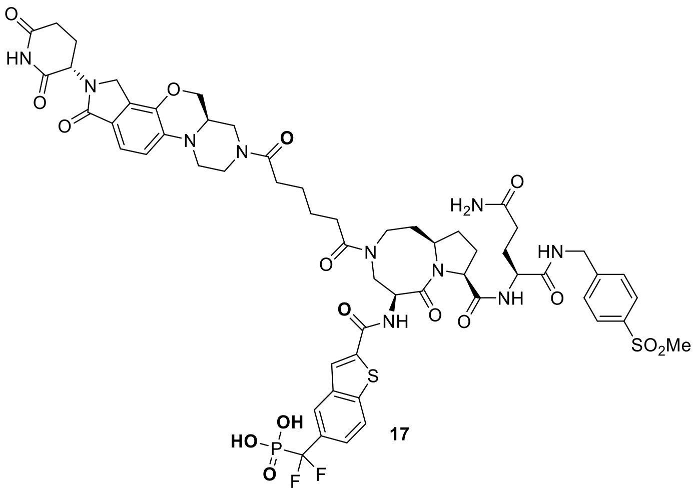

Chemical Formula: $C_{57}H_{65}F_2N_{10}O_{16}PS_2$
Molecular Weight: 1279.29

SAMPLE INFORMATION

Sample Name:	DW1388-8	Acquired By:	System
Sample Type:	Unknown	Sample Set Name:	1
Vial:	1:C,1	Acq. Method Set:	10to100Bin10min_5minDelay
Injection #:	1	Processing Method:	Default
Injection Volume:	5.00 ul	Channel Name:	254.0nm
Run Time:	10.0 Minutes	Proc. Chnl. Descr.:	PDA Spectrum PDA 254.0 nm
Date Acquired:	5/26/2025 10:59:54 AM EDT		
Date Processed:	5/26/2025 9:33:23 PM EDT		

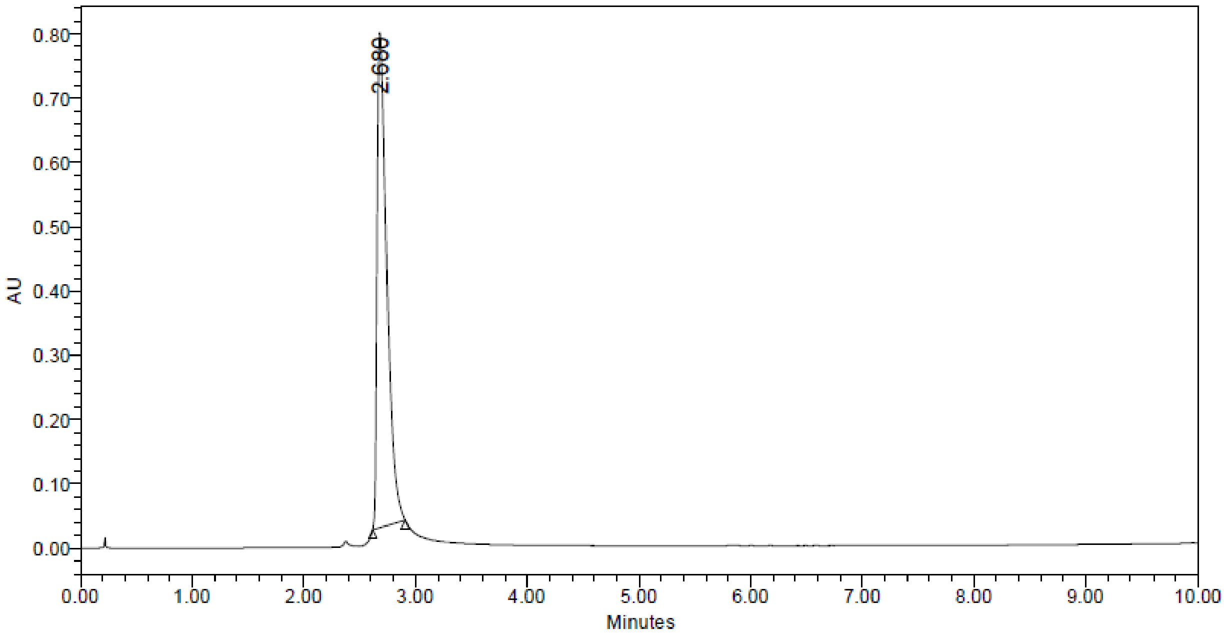

	RT	Area	% Area	Height
1	2.680	4642470	100.00	769384

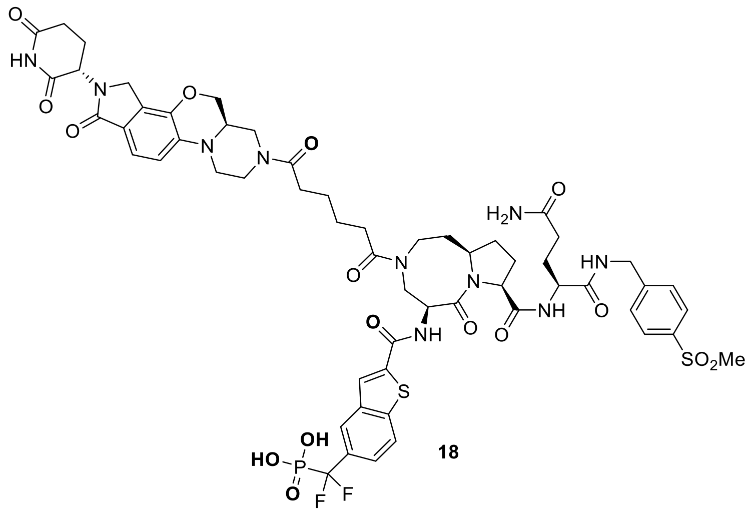

18

Chemical Formula: $C_{57}H_{65}F_2N_{10}O_{16}PS_2$
Molecular Weight: 1279.29

SAMPLE INFORMATION

Sample Name:	DW1984	Acquired By:	System
Sample Type:	Unknown	Date Acquired:	4/26/2025 3:47:47 PM EDT
Vial:	1:C,2	Acq. Method Set:	10to100% Bin 10
Injection #:	1	Date Processed:	4/27/2025 9:31:50 PM EDT
Injection Volume:	5.00 ul	Processing Method:	Bruce
Run Time:	10.0 Minutes	Channel Name:	254.0nm
Sample Set Name:	0	Proc. Chnl. Descr.:	PDA Spectrum (210-500)nm

Auto-Scaled Chromatogram

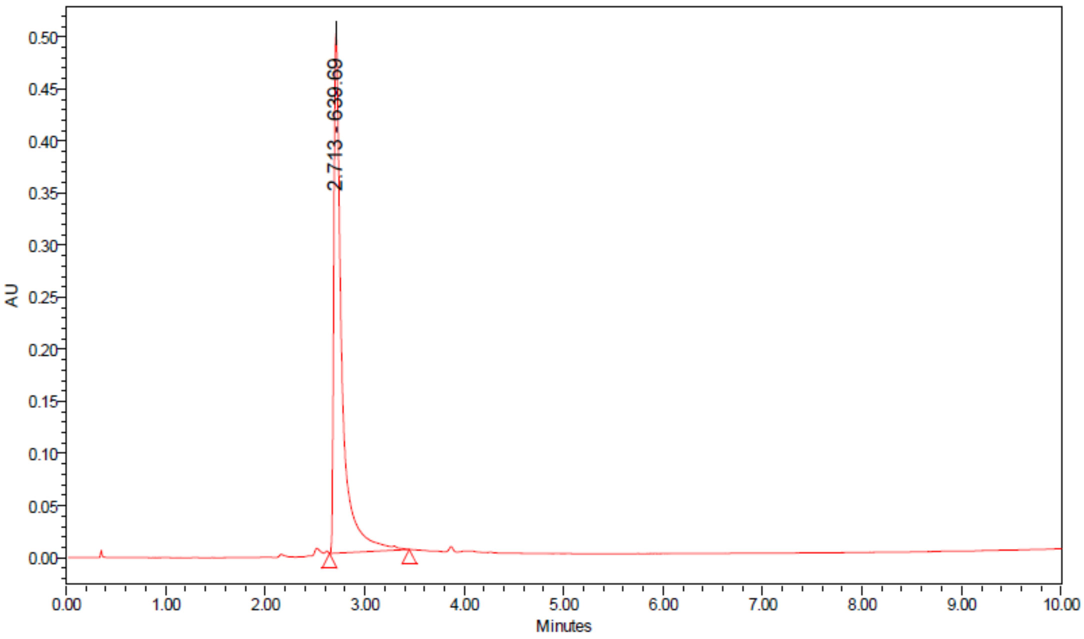

Peak Results

	RT	Area	Height	% Area	Base Peak (m/z)
1	2.713	2793357	499037	100.00	639.69

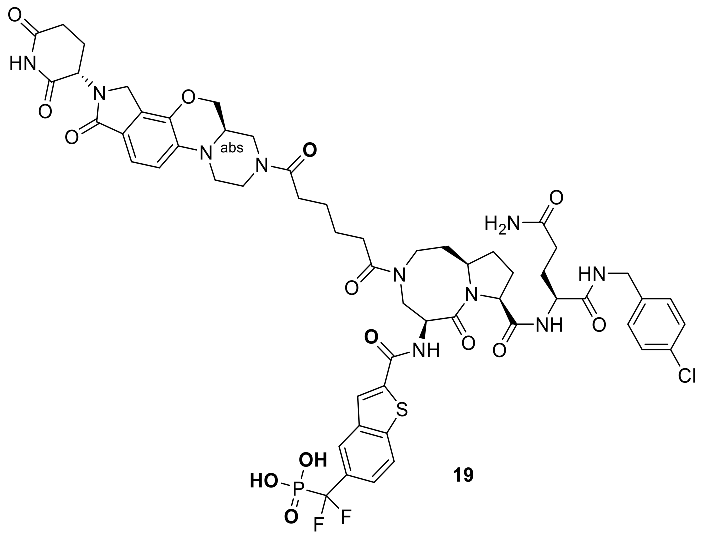

19

Chemical Formula: $C_{56}H_{62}ClF_2N_{10}O_{14}PS$
Molecular Weight: 1235.65

SAMPLE INFORMATION

Sample Name:	DW1385-2-2	Acquired By:	System
Sample Type:	Unknown	Date Acquired:	4/25/2025 4:19:52 PM EDT
Vial:	1:D,4	Acq. Method Set:	10to100% Bin 10
Injection #:	1	Date Processed:	4/25/2025 4:36:42 PM EDT
Injection Volume:	5.00 ul	Processing Method:	Bruce
Run Time:	10.0 Minutes	Channel Name:	254.0nm
Sample Set Name:	0	Proc. Chnl. Descr.:	PDA Spectrum (210-500)nm

Auto-Scaled Chromatogram

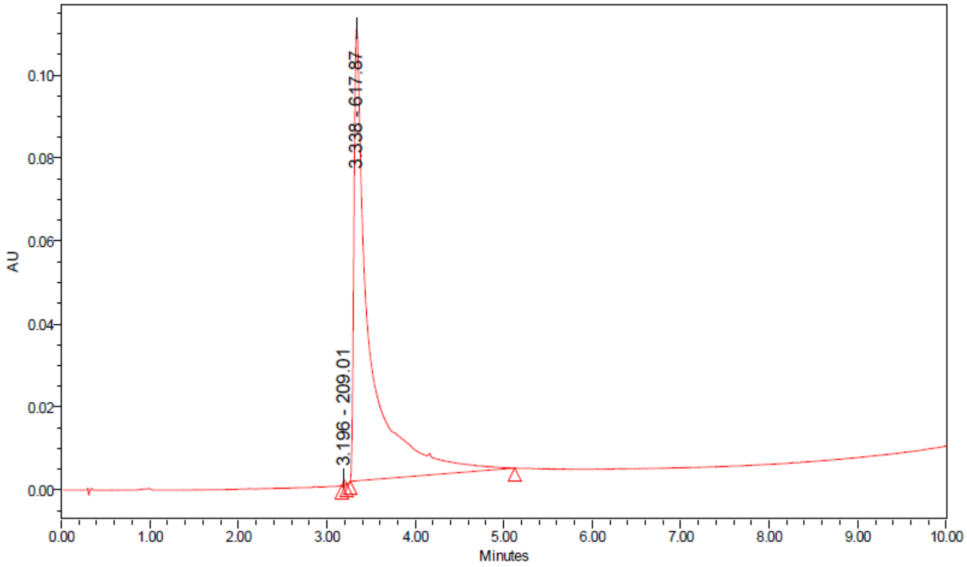

Peak Results

	RT	Area	Height	% Area	Base Peak (m/z)
1	3.196	2056	1268	0.15	209.01
2	3.338	1341046	109053	99.85	617.87

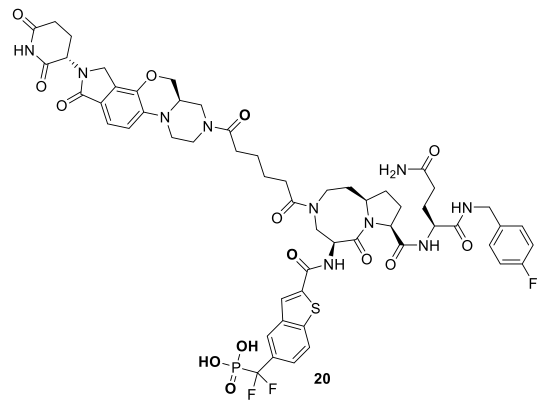

20

Chemical Formula: $C_{56}H_{62}F_{3}N_{10}O_{14}PS$
Molecular Weight: 1219.20

SAMPLE INFORMATION

Sample Name:	dw1386-3-3	Acquired By:	System
Sample Type:	Unknown	Date Acquired:	1/21/2026 4:03:33 PM EST
Vial:	1:B,8	Acq. Method Set:	10to100% Bin 10
Injection #:	1	Date Processed:	m/z_Delay5min 1/21/2026 5:0[illegible]:36 PM EST
Injection Volume:	5.00 ul	Processing Method:	Bruce
Run Time:	10.0 Minutes	Channel Name:	254.0nm
Sample Set Name:	0	Proc. Chnl. Descr.:	PDA Spectrum (210-500)nm

Auto-Scaled Chromatogram

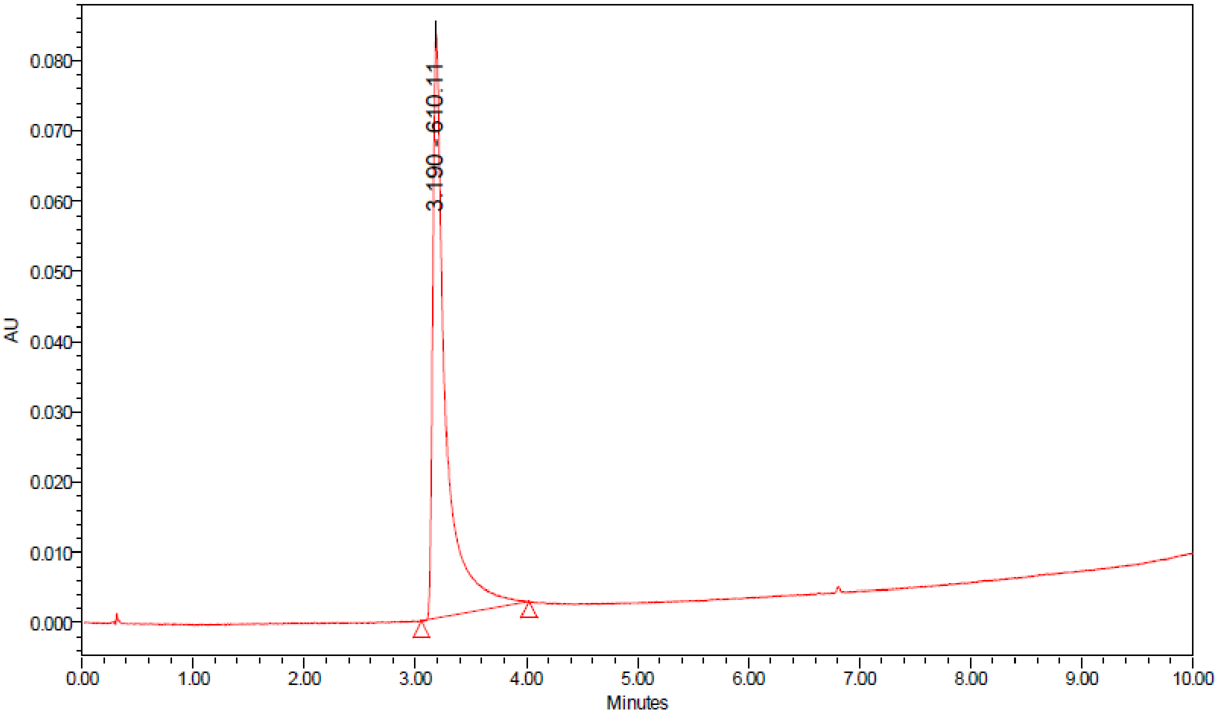

Peak Results

	RT	Area	Height	% Area	Base Peak (m/z)
1	3.190	669212	83136	100.00	610.11

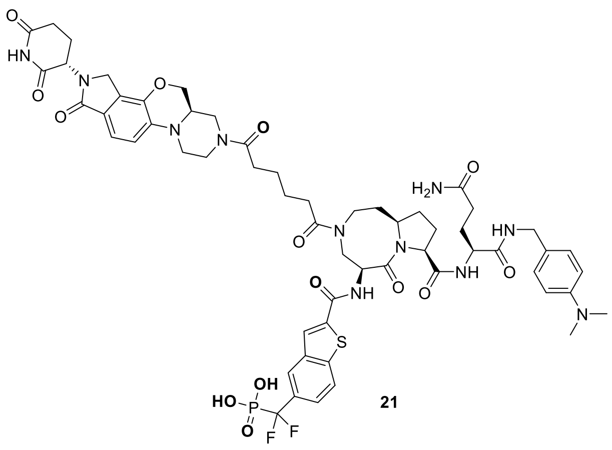

21

Chemical Formula: $C_{58}H_{68}F_2N_{11}O_{14}PS$
Molecular Weight: 1244.28

SAMPLE INFORMATION

Sample Name:	dw1387-0	Acquired By:	System
Sample Type:	Unknown	Date Acquired:	1/21/2026 4:34:51 PM EST
Vial:	1:C,2	Acq. Method Set:	10to100% Bin 10
Injection #:	1	Date Processed:	1/21/2026 5:06:03 PM EST
Injection Volume:	5.00 ul	Processing Method:	Bruce
Run Time:	10.0 Minutes	Channel Name:	254.0nm
Sample Set Name:	0	Proc. Chnl. Descr.:	PDA Spectrum (210-500)nm

Auto-Scaled Chromatogram

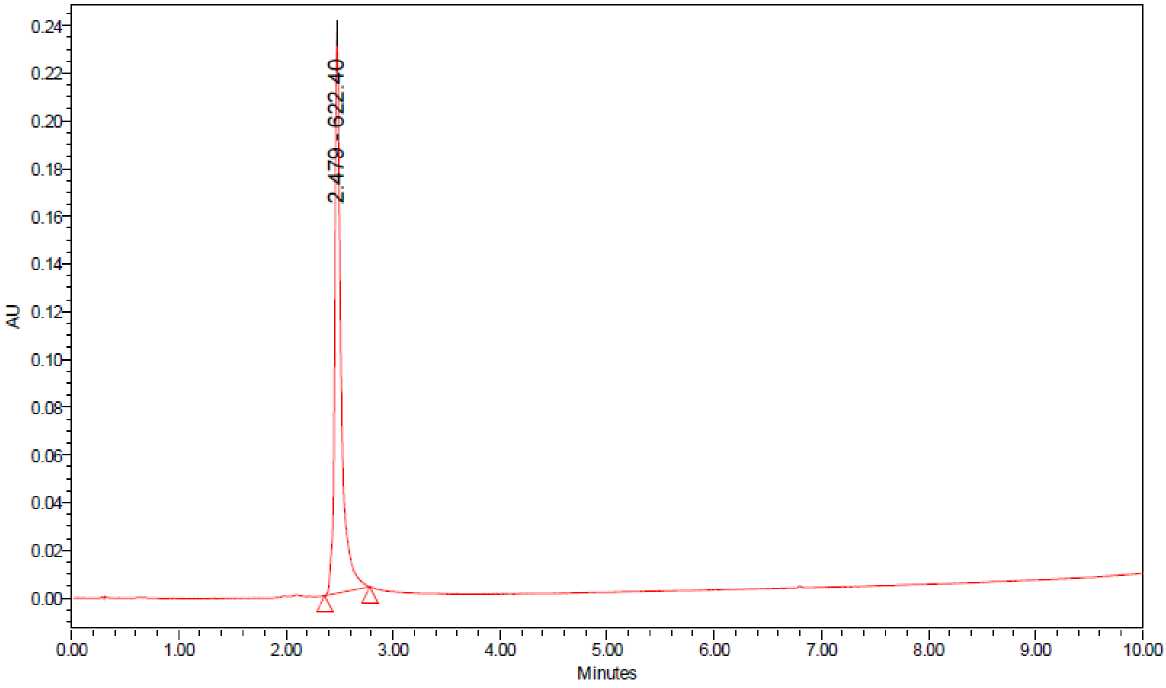

Peak Results

	RT	Area	Height	% Area	Base Peak (m/z)
1	2.479	987964	234777	100.00	622.40

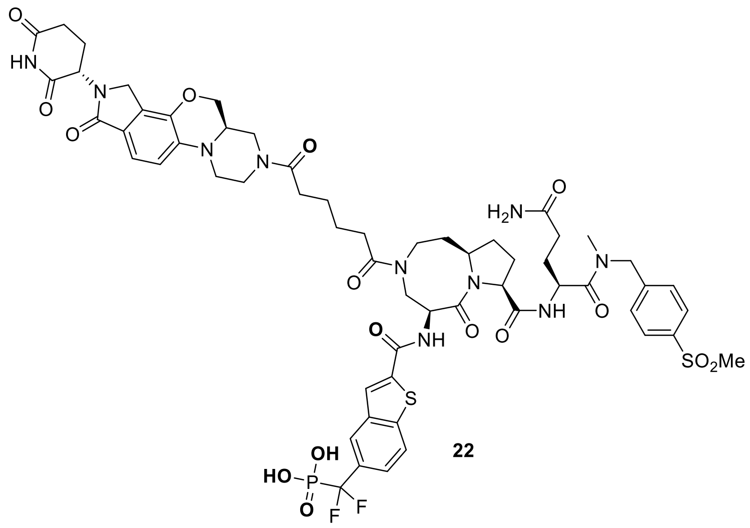

22

Chemical Formula: $C_{58}H_{67}F_{2}N_{10}O_{16}PS_{2}$
Molecular Weight: 1293.32

SAMPLE INFORMATION

Sample Name:	DW1525-8	Acquired By:	System
Sample Type:	Unknown	Date Acquired:	8/21/2025 10:39:51 AM EDT
Vial:	1:C,2	Acq. Method Set:	10to100% Bin 10
Injection #:	1	Date Processed:	m/z_Display_min 8/21/2025 [illegible]:14:54 AM EDT
Injection Volume:	8.00 ul	Processing Method:	Bruce
Run Time:	10.0 Minutes	Channel Name:	254.0nm
Sample Set Name:	1	Proc. Chnl. Descr.:	PDA Spectrum (210-500)nm

Auto-Scaled Chromatogram

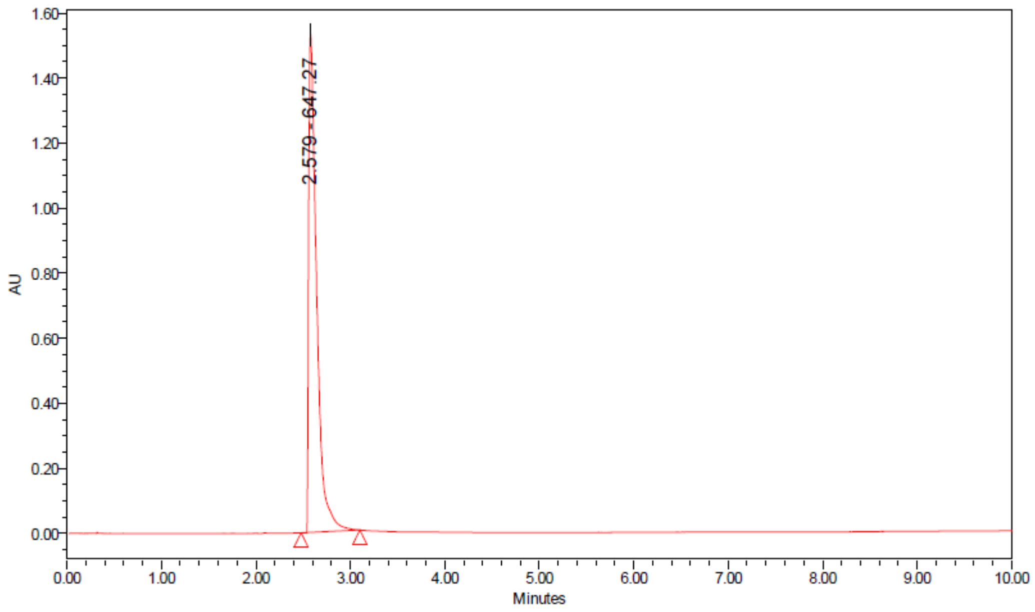

Peak Results

	RT	Area	Height	% Area	Base Peak (m/z)
1	2.579	9005338	1530166	100.00	647.27

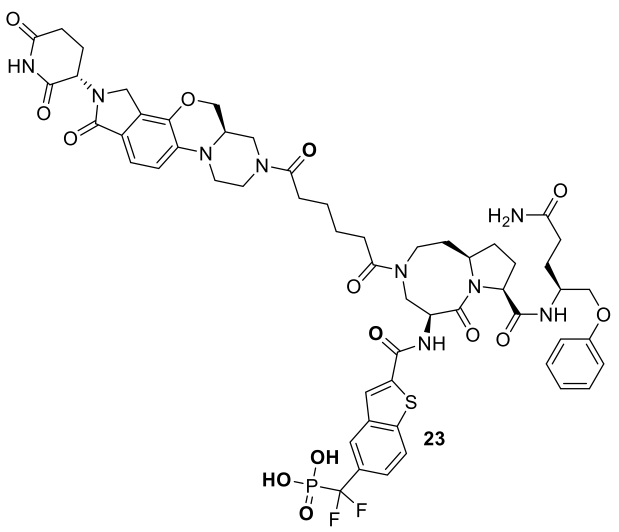

23

Chemical Formula: $C_{55}H_{62}F_2N_9O_{14}PS$
Molecular Weight: 1174.18

SAMPLE INFORMATION

Sample Name:	DW1282-3-2	Acquired By:	System
Sample Type:	Unknown	Sample Set Name:	YQY
Vial:	1:A,7	Acq. Method Set:	10to100Bin10min_5minDelay
Injection #:	1	Processing Method:	Default
Injection Volume:	5.00 ul	Channel Name:	254.0nm
Run Time:	10.0 Minutes	Proc. Chnl. Descr.:	PDA Spectrum PDA 254.0 nm
Date Acquired:	4/23/2025 5:40:14 PM EDT		
Date Processed:	4/23/2025 11:03:16 PM EDT		

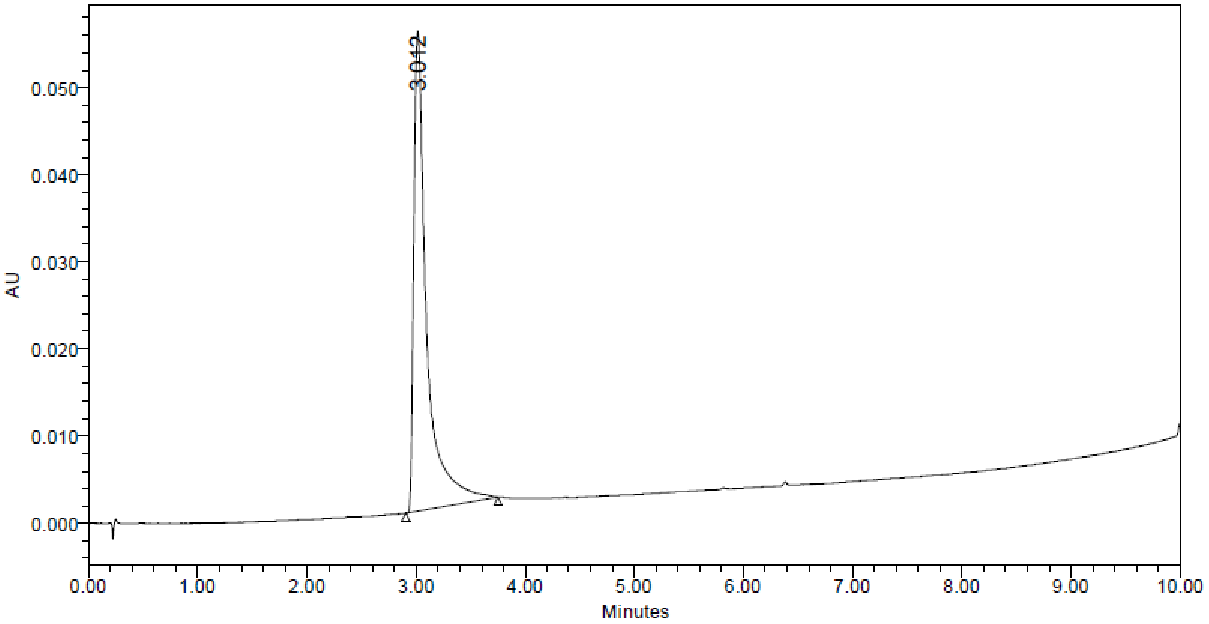

	RT	Area	% Area	Height
1	3.012	454991	100.00	55106

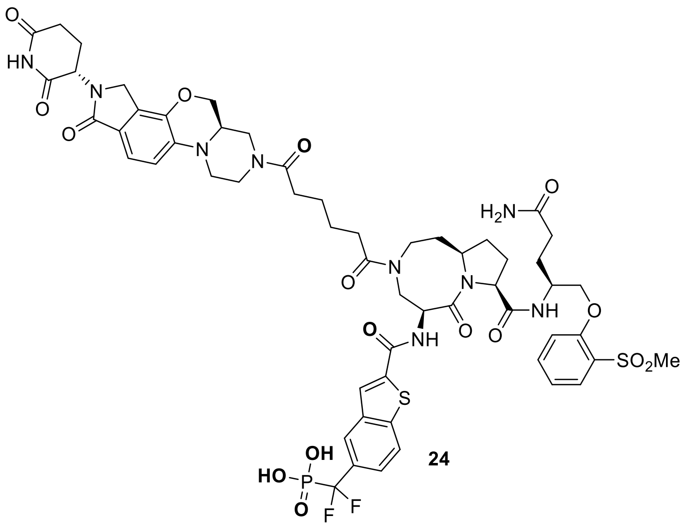

Chemical Formula: $C_{56}H_{64}F_2N_9O_{16}PS_2$
Molecular Weight: 1252.27

SAMPLE INFORMATION

Sample Name:	DW1383-6-2	Acquired By:	System
Sample Type:	Unknown	Sample Set Name:	YQY
Vial:	1:D,7	Acq. Method Set:	10to100Bin10min_5minDelay
Injection #:	1	Processing Method:	Default
Injection Volume:	5.00 ul	Channel Name:	254.0nm
Run Time:	10.0 Minutes	Proc. Chnl. Descr.:	PDA Spectrum PDA 254.0 nm
Date Acquired:	4/24/2025 12:00:47 AM EDT		
Date Processed:	4/24/2025 12:19:10 AM EDT		

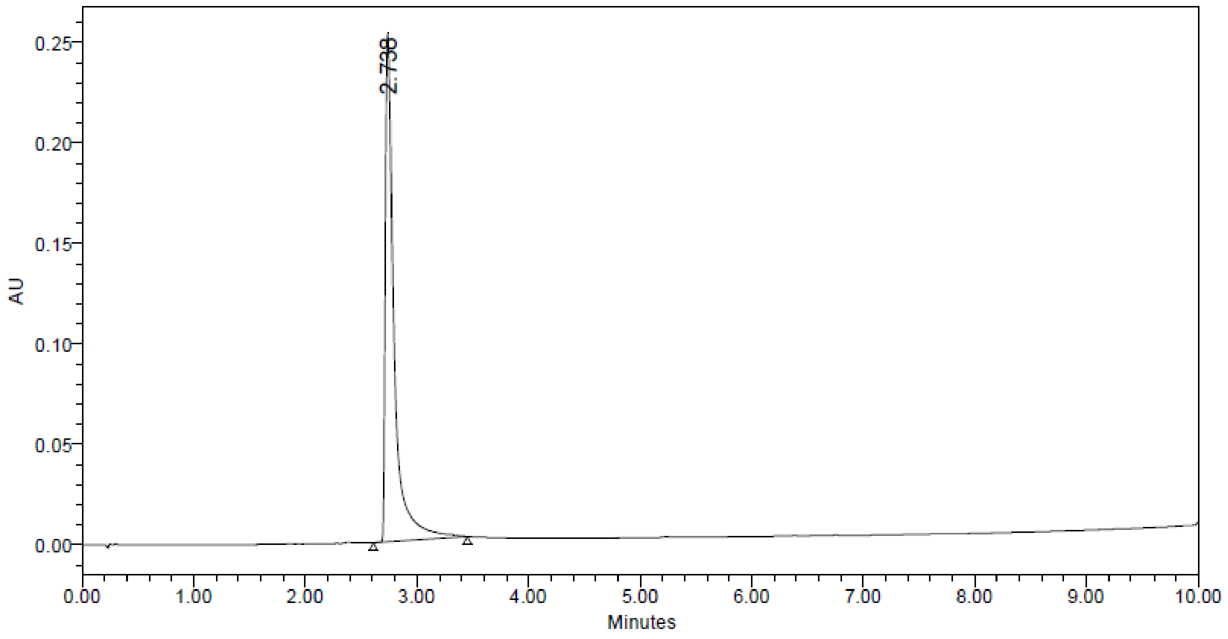

	RT	Area	% Area	Height
1	2.738	1370785	100.00	252837

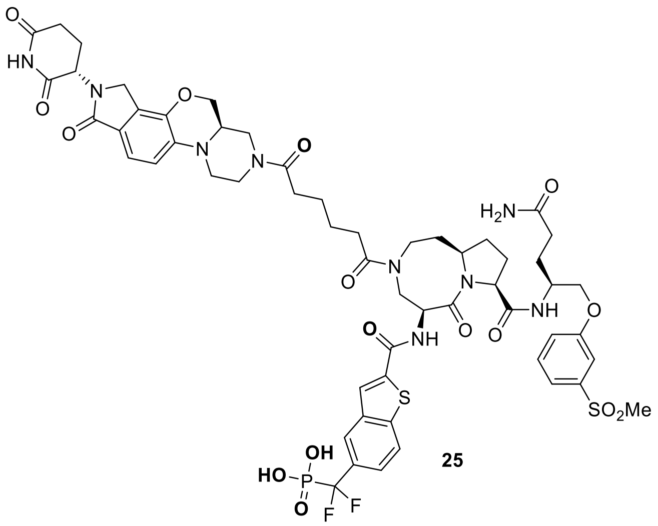

25

Chemical Formula: $C_{56}H_{64}F_2N_9O_{16}PS_2$
Molecular Weight: 1252.27

SAMPLE INFORMATION

Sample Name:	DW1381-2-2	Acquired By:	System
Sample Type:	Unknown	Sample Set Name:	YQY
Vial:	1:D,2	Acq. Method Set:	10to100Bin10min_5minDelay
Injection #:	1	Processing Method:	Default
Injection Volume:	5.00 ul	Channel Name:	254.0nm
Run Time:	10.0 Minutes	Proc. Chnl. Descr.:	PDA Spectrum PDA 254.0 nm
Date Acquired:	4/23/2025 9:57:13 AM EDT		
Date Processed:	4/23/2025 11:02:18 PM EDT		

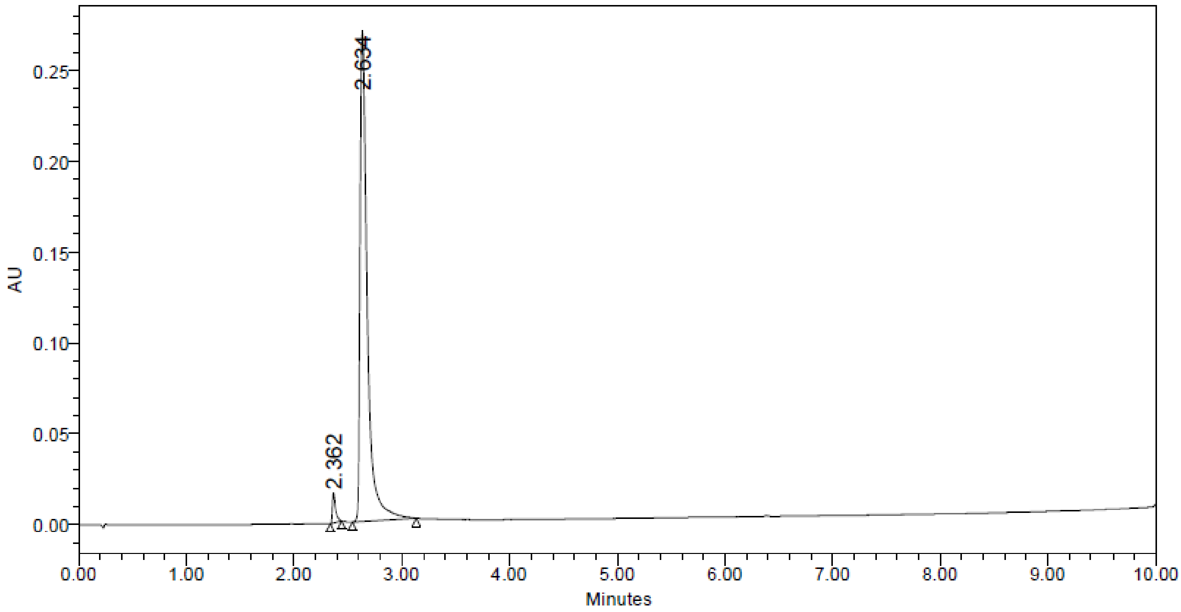

	RT	Area	% Area	Height
1	2.362	33720	2.62	16239
2	2.634	1251711	97.38	269736

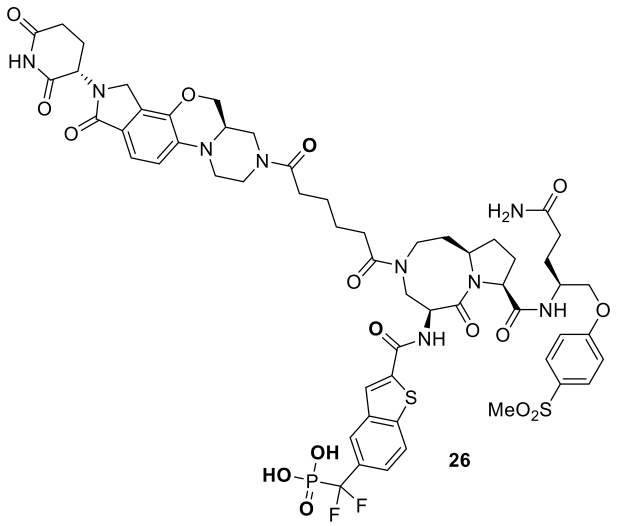

26

Chemical Formula: $C_{56}H_{64}F_2N_9O_{16}PS_2$
Molecular Weight: 1252.27

SAMPLE INFORMATION

Sample Name:	DW1380-7-2	Acquired By:	System
Sample Type:	Unknown	Sample Set Name:	YQY
Vial:	1:C,6	Acq. Method Set:	10to100Bin10min_5minDelay
Injection #:	1	Processing Method:	Default
Injection Volume:	5.00 ul	Channel Name:	254.0nm
Run Time:	10.0 Minutes	Proc. Chnl. Descr.:	PDA Spectrum PDA 254.0 nm
Date Acquired:	4/23/2025 8:54:36 AM EDT		
Date Processed:	4/23/2025 10:59:00 PM EDT		

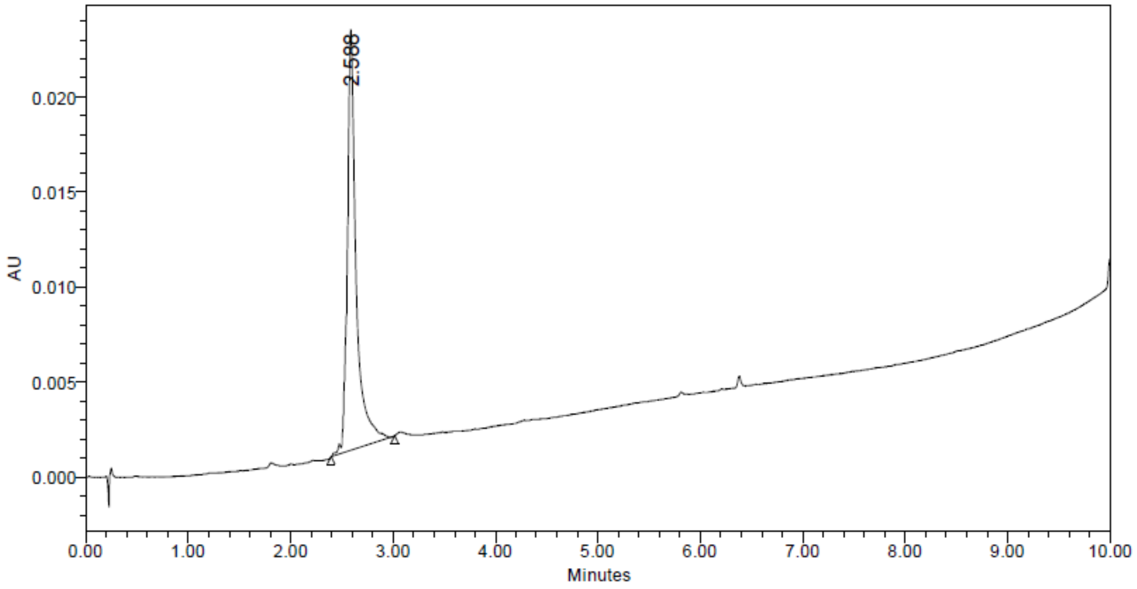

	RT	Area	% Area	Height
1	2.588	132932	100.00	22107

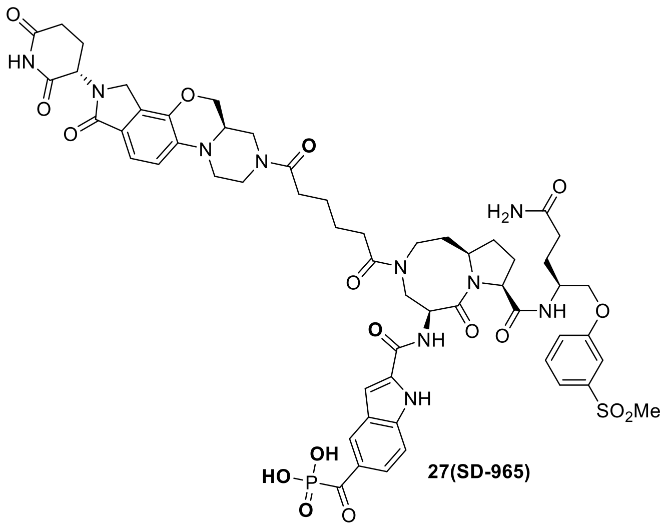

27(SD-965)

Chemical Formula: $C_{56}H_{65}N_{10}O_{17}PS$
Molecular Weight: 1213.22

SAMPLE INFORMATION

Sample Name:	dw1465-6	Acquired By:	System
Sample Type:	Unknown	Date Acquired:	6/21/2025 11:00:19 AM EDT
Vial:	1:E,4	Acq. Method Set:	10to100% Bin 10
Injection #:	1	Date Processed:	6/23/2025 2:41:46 PM EDT
Injection Volume:	5.00 ul	Processing Method:	Bruce
Run Time:	10.0 Minutes	Channel Name:	254.0nm
Sample Set Name:	1	Proc. Chnl. Descr.:	PDA Spectrum (210-500)nm

Auto-Scaled Chromatogram

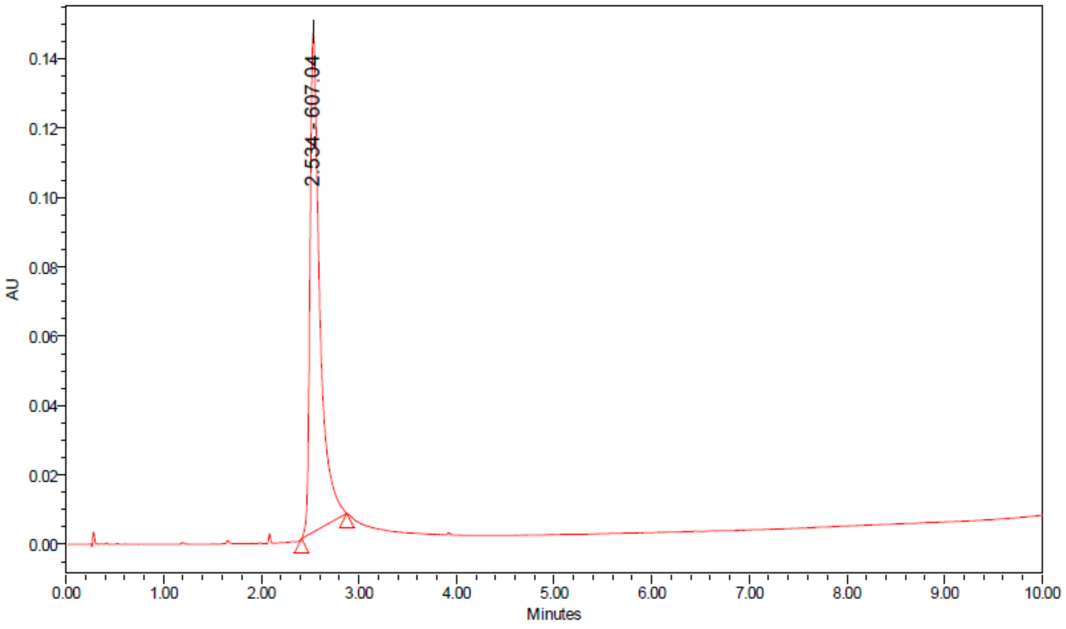

Peak Results

	RT	Area	Height	% Area	Base Peak (m/z)
1	2.534	1010943	144296	100.00	607.04

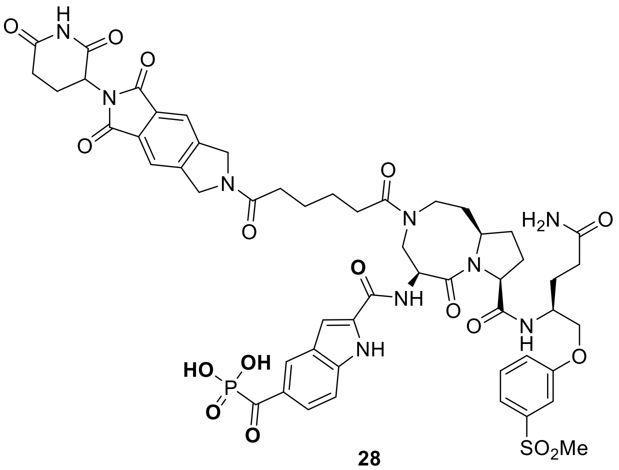

28

Chemical Formula: $C_{53}H_{58}N_9O_{17}PS$

Molecular Weight: 1156.13

SAMPLE INFORMATION			
Sample Name:	dw1416-1-2	Acquired By:	System
Sample Type:	Unknown	Date Acquired:	1/22/2026 12:28:52 AM EST
Vial:	1:D,7	Acq. Method Set:	10to100% Bin 10
Injection #:	1	Date Processed:	1/22/2026 12:45:36 AM EST
Injection Volume:	5.00 ul	Processing Method:	Bruce
Run Time:	10.0 Minutes	Channel Name:	254.0nm
Sample Set Name:	0	Proc. Chnl. Descr.:	PDA Spectrum (210-500)nm

Auto-Scaled Chromatogram

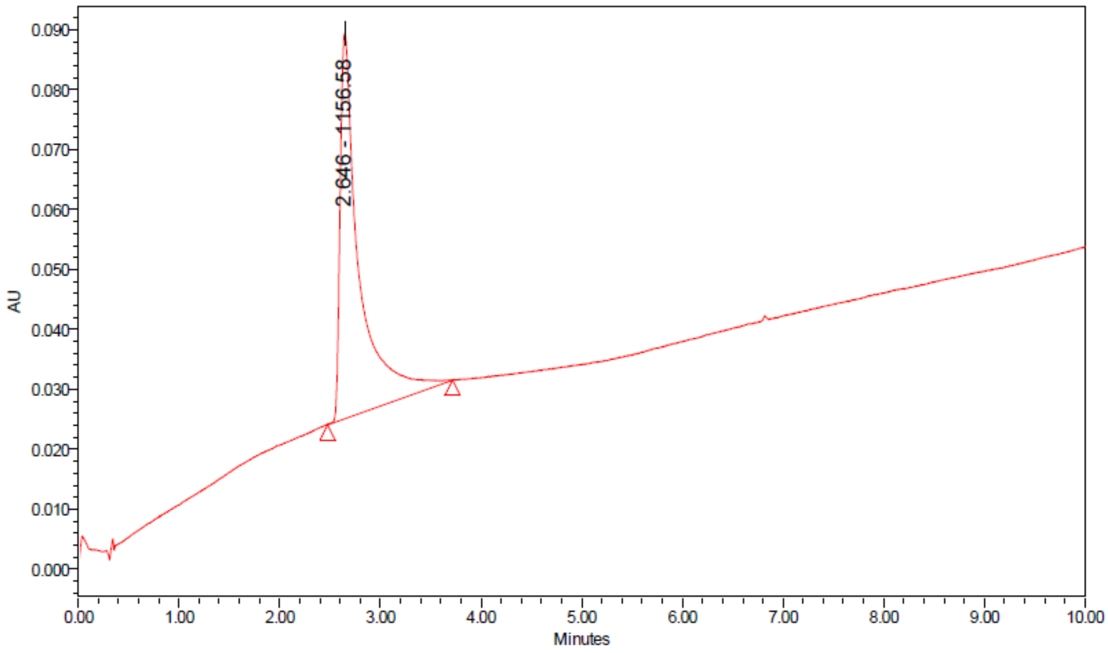

Peak Results

	RT	Area	Height	% Area	Base Peak (m/z)
1	2.646	851134	64313	100.00	1156.58

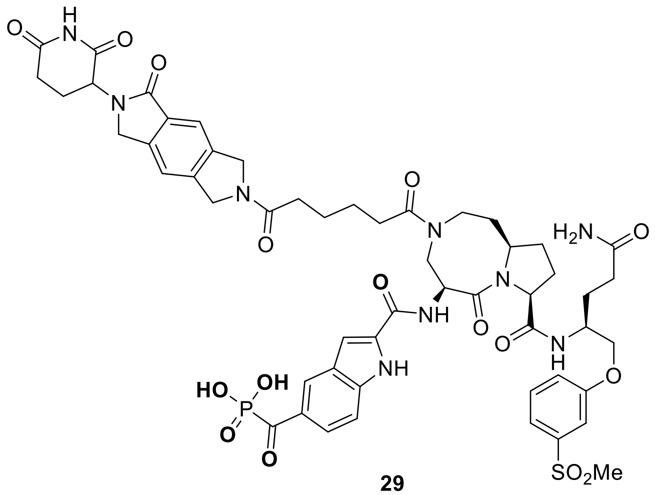

29

Chemical Formula: $C_{53}H_{60}N_9O_{16}PS$

Molecular Weight: 1142.14

SAMPLE INFORMATION

Sample Name:	dw1417-2-2	Acquired By:	System
Sample Type:	Unknown	Date Acquired:	1/21/2026 11:25:43 AM EST
Vial:	1:A,5	Acq. Method Set:	10to100% Bin 10
Injection #:	1	Date Processed:	1/21/2026 10:24:08 PM EST
Injection Volume:	5.00 ul	Processing Method:	Bruce
Run Time:	10.0 Minutes	Channel Name:	254.0nm
Sample Set Name:	0	Proc. Chnl. Descr.:	PDA Spectrum (210-500)nm

Auto-Scaled Chromatogram

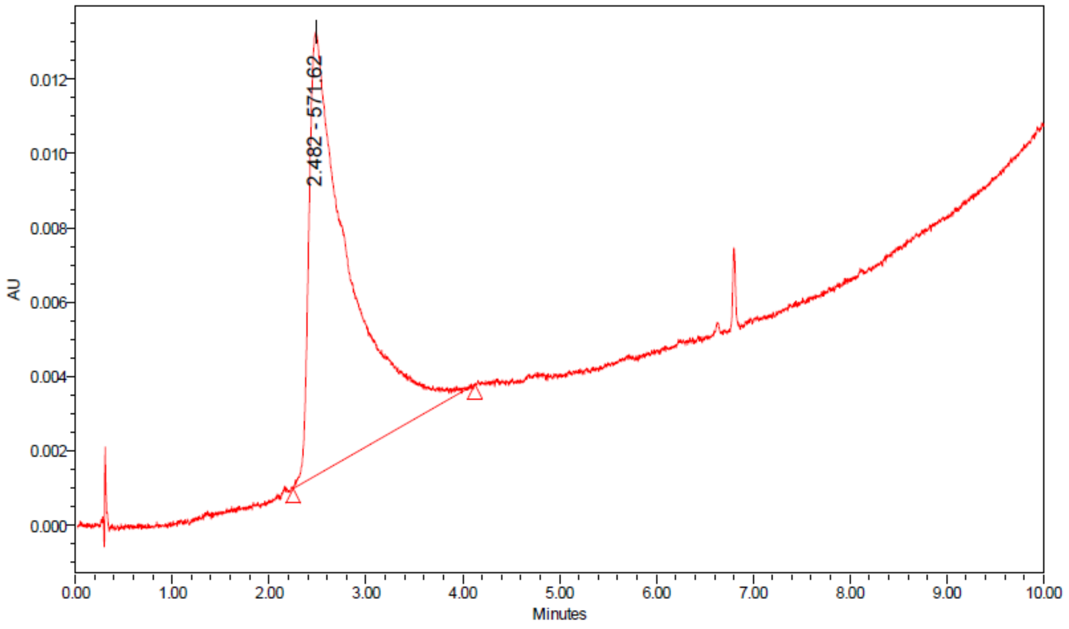

Peak Results

	RT	Area	Height	% Area	Base Peak (m/z)
1	2.482	339151	11934	100.00	571.62

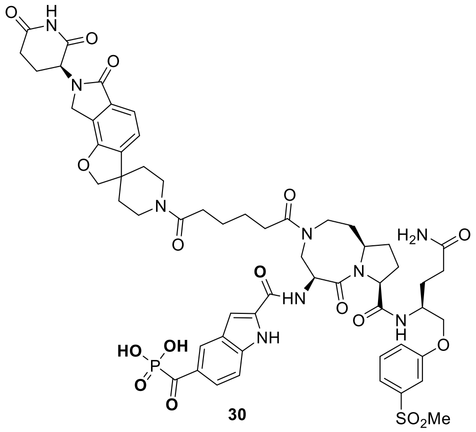

30

Chemical Formula: $C_{57}H_{66}N_9O_{17}PS$

Molecular Weight: 1212.23

SAMPLE INFORMATION

Sample Name:	dw1418-4-2	Acquired By:	System
Sample Type:	Unknown	Date Acquired:	1/21/2026 9:57:37 AM EST
Vial:	1:F,8	Acq. Method Set:	10to100% Bin 10
Injection #:	1	Date Processed:	[illegible] 1/21/2026 10:24:52 AM EST
Injection Volume:	5.00 ul	Processing Method:	Bruce
Run Time:	10.0 Minutes	Channel Name:	254.0nm
Sample Set Name:	0	Proc. Chnl. Descr.:	PDA Spectrum (210-500)nm

Auto-Scaled Chromatogram

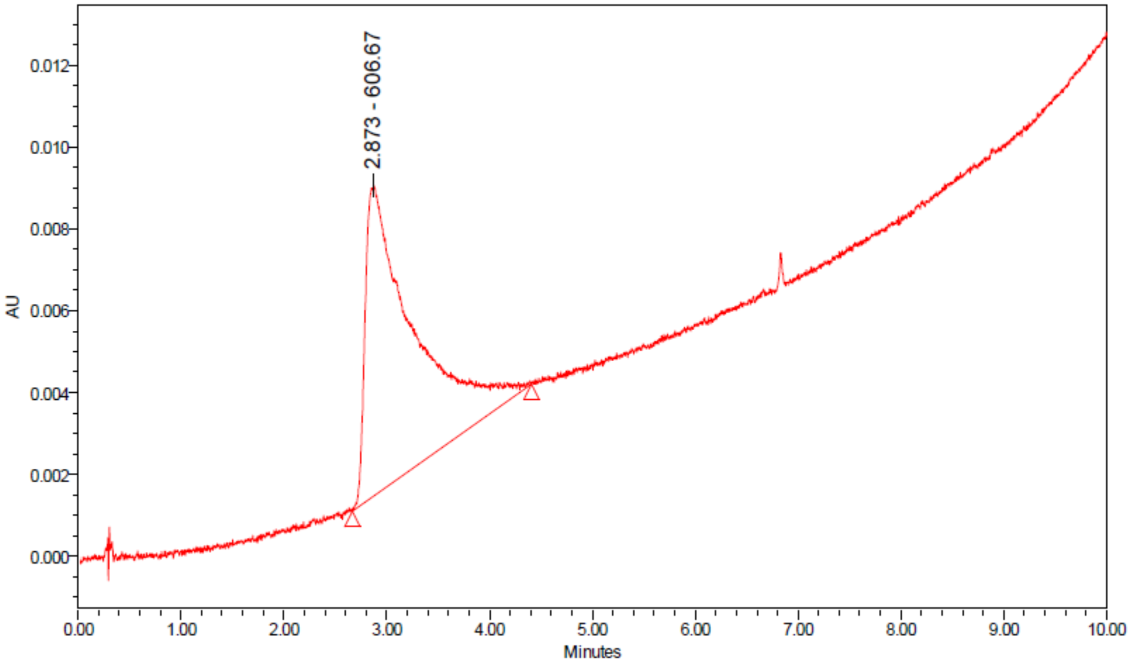

Peak Results

	RT	Area	Height	% Area	Base Peak (m/z)
1	2.873	246730	7590	100.00	606.67

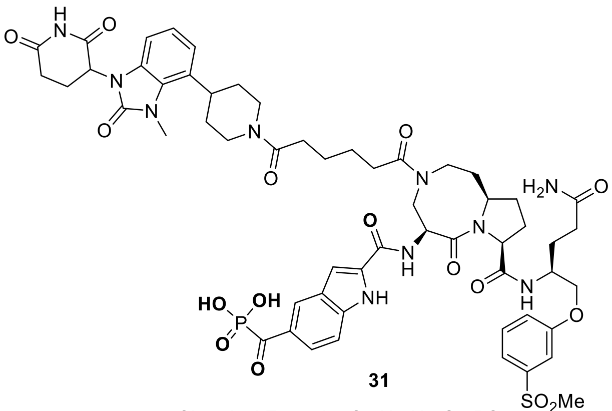

31

Chemical Formula: $C_{56}H_{67}N_{10}O_{16}PS$
Molecular Weight: 1199.24

SAMPLE INFORMATION

Sample Name:	DW1419-T	Acquired By:	System
Sample Type:	Unknown	Date Acquired:	7/1/2025 4:25:01 PM EDT
Vial:	1:B,1	Acq. Method Set:	10to100% Bin 10
Injection #:	1	Date Processed:	7/1/2025 5:00:38 PM EDT
Injection Volume:	5.00 ul	Processing Method:	Bruce
Run Time:	10.0 Minutes	Channel Name:	254.0nm@2
Sample Set Name:	7	Proc. Chnl. Descr.:	PDA Spectrum (210-500)nm

Auto-Scaled Chromatogram

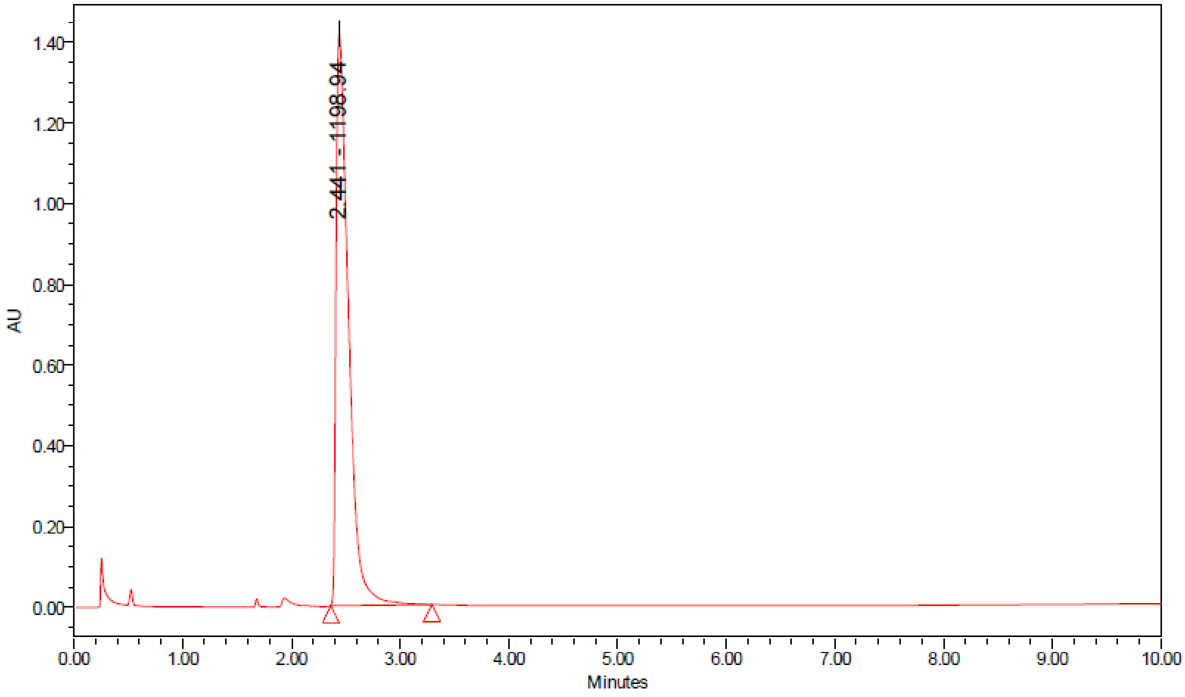

Peak Results

	RT	Area	Height	% Area	Base Peak (m/z)
1	2.441	11198506	1417987	100.00	1198.94

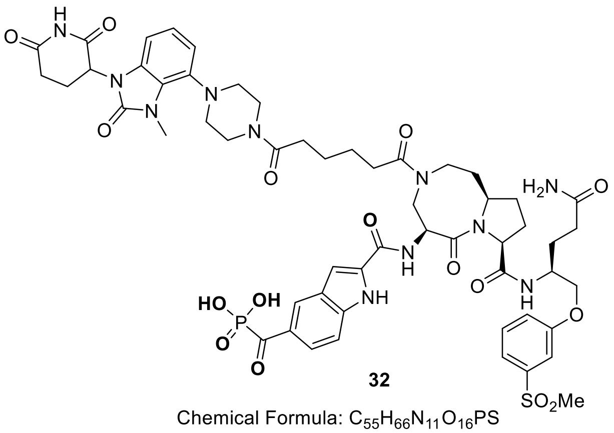

32

Chemical Formula: $C_{55}H_{66}N_{11}O_{16}PS$
Molecular Weight: 1200.23

SAMPLE INFORMATION

Sample Name:	DW1420-3	Acquired By:	System
Sample Type:	Unknown	Date Acquired:	8/22/2025 9:03:08 AM EDT
Vial:	1:D,1	Acq. Method Set:	10to100% Bin 10
Injection #:	1	Date Processed:	8/22/2025 11:19:11 AM EDT m/z_Delay5min
Injection Volume:	8.00 ul	Processing Method:	Bruce
Run Time:	10.0 Minutes	Channel Name:	254.0nm
Sample Set Name:	1	Proc. Chnl. Descr.:	PDA Spectrum (210-500)nm

Auto-Scaled Chromatogram

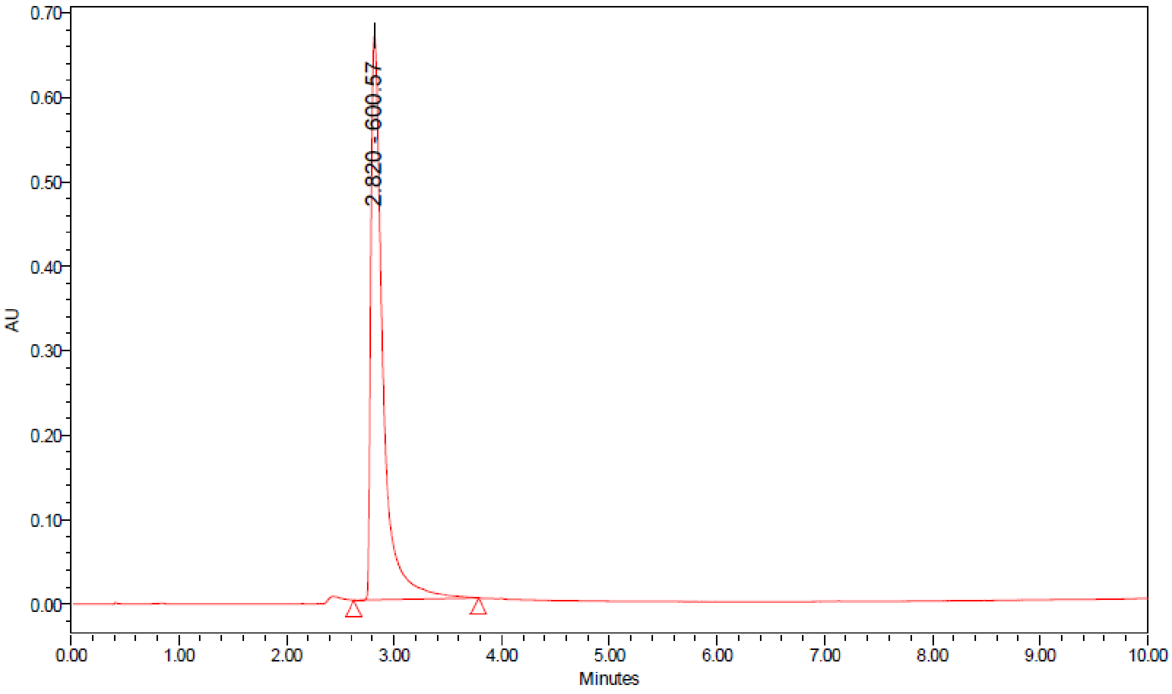

Peak Results

	RT	Area	Height	% Area	Base Peak (m/z)
1	2.820	5145069	668643	100.00	600.57

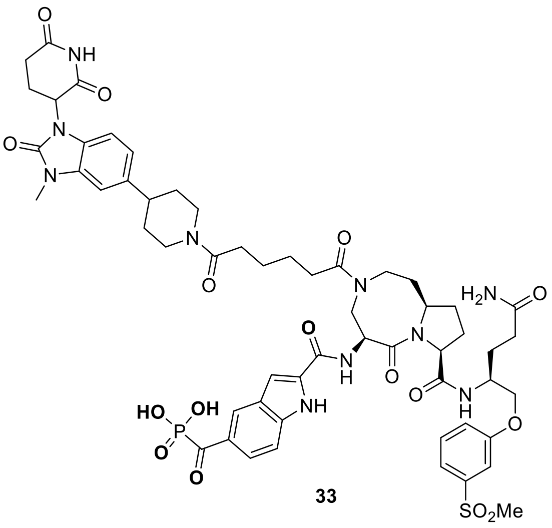

33

Chemical Formula: $C_{56}H_{67}N_{10}O_{16}PS$
Molecular Weight: 1199.24

SAMPLE INFORMATION			
Sample Name:	DW1425-8	Acquired By:	System
Sample Type:	Unknown	Date Acquired:	8/21/2025 10:24:06 AM EDT
Vial:	1:C,1	Acq. Method Set:	10to100% Bin 10
Injection #:	1	Date Processed:	8/21/2025 11:15:28 AM EDT
Injection Volume:	8.00 ul	Processing Method:	Bruce
Run Time:	10.0 Minutes	Channel Name:	254.0nm
Sample Set Name:	1	Proc. Chnl. Descr.:	PDA Spectrum (210-500)nm

Auto-Scaled Chromatogram

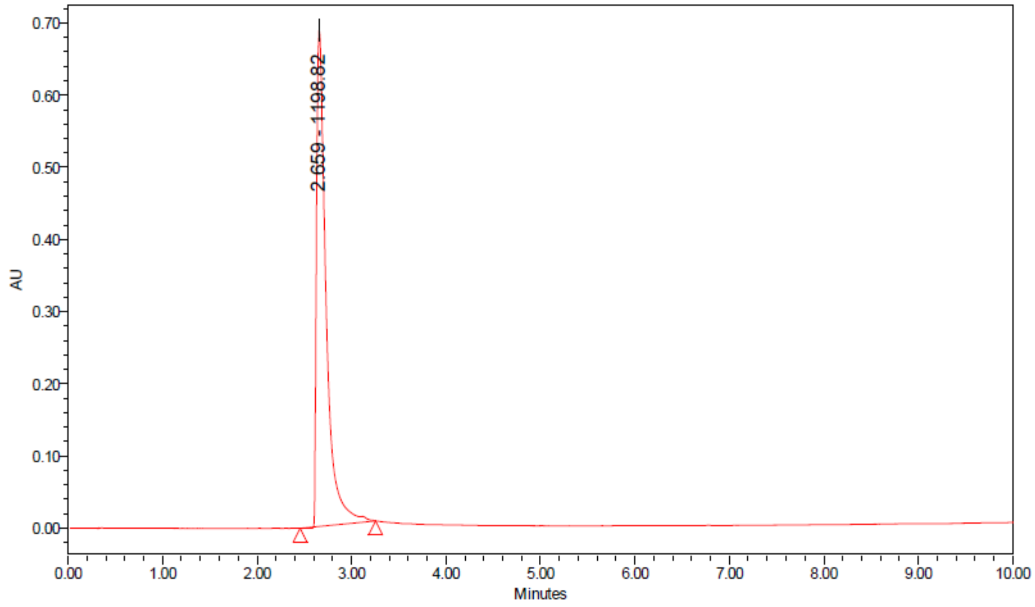

Peak Results

	RT	Area	Height	% Area	Base Peak (m/z)
1	2.659	4866306	687834	100.00	1198.82

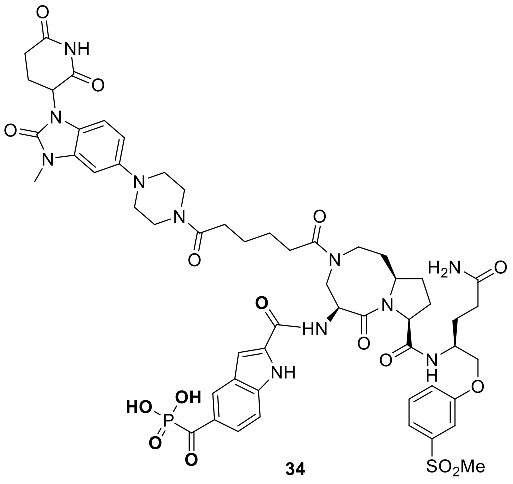

34

Chemical Formula: $C_{55}H_{66}N_{11}O_{16}PS$

Molecular Weight: 1200.23

SAMPLE INFORMATION

Sample Name:	dw1428-3-1	Acquired By:	System
Sample Type:	Unknown	Date Acquired:	2/5/2026 10:54:10 AM EST
Vial:	1:F,4	Acq. Method Set:	10to100% Bin 10
Injection #:	1	Date Processed:	2/5/2026 [illegible] 18 PM EST
Injection Volume:	5.00 ul	Processing Method:	Bruce
Run Time:	10.0 Minutes	Channel Name:	254.0nm
Sample Set Name:	1	Proc. Chnl. Descr.:	PDA Spectrum (210-500)nm

Auto-Scaled Chromatogram

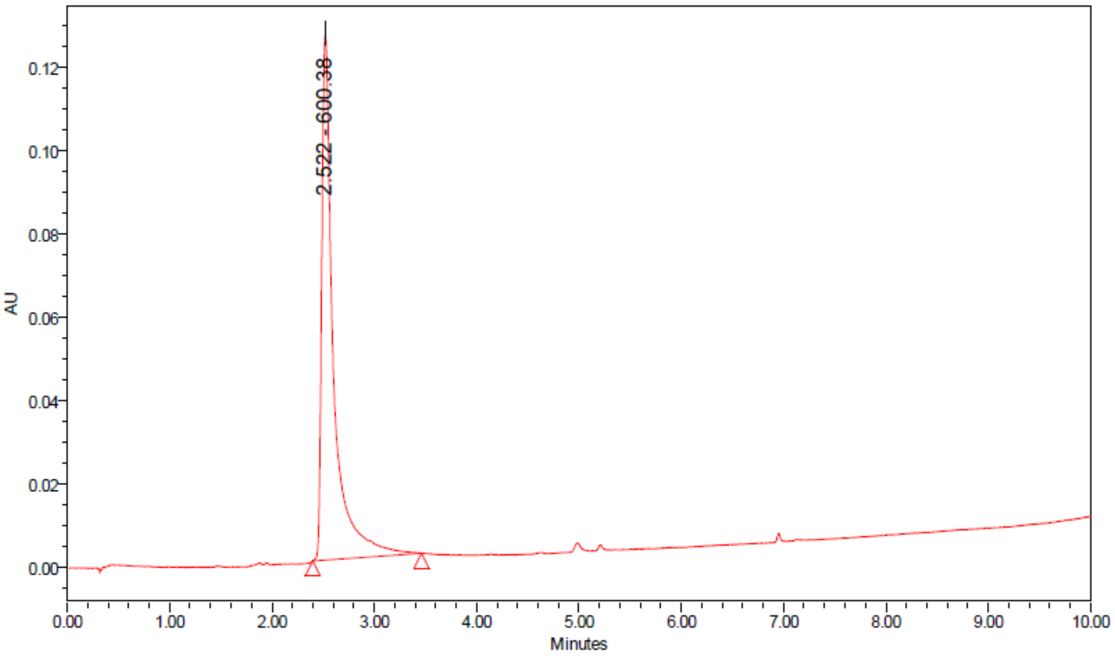

Peak Results

	RT	Area	Height	% Area	Base Peak (m/z)
1	2.522	1017013	126421	100.00	600.38

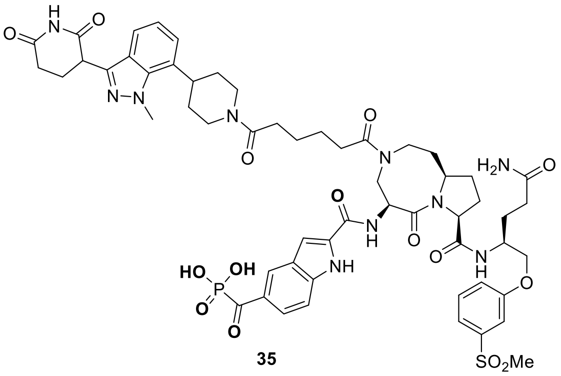

35

Chemical Formula: $C_{56}H_{67}N_{10}O_{15}PS$
Molecular Weight: 1183.24

SAMPLE INFORMATION			
Sample Name:	dw1424-1-2	Acquired By:	System
Sample Type:	Unknown	Date Acquired:	1/21/2026 3:32:13 PM EST
Vial:	1:B,6	Acq. Method Set:	10to100% Bin 10
Injection #:	1	Date Processed:	1/21/2026 4:04:21 PM EST
Injection Volume:	5.00 ul	Processing Method:	Bruce
Run Time:	10.0 Minutes	Channel Name:	254.0nm
Sample Set Name:	0	Proc. Chnl. Descr.:	PDA Spectrum (210-500)nm

Auto-Scaled Chromatogram

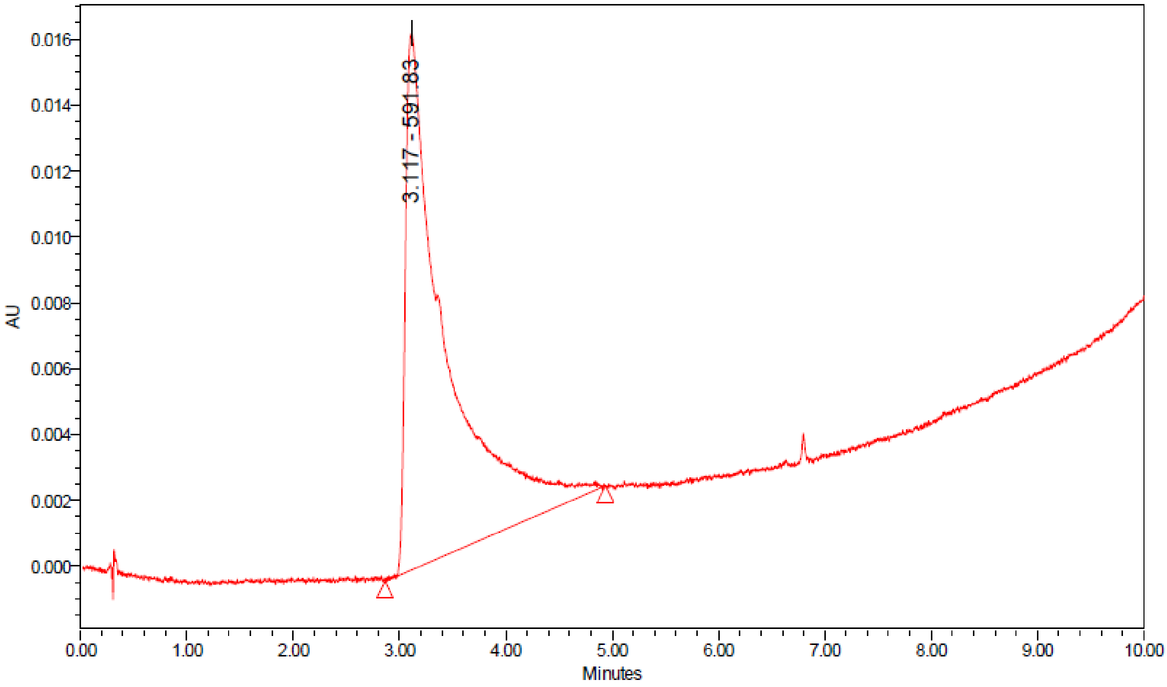

Peak Results

	RT	Area	Height	% Area	Base Peak (m/z)
1	3.117	428808	16303	100.00	591.83

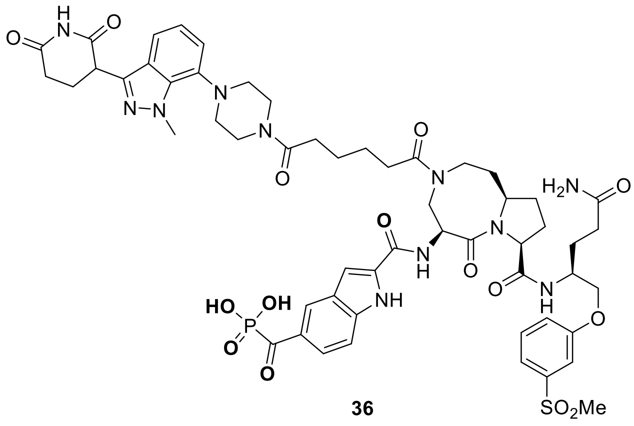

36

Chemical Formula: $C_{55}H_{66}N_{11}O_{15}PS$
Molecular Weight: 1184.23

SAMPLE INFORMATION

Sample Name:	dw1423-1-5	Acquired By:	System
Sample Type:	Unknown	Date Acquired:	1/21/2026 3:00:51 PM EST
Vial:	1:B,4	Acq. Method Set:	10to100% Bin 10
Injection #:	1	Date Processed:	m/a_Delay5min 1/21/2026 4:01:25 PM EST
Injection Volume:	5.00 ul	Processing Method:	Bruce
Run Time:	10.0 Minutes	Channel Name:	254.0nm
Sample Set Name:	0	Proc. Chnl. Descr.:	PDA Spectrum (210-500)nm

Auto-Scaled Chromatogram

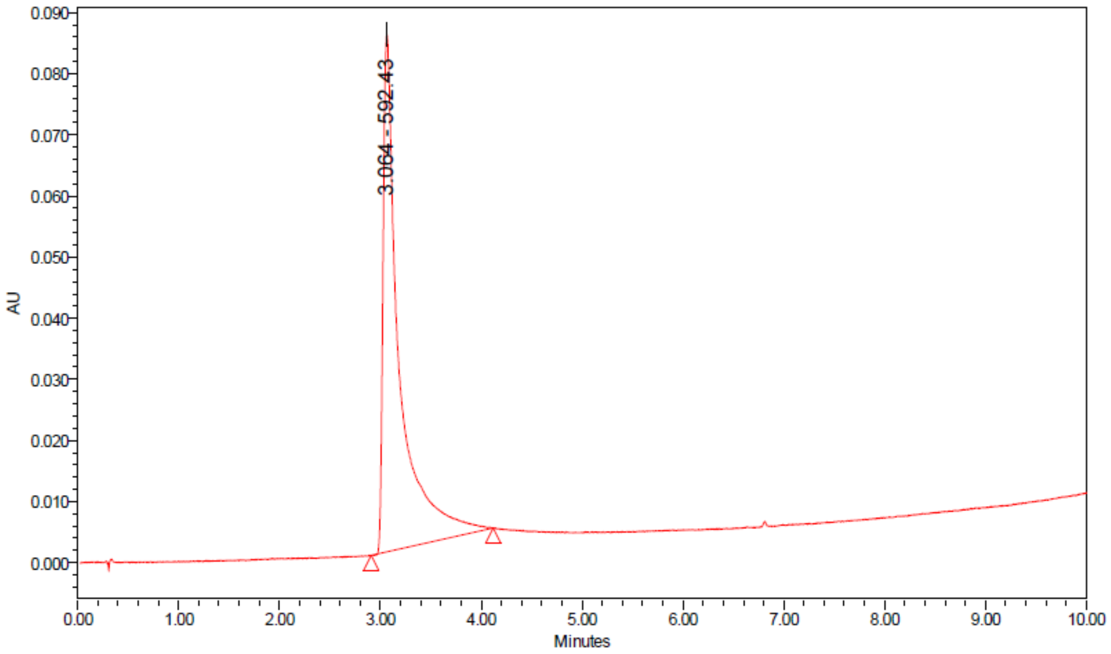

Peak Results

	RT	Area	Height	% Area	Base Peak (m/z)
1	3.064	959100	84664	100.00	592.43

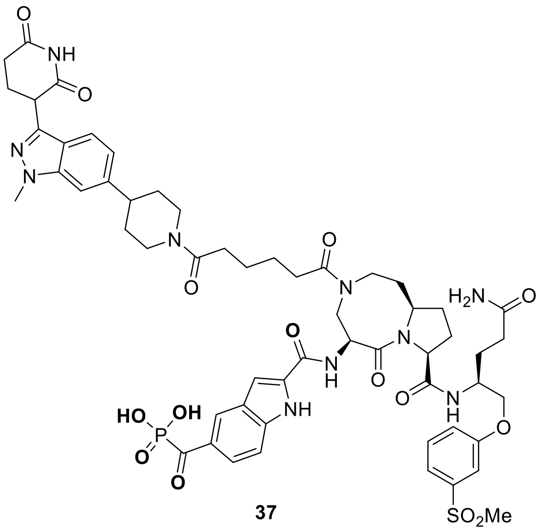

37

Chemical Formula: $C_{56}H_{67}N_{10}O_{15}PS$
Molecular Weight: 1183.24

SAMPLE INFORMATION

Sample Name:	DW1421-4-1	Acquired By:	System
Sample Type:	Unknown	Date Acquired:	1/20/2026 11:16:56 AM EST
Vial:	1:D,1	Acq. Method Set:	10to100% Bin 10 min_Delay5min
Injection #:	1	Date Processed:	1/20/2026 5:04:13 PM EST
Injection Volume:	5.00 ul	Processing Method:	Bruce
Run Time:	10.0 Minutes	Channel Name:	254.0nm
Sample Set Name:	1	Proc. Chnl. Descr.:	PDA Spectrum (210-500)nm

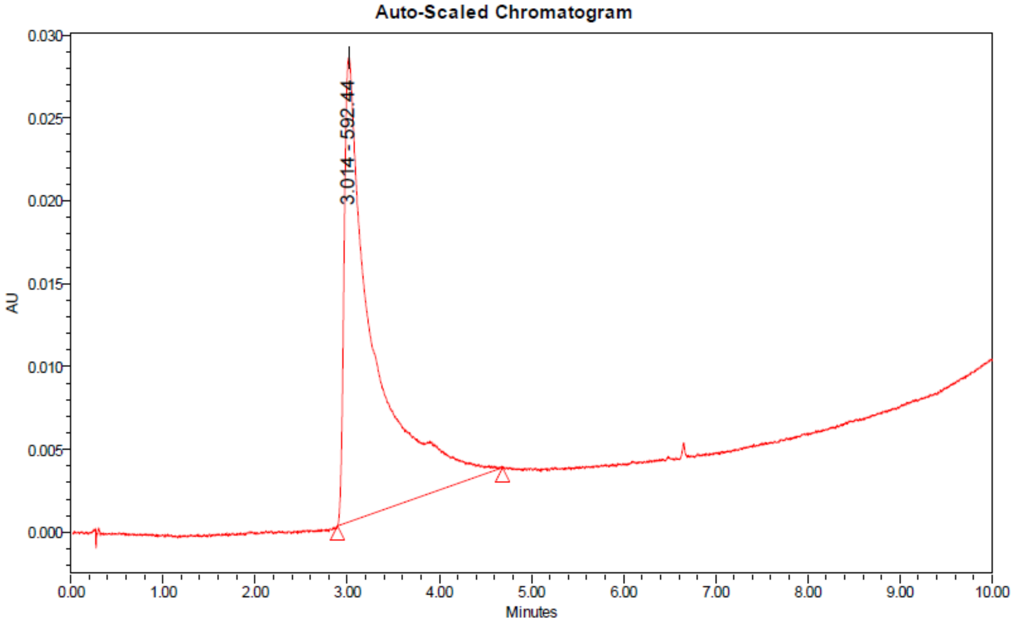

Peak Results

	RT	Area	Height	% Area	Base Peak (m/z)
1	3.014	617004	28013	100.00	592.44

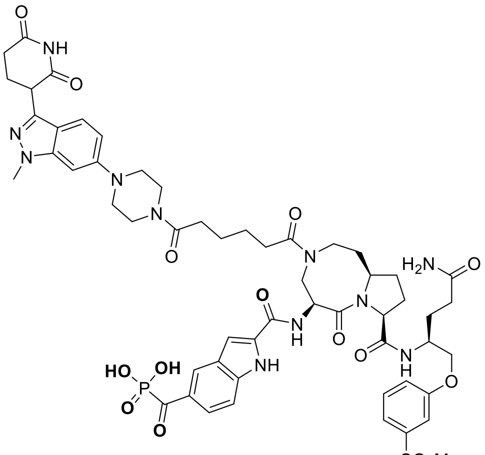

38

Chemical Formula: $C_{55}H_{66}N_{11}O_{15}PS$

Molecular Weight: 1184.23

SAMPLE INFORMATION

Sample Name:	dw1422-2-2	Acquired By:	System
Sample Type:	Unknown	Date Acquired:	1/22/2026 12:00:08 AM EST
Vial:	1:D,6	Acq. Method Set:	10to100% Bin 10
Injection #:	1	Date Processed:	1/22/2026 12:07:32 AM EST
Injection Volume:	5.00 ul	Processing Method:	Bruce
Run Time:	10.0 Minutes	Channel Name:	254.0nm
Sample Set Name:	0	Proc. Chnl. Descr.:	PDA Spectrum (210-500)nm

Auto-Scaled Chromatogram

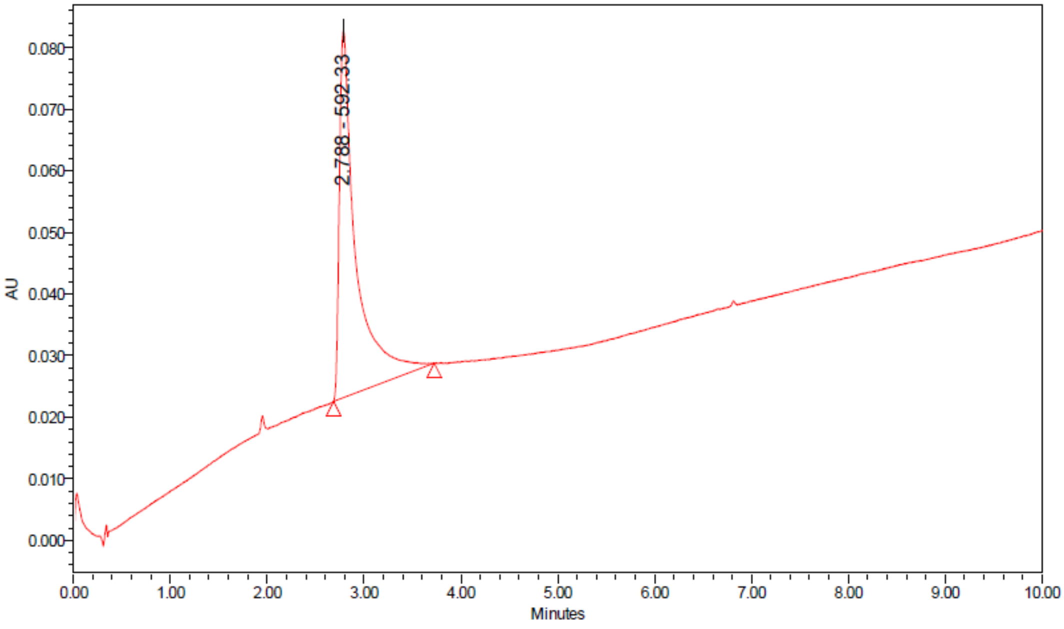

Peak Results

	RT	Area	Height	% Area	Base Peak (m/z)
1	2.788	711777	59539	100.00	592.33

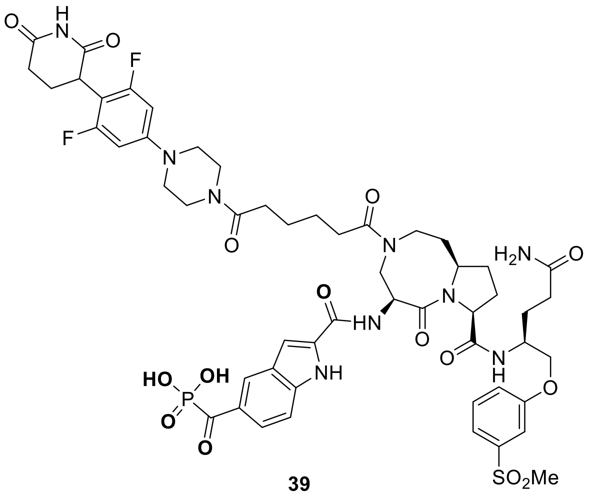

39

Chemical Formula: $C_{53}H_{62}F_2N_9O_{15}PS$
Molecular Weight: 1166.16

SAMPLE INFORMATION

Sample Name:	dw1426-5-3	Acquired By:	System
Sample Type:	Unknown	Sample Set Name:	PG 01
Vial:	1:A,2	Acq. Method Set:	10to100Bin10min_5minDelay
Injection #:	1	Processing Method:	Dimin
Injection Volume:	5.00 ul	Channel Name:	254.0nm
Run Time:	10.0 Minutes	Proc. Chnl. Descr.:	PDA Spectrum PDA 254.0 nm
Date Acquired:	2/10/2026 6:38:48 AM EST		
Date Processed:	2/10/2026 6:59:21 AM EST		

Auto-Scaled Chromatogram

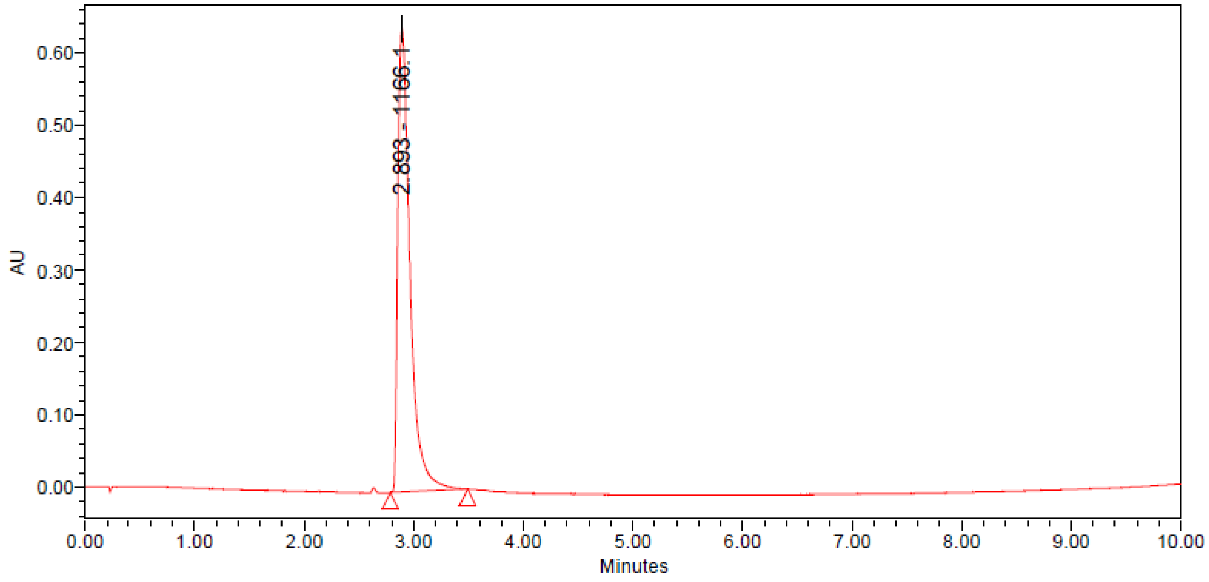

Peak Results

	Name	RT	Area	Height	Amount	Units
1		2.893	4895006	639893		

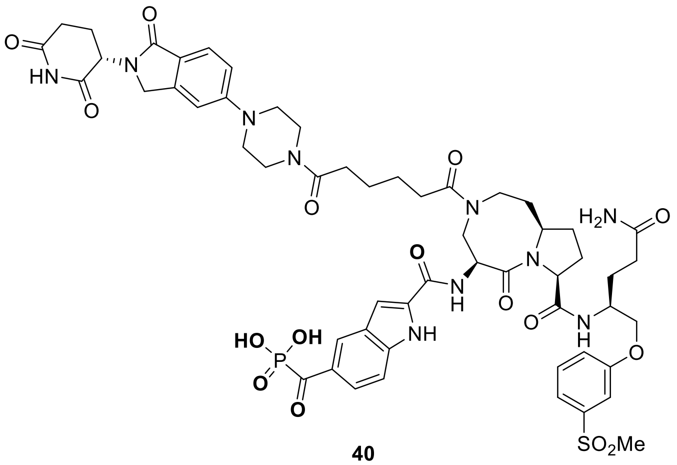

40

Chemical Formula: $C_{55}H_{65}N_{10}O_{16}PS$

Molecular Weight: 1185.21

SAMPLE INFORMATION

Sample Name:	dw1427	Acquired By:	System
Sample Type:	Unknown	Sample Set Name:	PG 01
Vial:	1:E,8	Acq. Method Set:	10to100Bin10min_5minDelay
Injection #:	1	Processing Method:	Dimin
Injection Volume:	5.00 ul	Channel Name:	254.0nm
Run Time:	10.0 Minutes	Proc. Chnl. Descr.:	PDA Spectrum PDA 254.0 nm
Date Acquired:	2/9/2026 11:24:50 PM EST		
Date Processed:	2/9/2026 11:48:01 PM EST		

Auto-Scaled Chromatogram

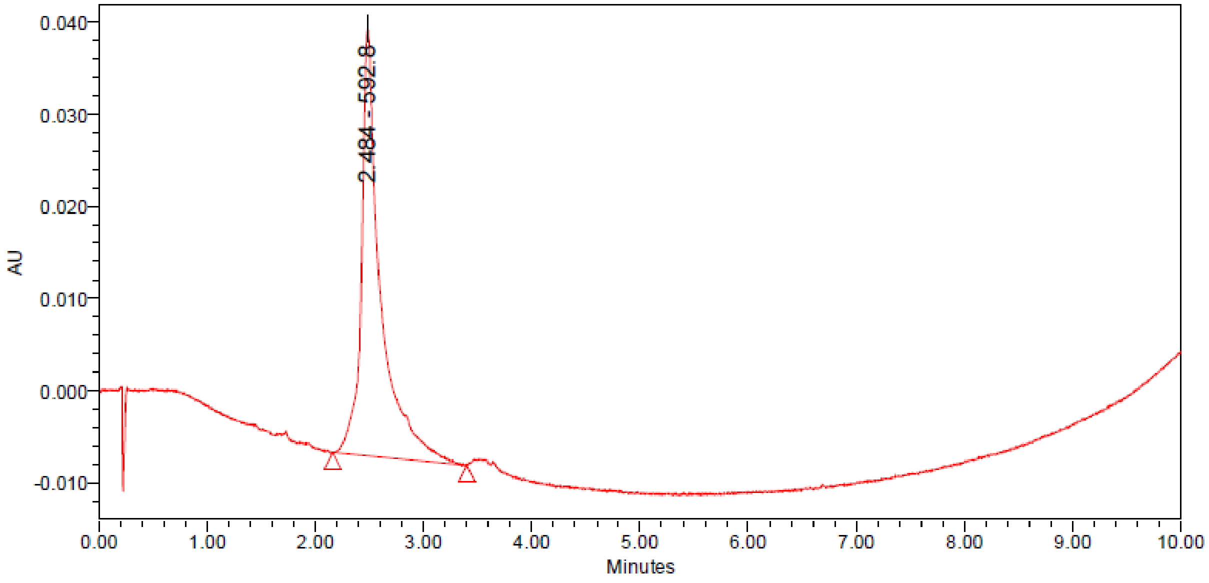

Peak Results

	Name	RT	Area	Height	Amount	Units
1		2.484	568302	46341		

4. **Figure S4.** NMR spectra of representative STAT3 degraders

^{1}H NMR(400 MHz, DMSO-d6/D_2O) of **27** (SD-965):

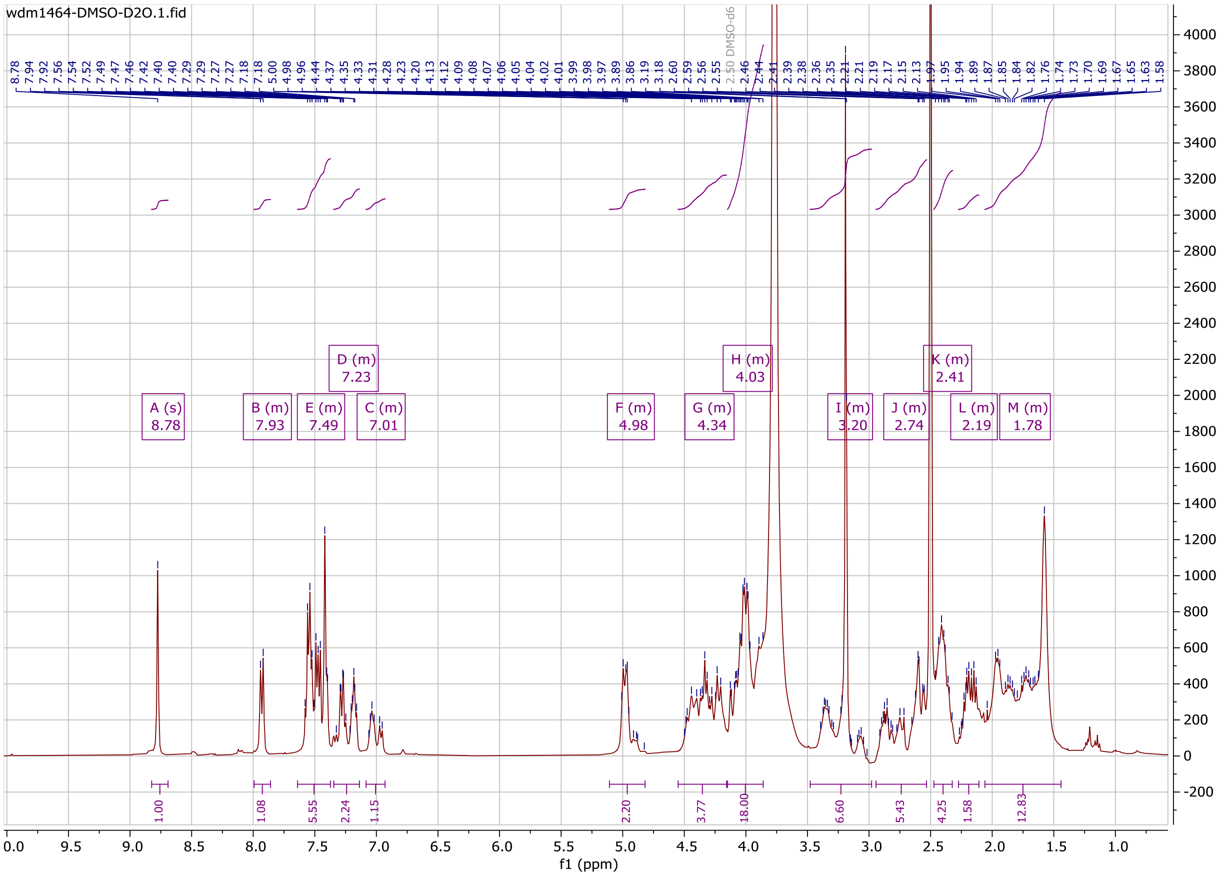

^{13}C NMR(600 MHz, DMSO-d6/D_2O) of **27** (SD-965):

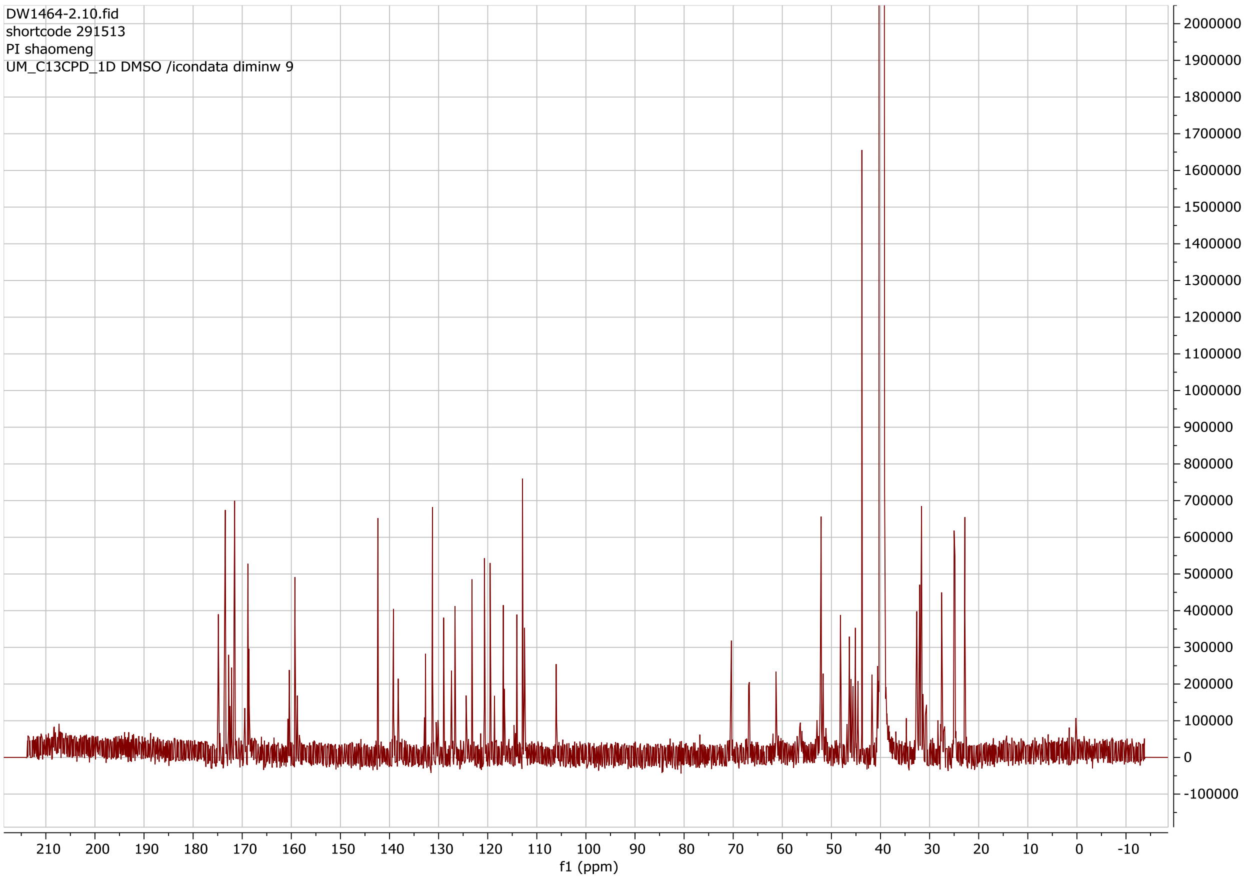

^{1}H NMR(400 MHz, DMSO-d6/D_2O) of **25**:

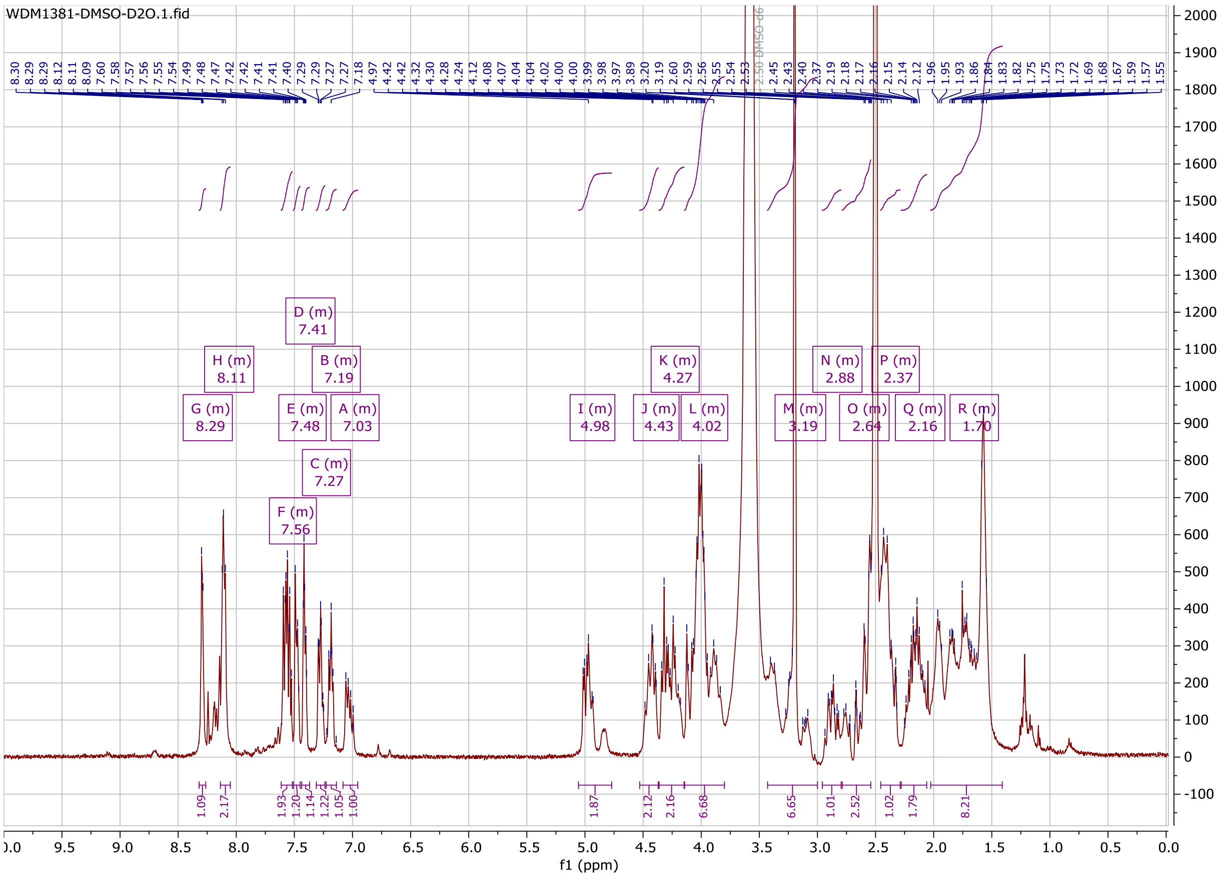

^{1}H NMR(400 MHz, DMSO-d6/D_2O) of **31**:

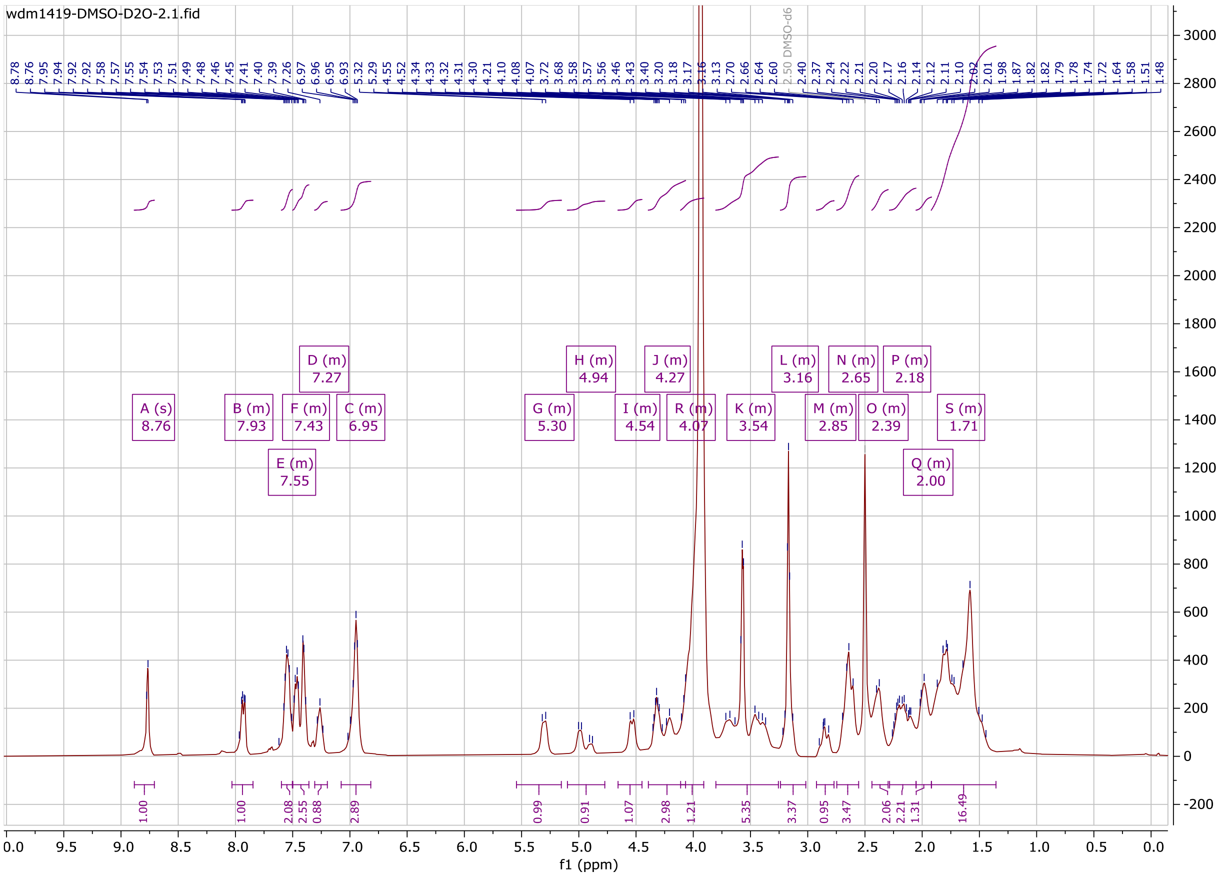

^{1}H NMR(400 MHz, DMSO-d6/D_2O) of **32**:

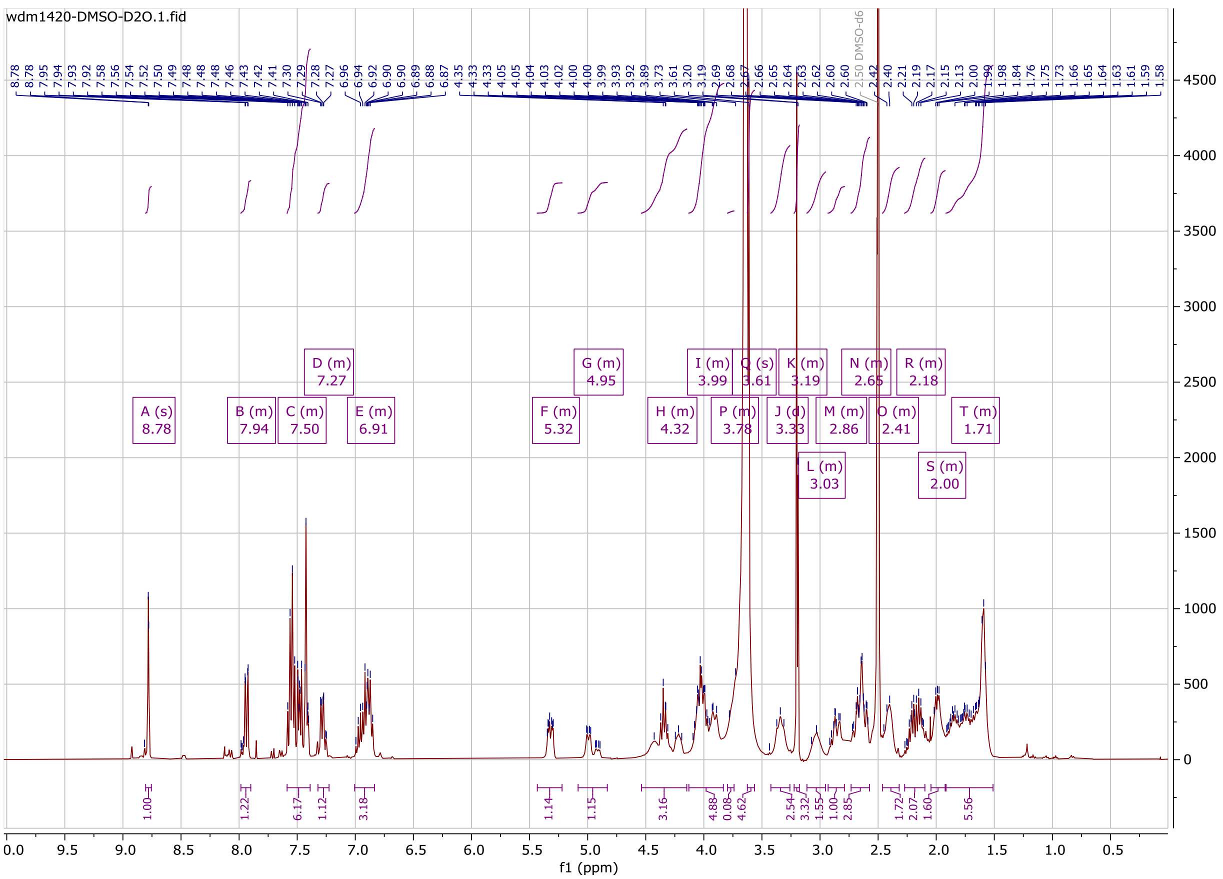

5. **Figure S5**. Our previously reported co-crystal structure of SD-36 with STAT3 (PDB ID: 6NJS). Three residues in STAT3 interacting with the pro-(S)-phenyl group in SD-36 were highlighted.

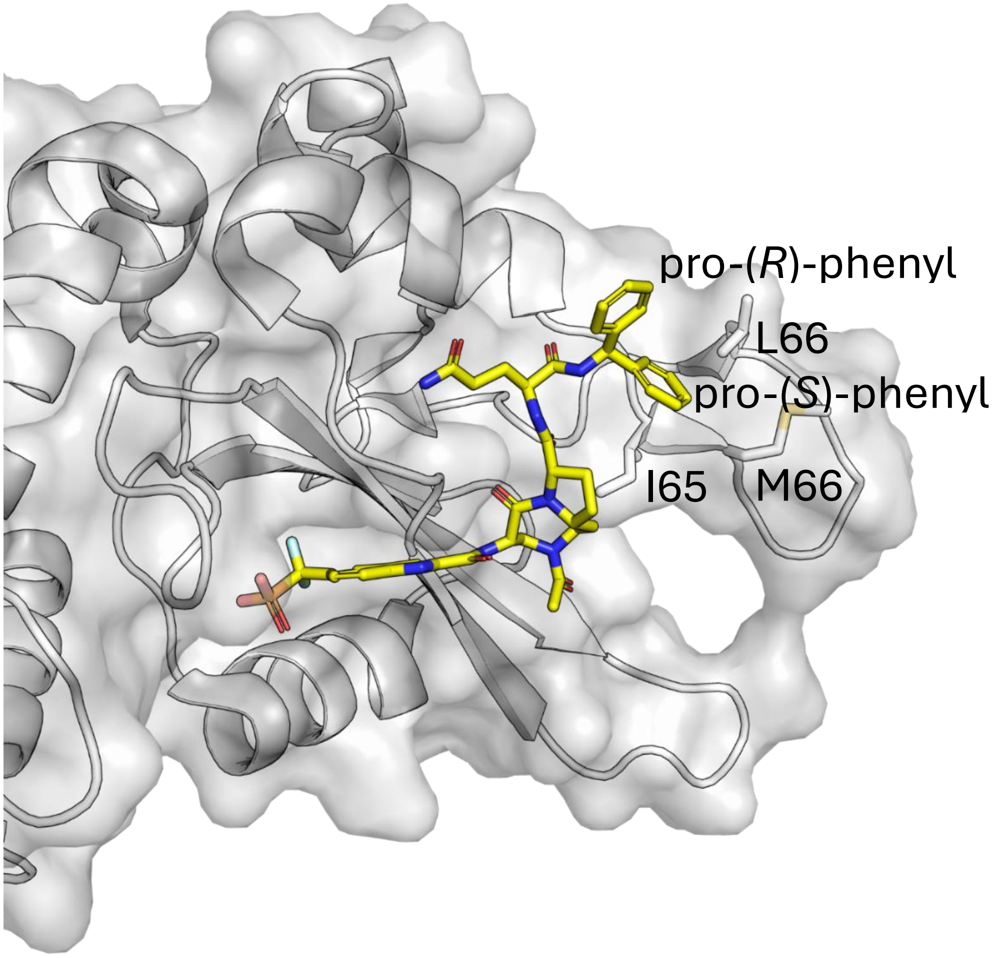

Supplement: Supplementary file 1 [file jm5c03767_si_001.pdf]
